# Cancer treatment alters mutant selection in normal esophagus

**DOI:** 10.1038/s41588-026-02738-0

**Published:** 2026-09-11

**Authors:** Joanna C. Fowler, Giuseppina Arbore, Roshan K. Sood, Irina Abnizova, Luca Albarello, Oliver Pickering, Kasumi Murai, Ujjwal Banerjee, Salomé Brunon, Swee Hoe Ong, Andrea Cossu, Ugo Elmore, Francesco Puccetti, David Fernandez-Antoran, Giovanni Tonon, Paolo Dellabona, Riccardo Rosati, Samuel Luke Hill, Tim Underwood, Benjamin A. Hall, David Shorthouse, Philip H. Jones

**Affiliations:** 1 Wellcome Sanger Institute, Hinxton, UK; 2 Experimental Immunology Unit, IRCCS San Raffaele Scientific Institute, Milan, Italy; 3 Vita-Salute San Raffaele University, Milan, Italy; 4 Department of Pathology, IRCCS San Raffaele Scientific Institute, Milan, Italy; 5 School of Cancer Sciences, Faculty of Medicine, University of Southampton, Southampton, UK; 6 Department of Gastrointestinal Surgery, IRCCS San Raffaele Scientific Institute, Milan, Italy; 7 The Gurdon Institute, University of Cambridge, Cambridge, UK; 8 Aragón Health Research Institute (IIS-Aragón), Zaragoza, Spain; 9 Aragonese Agency for Research and Development Foundation (ARAID), Zaragoza, Spain; 10 Center for OMICS Sciences, IRCCS San Raffaele Scientific Institute, Milan, Italy; 11 Department of Medical Physics and Biomedical Engineering, University College London, London, UK; 12 School of Pharmacy, University College London, London, UK; 13 Department of Oncology, University of Cambridge, Hutchison Research Centre, Cambridge Biomedical Campus, Cambridge, UK

**Keywords:** Oesophageal cancer, Genomics, Genome informatics

## Abstract

Aging epithelial tissues, including the esophagus, are colonized by somatic mutant clones under strong competitive selection. The effect of cancer treatment on mutant selection in normal epithelium is unknown. We hypothesized that some mutant clones may be selectively expanded during treatment. To test this, we sequenced normal esophageal epithelium removed from 70 patients after therapy for esophageal cancer. Patients received either no treatment, combination chemotherapy (FLOT, ECX or EOX) or chemotherapy and radiation therapy (CROSS). Mutant *TP53* and *PPM1D* clones were expanded in patients undergoing CROSS. In the group undergoing FLOT, there was increased selection for *RAC1*, *NFE2L2* and *MTOR* mutations consistent with these mutants conferring 5-fluorouracil resilience in normal epithelium. Sequencing normal epithelia reveals treatment-specific selection of mutations and may identify genes implicated in cellular responses to therapy.

## Abstract (web summary)

Cancer treatment alters the selection of preexisting somatic mutations in the normal esophagus, including druggable mutants that confer treatment resilience to normal cells.

## Main

Normal esophageal epithelium is an evolving patchwork of mutant clones^1–3^. Mutational signature analysis indicates that esophageal mutations are generated by cell-intrinsic processes and, in some individuals, alcohol and tobacco exposure^1,2,4^. Mutant clones with increased fitness expand, collide and compete for space in the tissue, with only the fittest mutations surviving^3,5,6^.

**Fig. 1:**
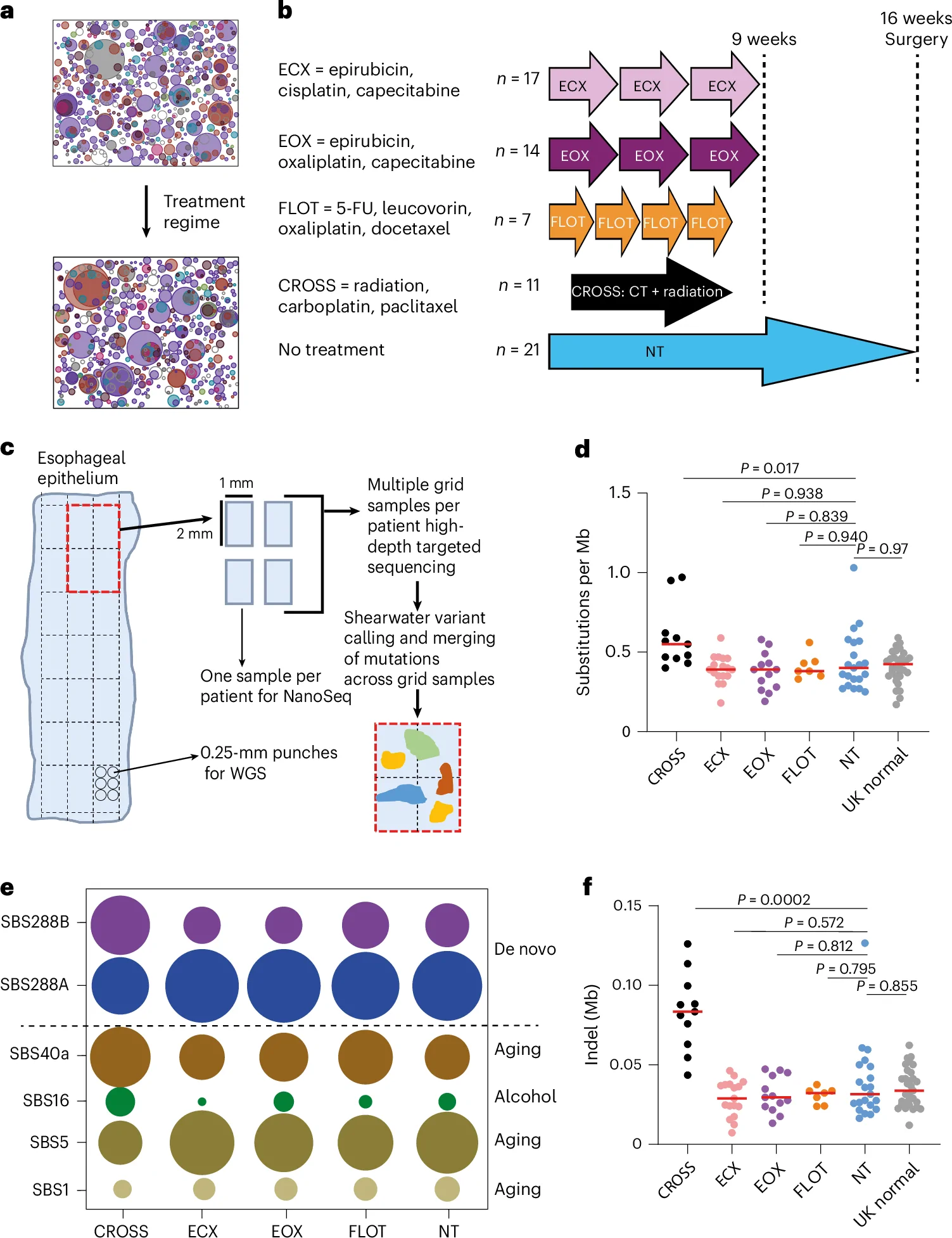
Mutational burden and signature analysis. **a**, Representation of mutant clone patchwork in normal esophagus and hypothesized changes after chemotherapy or radiotherapy. Circles of different colors represent clones carrying different mutations. **b**, Treatment schedules received by each group of patients; the details of drugs in each regime are shown in Supplementary Table 1. ECX *n* = 17 patients, EOX *n* = 14 patients, FLOT *n* = 7 patients, CROSS *n* = 11 patients, no treatment (NT) *n* = 21 patients. **c**, Sequencing protocol. Esophageal epithelium was peeled away from the submucosa and cut into a gridded array of 2-mm^2^ samples. Each 2-mm^2^ sample was sequenced at more than 250× coverage using a targeted bait panel. Mutations were called using ShearwaterML and mutations spanning adjacent grids merged. A single 2-mm^2^ sample per patient was sequenced using duplex sequencing (NanoSeq). Smaller 0.25-mm diameter punch samples from a subset of patients were also whole-genome sequenced. **d**, Substitutions per megabase (Mb) for all treatment groups. UK normal: normal esophagus from patients without cancer (data from King et al.^3^). *P* values were determined using a Kruskal–Wallis test with Dunn’s multiple-comparisons test. **e**, Average signature for each treatment group. Average number of mutations per Mb from a de novo signature analysis (Methods and Extended Data Figs. 2 and 3) or from a deconvolved COSMIC SBS mutational signature analysis (Extended Data Fig. 4) were determined for each treatment group; the size of the circle represents the proportion of mutations attributed to that signature. **f**, Burden of indels per Mb from NanoSeq for each patient. UK normal: normal esophagus from patients without cancer (data from King et al.^3^). *P* values were determined using a Kruskal–Wallis test with Dunn’s multiple-comparisons test. Patients treated with CROSS are shown in black (*n* = 11), ECX pink (*n* = 17), EOX, purple (*n* = 13), FLOT orange (*n* = 7) and NT in blue (*n* = 21). Source data

Cancer therapy may drive selective expansion of mutant clones in normal tissues. In the hematopoietic system, for example, clones mutant for the DNA repair pathway genes *CHEK2*, *PPM1D* and *TP53* emerge in patients treated with radiation, platinum compounds or *TOP2A* inhibitors^7–10^. We hypothesized that anticancer treatment may alter the selection of mutant clones in the already densely mutated normal esophagus (Fig. 1a).

There are two common types of esophageal cancer. Squamous cell carcinomas (SCCs) evolve from the epithelium of the upper and middle esophagus, whereas adenocarcinomas develop from metaplastic Barrett’s esophagus, in the distal esophagus or gastroesophageal junction^11,12^. For either cancer type, treatment involves surgery alone if the tumor is early stage or ‘neoadjuvant’ (that is, preoperative) therapy followed by surgery for locally advanced tumors. Neoadjuvant treatments include chemotherapy with the FLOT (5-fluorouracil (5-FU), leucovorin, oxaliplatin, docetaxel), EOX (epirubicin, oxaliplatin and capecitabine) or ECX (epirubicin, cisplatin, capecitabine) regimes, which combine a platinum agent (cisplatin or oxaliplatin), with a taxane (docetaxel), an anthracycline (epirubicin) or an antimetabolite (5-FU given with folinic acid or capecitabine)^13–15^ (Fig. 1b and Supplementary Table 1). Three or four cycles of chemotherapy are given before surgery. An alternative is the CROSS protocol, combining 5 weeks of radiation therapy with concurrent weekly cycles of a taxane, paclitaxel and the platinum compound, carboplatin^16^.

**Extended Data Fig. 1:**
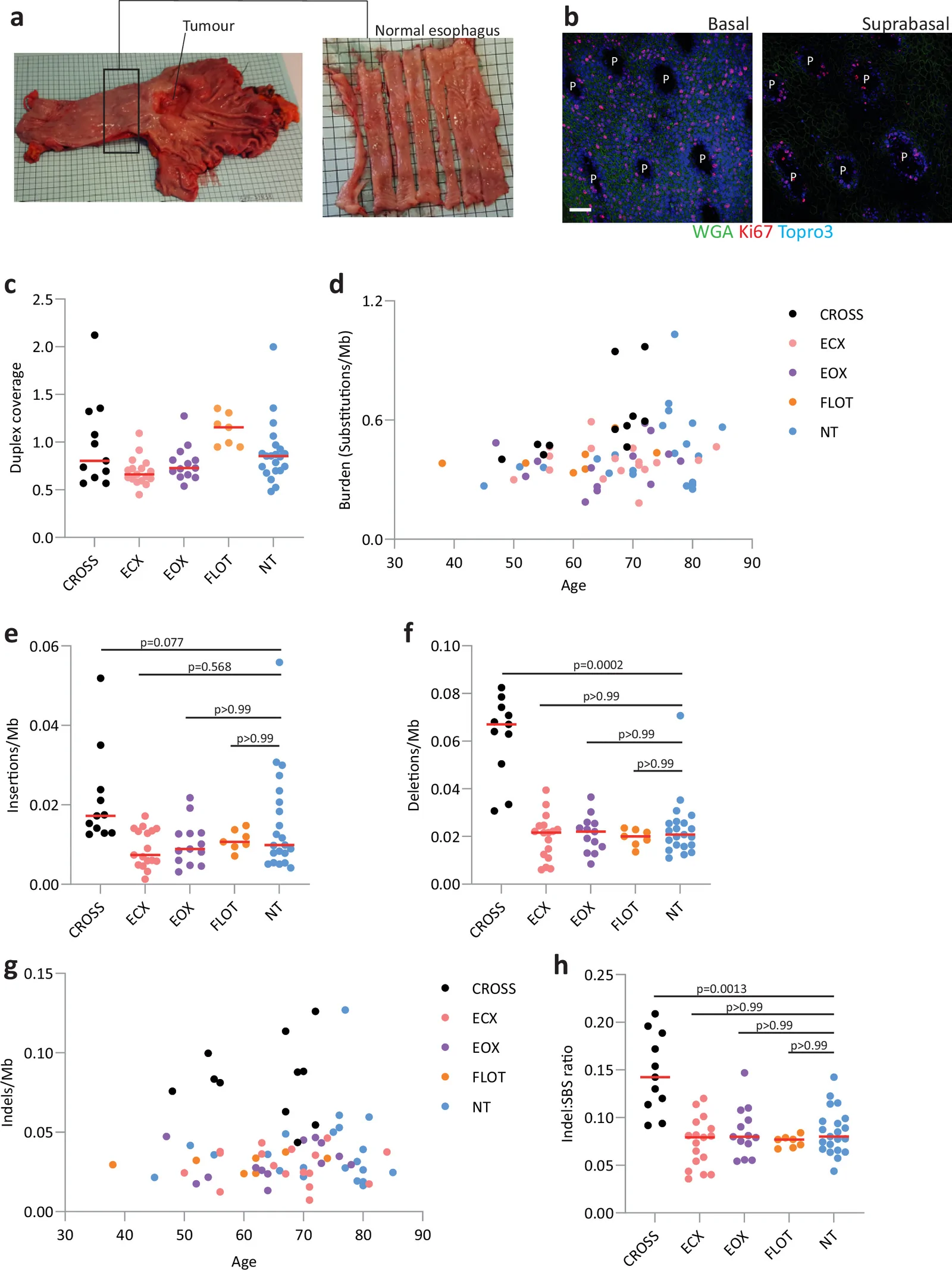
NanoSeq of normal esophageal epithelium from treated and untreated patients. **a** A typical surgical resection of the esophagus. Tissue was cut into approx. 1 cm strips before separating the epithelium from the underlying mucosa. **b** Representative confocal image of 10 peeled normal esophageal epithelium samples. The basal layer contains proliferating cells (stained with Ki67- red), in upper (Suprabasal) layers proliferation is confined to the papillae (P)^27^. Cell membranes are stained with Wheat Germ Agglutinin (WGA – green) and nuclei with Topro3 (blue). Scale bar = 40 µm. **c** Duplex coverage for all NanoSeq samples. **d** Mutational burden from Nanoseq versus age. **e-f** Insertion (**e**) and deletion (**f**) burden/Mb across treatment groups. **g** Indel burden from Nanoseq versus age. Indel burden is unrelated to age (ANOVA p = 0.48). **h** Indel:SBS ratio for each patient within all treatment groups. P-values determined by Kruskal-Wallis with Dunn’s multiple comparison test. Panels **c-h** Patients treated with CROSS represented by black points (n = 11), ECX by pink point (n = 17), EOX by purple points (n = 13), FLOT by orange points (n = 7) and no treatment (NT) by blue points (n = 21). Source data

After recovery from treatment, patients have normal esophagus removed along with the tumor (Fig. 1b and Extended Data Fig. 1a)^13,16^. The presence of normal esophagus within the surgically excised tissue gave us the opportunity to test if the mutational landscape of the normal esophageal epithelia was altered by cancer treatment using duplex, whole-genome (WGS) and targeted DNA sequencing. We found that mutational selection differed between treatment groups because of the emergence of resistant mutant clones.

## Results

Normal esophagus was collected from surgically resected material in patients who had received either no treatment (NT) (*n* = 21), or neoadjuvant ECX (*n* = 17), EOX (*n* = 14), FLOT (*n* = 7) or CROSS (*n* = 11) (Fig. 1c). The demographic and treatment details of the patients are shown in Supplementary Table 2. Normal epithelium was dissected into a gridded array of 2-mm^2^ samples, which were imaged to confirm the absence of tumor and then subjected to DNA sequencing (Extended Data Fig. 1a,b, Fig. 1b,c and Supplementary Table 2)^2,3^.

### Mutational burden and signatures in posttreatment esophagus

**Extended Data Fig. 2:**
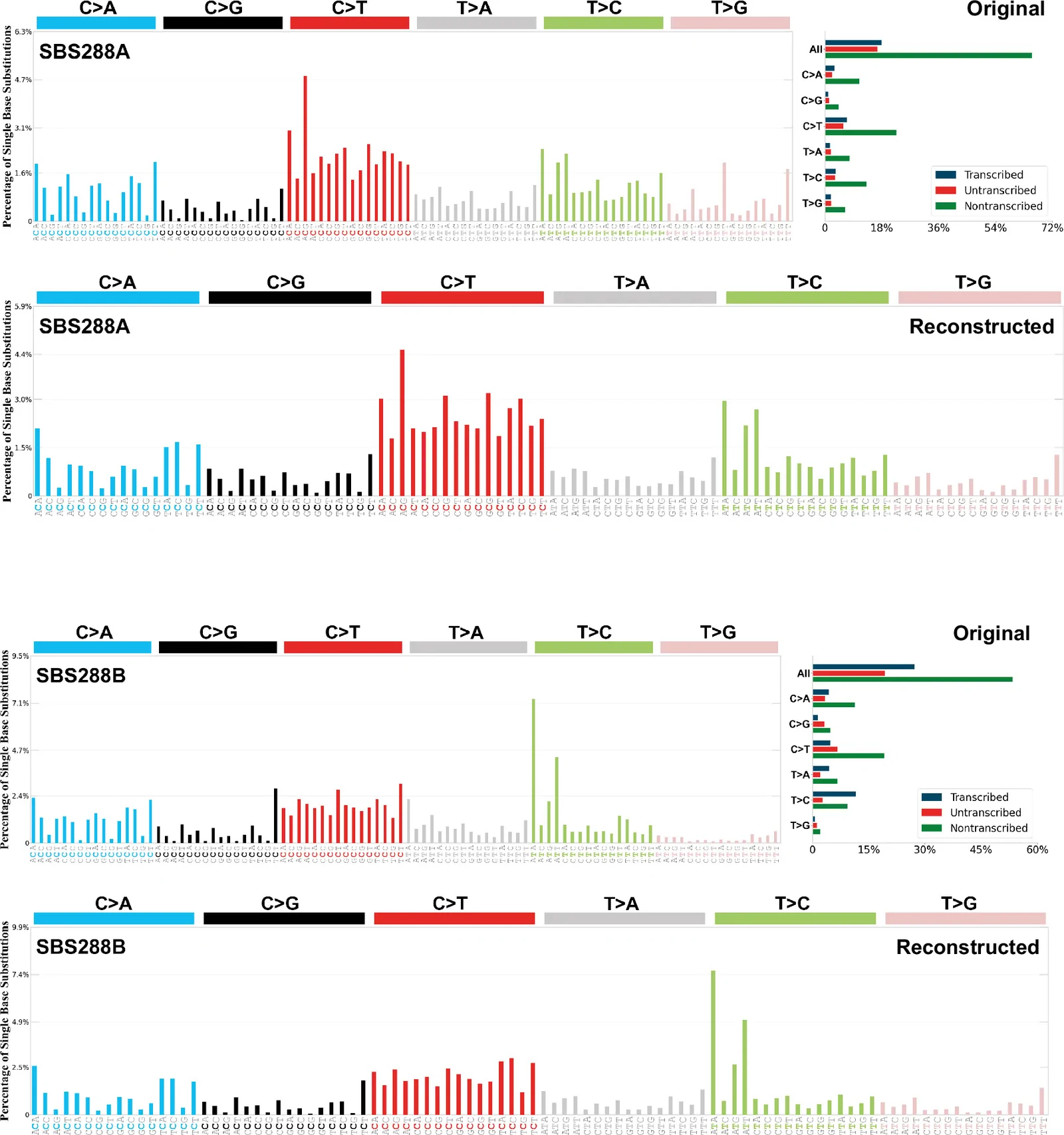
De novo signature analysis of nanoseq sequencing of all samples. Results of a de novo mutational signature analysis of all Nanoseq samples. Two de novo signatures, SBS288A and SBS288B were identified.

**Extended Data Fig. 3:**
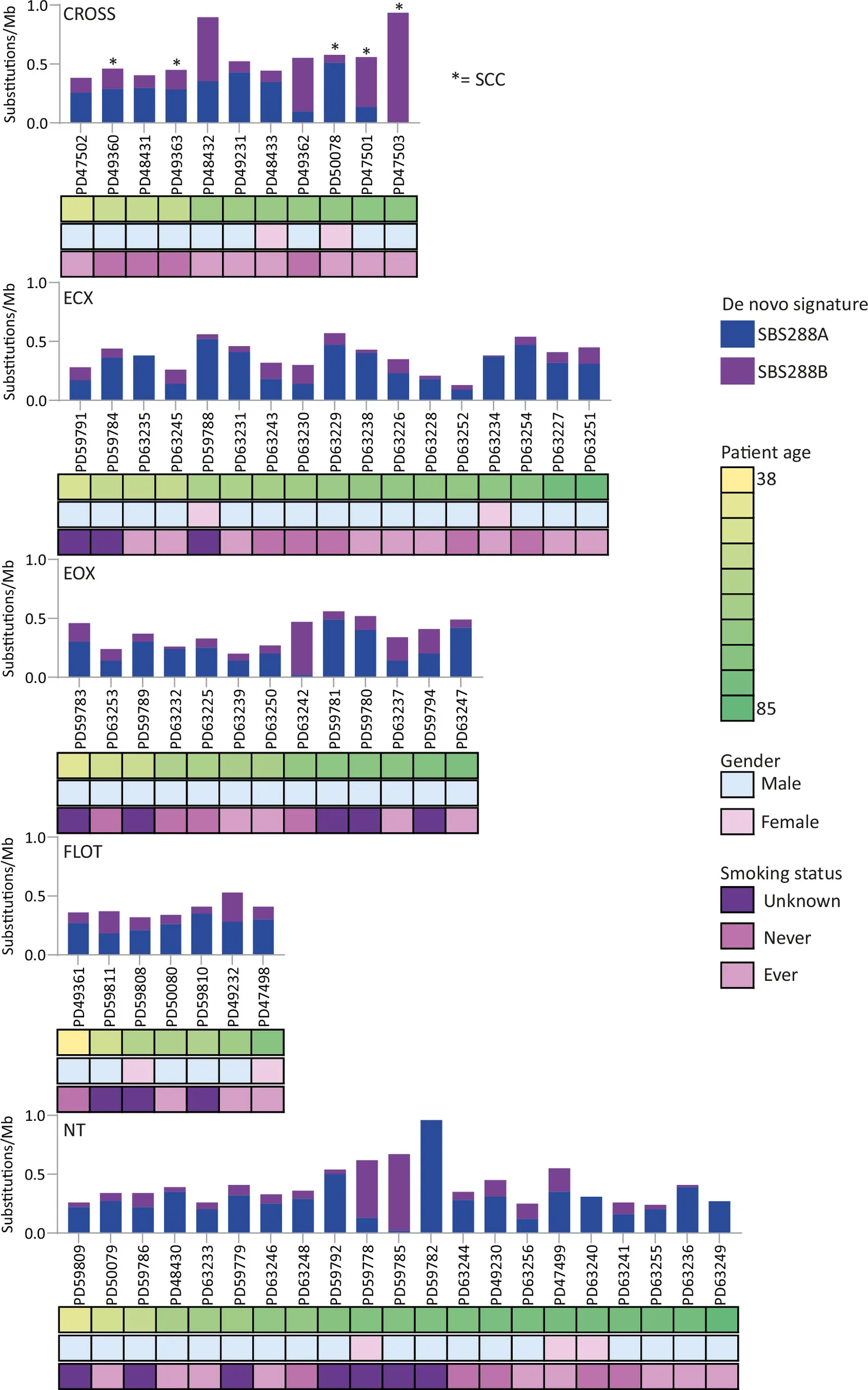
De novo analysis of nanoseq sequencing for each patient. De novo (SBS288A, SBS288B) mutations/Mb from normal esophageal epithelium from 69 patients. Age, sex and smoking status are shown below the bar plot. Samples are grouped by treatment regime. Asterisk indicates patients with squamous cell carcinoma, all other patients had adenocarcinoma. Source data

**Extended Data Fig. 4:**
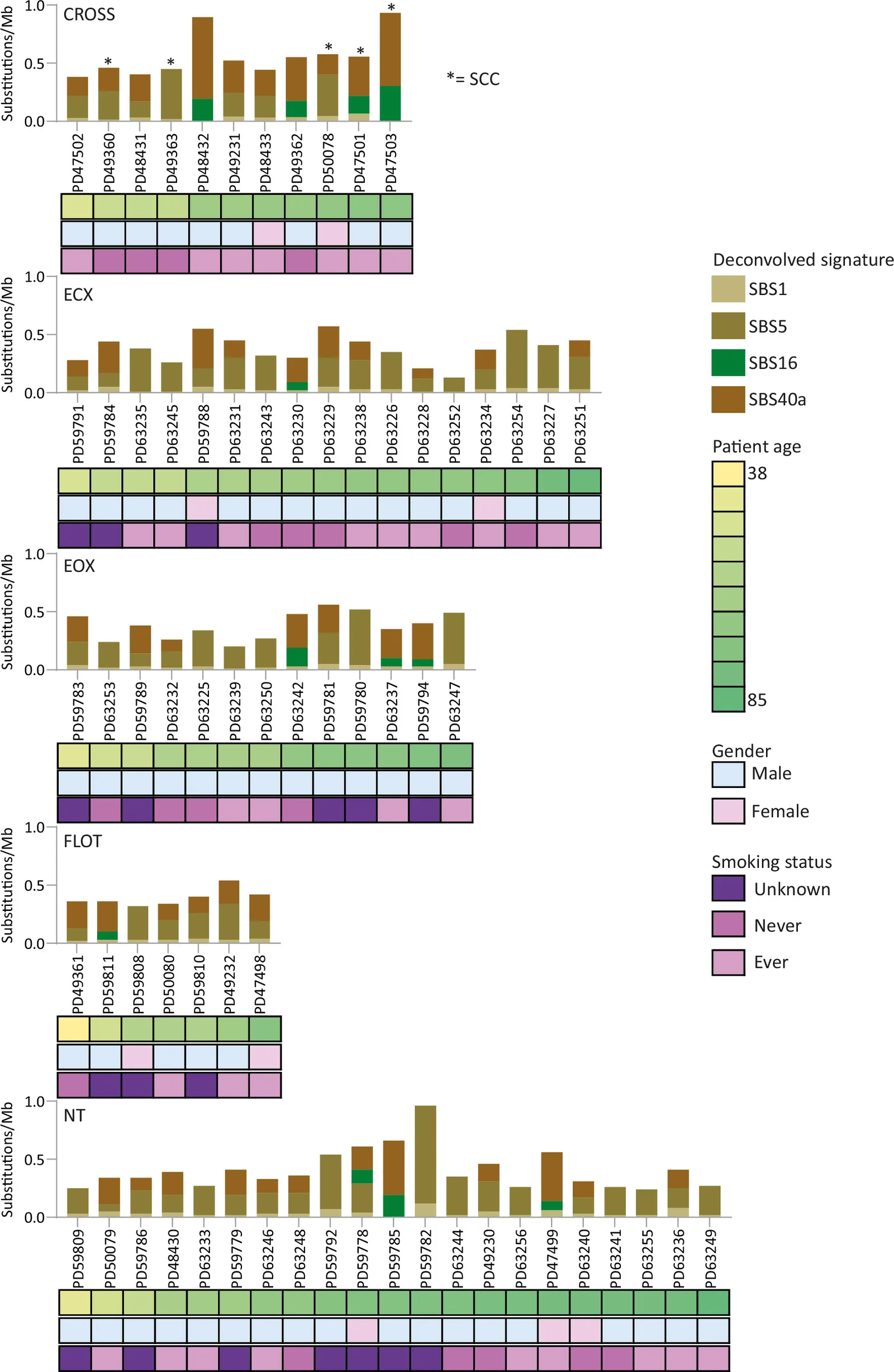
Deconvolved mutational signature analysis of nanoseq data for each patient. Deconvolved (SBS1, SBS5, SBS16 and SBS40a) mutations/Mb from normal oesophageal epithelium from 69 patients. Age, sex and smoking status are shown below the bar plot. Samples are grouped by treatment regime. Asterisk indicates patients with squamous cell carcinoma, all other patients had adenocarcinoma. Source data

We began by investigating whether neoadjuvant treatment altered the mutational burden of the normal esophagus using nanorate sequencing (NanoSeq). This is a modification of duplex sequencing that detects somatic mutations in single molecules in polyclonal samples, and determines the mutational burden and mutational signatures across 30% of the genome^17^. Importantly, for this study, NanoSeq is insensitive to differences in clonality between samples. Similar sequencing coverage was achieved across all groups (Extended Data Fig. 1c). There was no significant difference in mutational burden between the NT, EOX, ECX and FLOT treatment groups, which all had a similar burden to that reported for normal esophagus in age-matched patients without cancer^3^. However, the burden in CROSS patients was significantly higher than the other groups^2,3,17^ (Fig. 1d, Extended Data Fig. 1d and Supplementary Table 3). No association with other factors such as age, sex or smoking status was observed (Supplementary Note and Supplementary Tables 4 and 5). De novo signature analysis revealed two signatures—SBS288A and SBS288B (Fig. 1e, Extended Data Figs. 2 and 3 and Supplementary Table 3). Signature A was a combination of the ‘clock-like’ signatures SBS1 (10%), SBS5 (49%) and SBS40a (41%) (https://cancer.sanger.ac.uk/signatures/sbs/; cosine similarity 0.97). Signature B showed a combination of SBS1 (4%), SBS5 (17%) and SBS40a (54%), together with SBS16 (24%) (cosine similarity 0. 98) (Fig. 1e, Extended Data Fig. 4 and Supplementary Table 3)^18^. SBS16 is correlated with alcohol ingestion in normal esophagus and ESCC^19^. There was no significant effect of treatment in the number of Signature A mutations per Mb (*P* = 0.905, Kruskal–Wallis test) (Supplementary Note and Supplementary Table 4) or Signature B mutations per Mb (*P* = 0.1163 Kruskal–Wallis test) (Supplementary Note and Supplementary Table 4). Notably, the mutational signatures of 5-FU, oxaliplatin or carboplatin (SBS25, 31 and 35; https://cancer.sanger.ac.uk/signatures/sbs/) were absent^18,20,21^. To further investigate our mutational spectra, we ran mSigAct (Methods) using SBS1, SBS5, SBS16 and SBS40a as a baseline and tested if the addition of any of the chemotherapy-associated signatures—SBS17b, SBS25, SBS31 or SBS35—significantly improved the reconstruction^22^. Although the reconstruction was significantly improved in some patients in the treatment groups, it was also improved in patients in the NT group to a similar degree. We concluded that we cannot reliably identify treatment-specific signatures in any of the treatment groups (Supplementary Table 6).

There was a significantly higher burden of insertions and deletions (indels), specifically deletions, in CROSS patients (Fig. 1f, Extended Data Fig. 1e–h and Supplementary Tables 4 and 5) consistent with exposure to ionizing radiation^23,24^.

### Selection of mutant clones

**Extended Data Fig. 5:**
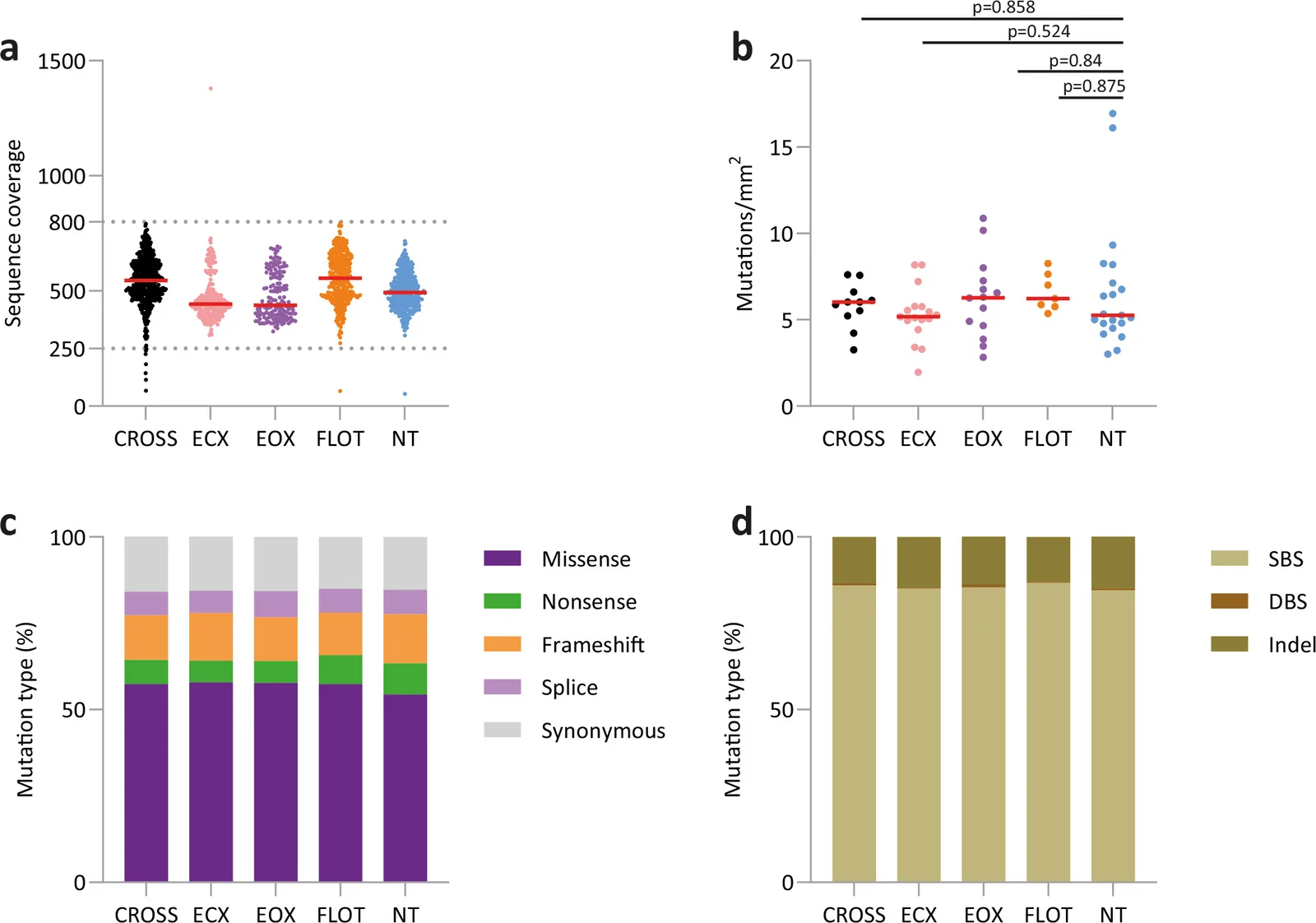
Targeted sequencing of esophageal epithelium from all groups. **a** On-target sequence coverage for each 2mm^2^ grid sample. Median is depicted by a red line. Grids with an average sequence coverage >800x or <250x coverage (depicted with dotted lines) were excluded from further analysis. CROSS n = 715, ECX n = 260, EOX n = 163, FLOT n = 401, No treatment (NT) n = 456. **b** Average number of mutations per mm^2^. Each point represents a patient (CROSS n = 11, ECX n = 17, EOX n = 14, FLOT n = 7, NT n = 21). P-values determined by Kruskal-Wallis with Dunn’s multiple comparison test. **c** Proportion of the types of mutations in each treatment group. Percentage of mutation types are similar across treatment groups (p = 0.00077 Kendall concordance test) **d** Proportion of single base (SBS), double base (DBS) and insertion/ deletions (indel) in each treatment group from targeted sequencing. Percentages are similar across treatment groups (p = 0.007, Kendall’s concordance test) For **c** and **d** CROSS n = 7984, ECX n = 2701, EOX n = 1831, FLOT n = 5028, NT n = 6148 Source data

Next, we examined somatic mutant clones using targeted sequencing of 1,995 2-mm^2^ samples across the five groups for 324 cancer-associated genes with median on-target coverage of ca. 500× (Extended Data Fig. 5a and Supplementary Table 7). A total of 23,692 independent mutations (Methods and Supplementary Table 8) were identified. After adjusting for the area sampled in each patient, the density of mutations, types of mutations, and the proportions of SBS and double-base substitutions, were similar in all groups (Extended Data Fig. 5b–d and Supplementary Table 8).

The absence of chemotherapy-specific SBS mutational signatures, and similar density of mutants across all groups, argues that most of the mutant clones we detected were likely to have existed before therapy rather than being new mutants generated by treatment. This in turn suggests that differences between groups reflect alterations in the selection of mutants under different treatments.

**Fig. 2:**
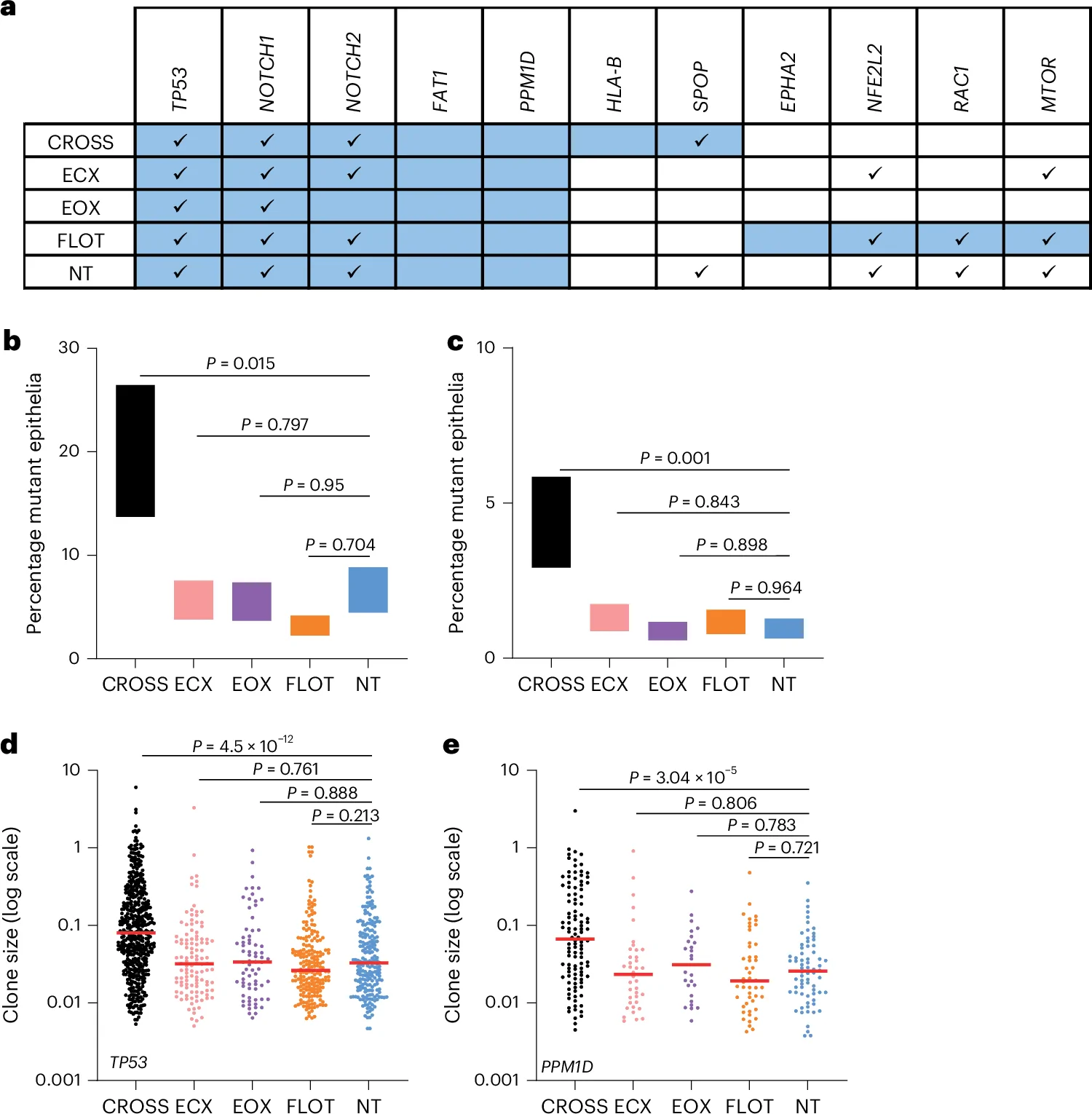
Chemoradiation expands *TP53* and *PPM1D* mutant clones. **a**, Mutant selection across groups. The blue shading indicates genes under positive selection according to the *d*N-to-*d*Scv ratio (Methods). Only genes selected in all or a single treatment group and with *q* < 0.01 are shown. (The full list of genes under selection is shown in Supplementary Tables 9–13 and Methods.) The presence of ticks (✓) indicates significant 1D clustering of missense mutants according to NMC. Details of the numbers of clusters and amino acid location are shown in a GitHub repository^80^. **b**,**c**, Average percentage mutant epithelia for *TP53* (**b**) and *PPM1D* (**c**) across all patients within a treatment group. The upper and lower bounds allow for uncertainty in copy number and nested mutations. The lower bound shows the result of biallelic mutation of the gene in all mutant cells with copy neutral loss of heterozygosity or compound heterozygous mutations. The upper limit corresponds to one mutant allele per cell at a diploid locus. CROSS *n* = 11, ECX *n* = 17, EOX *n* = 14, FLOT *n* = 7, NT *n* = 21 patients. *P* values were calculated by comparing the lower bound values from each patient using a Kruskal–Wallis test with Dunn’s multiple-comparisons test. **d**, *TP53* clone size (summed VAF) in each of the treatment groups. The median in each group is shown with a red line. CROSS *n* = 468, ECX *n* = 114, EOX *n* = 72, FLOT *n* = 204, NT *n* = 209 clones. *P* values were determined using a Kruskal–Wallis test with Dunn’s multiple-comparisons test. **e**, *PPM1D* clone size (summed VAF) in each of the treatment groups. The median in each group is shown with a red line. CROSS *n* = 110, ECX *n* = 38, EOX *n* = 26, FLOT *n* = 48, NT *n* = 70. *P* values were determined using a Kruskal–Wallis test with Dunn’s multiple-comparisons test. Source data

To examine if treatment altered mutant selection, we first calculated the proportion of nonsynonymous mutations relative to synonymous mutations (*d*N-to-*d*S ratio), adjusted for the mutational spectrum and other factors, for each mutant gene in each treatment group (Fig. 2a and Supplementary Tables 9–13)^2,25^. A statistically significant increase in *d*N-to-*d*S ratio above 1 indicates that a mutant gene is under positive selection, with protein-altering mutations driving clonal expansion^26,27^. We required eight or more nonsynonymous mutations if there were zero synonymous mutations for a mutant gene to be considered under positive selection (Methods). We did not observe a correlation between the number of genes under positive selection and the number of mutations in each group (Pearson correlation 0.45, two-tailed *P* = 0.44), arguing that the differences in selection between groups are not due to variation in mutation numbers. Most patients had esophageal adenocarcinoma (EAC); only five had SCC and all of these were in the CROSS group. However, neither within the CROSS group nor over all groups was there any significant variation in any parameter with tumor type (Supplementary Note and Supplementary Tables 4 and 5).

A limitation of *d*N-to-*d*S analysis across a whole gene is that it does not reveal clustering of missense mutants. Therefore, we performed one-dimensional (1D) nonrandom mutational clustering (NMC) analysis of missense mutants in positively selected mutant genes (Fig. 2a, Supplementary Note and Supplementary Table 14)^28^. This was augmented by three-dimensional (3D) clustering analysis for proteins where experimentally determined structures deposited in the Protein Data Bank, including apo and ligand-bound conformation, were available. Below we consider mutant genes that were selected in all groups, followed by mutant genes selected in CROSS (*HLA-B* and *SPOP*) and FLOT (*NFE2L2*, *RAC1* and *MTOR*) patients.

### Expansion of *TP53* and *PPM1D* mutant clones in CROSS patients

**Extended Data Fig. 6:**
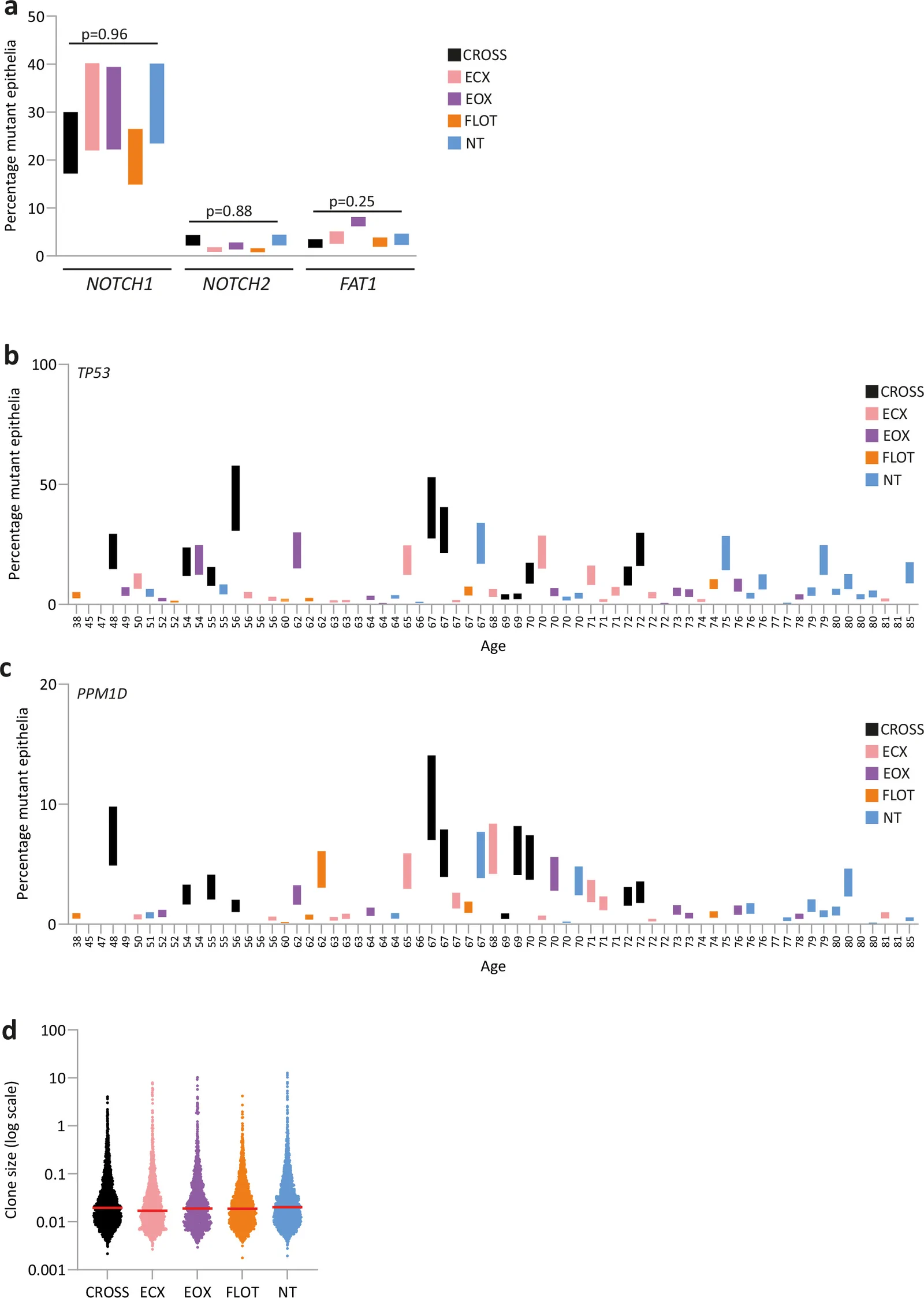
*TP53* and *PPM1D* mutant clones cover a greater proportion of the epithelium in CROSS patients. **a** Average percentage mutant epithelia of genes under positive selection using *d*N/*d*Scv (q < 0.01) in all treatment groups. Average calculated from CROSS n = 11, ECX n = 16, EOX n = 14, FLOT n = 7, NT n = 21 patients. P-values calculated by comparing lower bound values from each patient using Kruskal-Wallis test with Benjamini Hochberg multiple test correction (Extended Data Table 15). **b-c** Percentage epithelia carrying mutations in *TP53* (**b**) and *PPM1D* (**c**) in each patient plotted against age. **a-c** In all cases upper and lower bounds allow for uncertainty in copy number and nested mutations. Lower bound shows the result of biallelic mutation of the gene in all mutant cells with copy neutral loss of heterozygosity or compound heterozygous mutations. Upper bound corresponds to one mutant allele per cell at a diploid locus. **d** Non-synonymous clone size distribution (VAF) across all sequenced genes excluding TP53 and PPM1D. Red bar indicates median CROSS n = 6139, ECX n = 2112, EOX n = 1439, FLOT n = 4003, NT n = 4902. P value (two-tailed ANOVA) = 1.82e-9 however effect size is practically insignificant (eta-squared=0.0025). In all panels data from patients receiving CROSS shown as black bars/points, ECX as pink bars/points, EOX as purple bars/ points, FLOT by orange bars/ points and no treatment (NT) as blue bars/ points. Source data

The most prevalent mutant genes (*TP53*, *PPM1D*, *NOTCH1*, *NOTCH2* and *FAT1*) were positively selected in all groups (Fig. 2a and Supplementary Tables 9–13). These mutants are also selected in normal epithelium from donors without cancer^1–3^. The proportion of epithelium carrying nonsynonymous mutants of *FAT1*, *NOTCH1* and *NOTCH2* was similar in all groups (Extended Data Fig. 6a and Supplementary Table 15). However, the proportion of tissue mutant for *TP53* and *PPM1D* in CROSS patients was double that of the other groups (Fig. 2b,c and Extended Data Fig. 6b,c). Consistent with this, the estimated size of *TP53* and *PPM1D* mutant clones was substantially larger in CROSS patients compared with the other groups (Fig. 2d,e). In contrast, the median size of clones carrying nonsynonymous mutations in all other genes showed minimal differences across groups (Extended Data Fig. 6d). These changes argue that *TP53* and *PPM1D* mutants gain an additional selective advantage during chemoradiation but not other treatments.

**Fig. 3:**
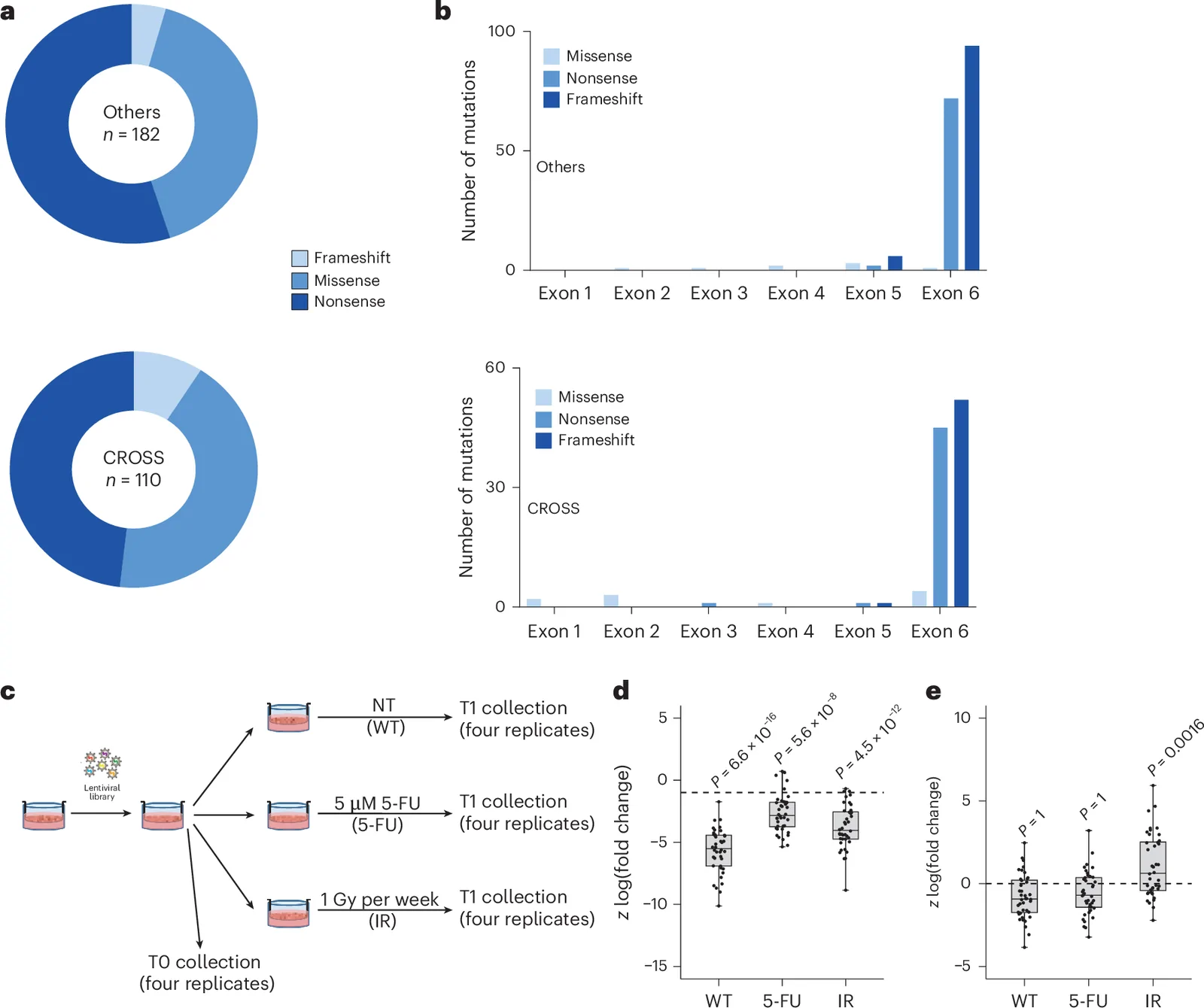
*TP53* and *PPM1D* mutations. **a**, Proportion of *PPM1D* mutations showing frameshift, missense or nonsense mutations. Others include mutations from ECX, EOX, FLOT and NT patients. **b**, Location of *PPM1D* mutation according to exon. Others include mutations from ECX, EOX, FLOT and NT patients. CROSS *n* = 110, others *n* = 182. **c**, CRISPR–Cas9 protocol. Epithelioid cultures were established from the esophageal epithelium of transgenic mice expressing Cas9. Cultures were transduced with a gRNA lentiviral library. Time point 0 (T0) DNA was collected 4 days later. Cultures were treated with either 5-FU or irradiation or untreated; DNA was collected after 3 weeks of treatment (T1) and gRNA was sequenced. **d**,**e**, log fold change of gRNA targeting exon 1 (**d**) or exon 6 (**e**) of *Ppm1d* at T1 relative to T0. The dots indicate gRNAs; all ten guide RNAs from each of the four biological replicates are shown. The central line shows the median; the box indicates the quartiles; and the bars indicate the minimum and maximum. Thresholds of depleted (**d**) and enriched (**e**) guides are shown by dotted lines and inferred from control guides (Methods). *P* values represent the significance of guides being depleted (**d**) or enriched (**e**) via a one-sided *t*-test with Benjamini–Hochberg multiple test correction. Source data

As in individuals without cancer, over 90% of *PPM1D* mutations in all groups were nonsense or frameshift mutations within exon 6 (Fig. 3a,b)^1,3^. Such mutants generate a stabilized form of the protein that retains phosphatase activity, inhibiting TP53 function, activating MTORC1 and conferring resistance to chemotherapy and ionizing radiation in mutant hematopoietic stem cells, leading to clonal hematopoiesis^8,9,29–32^. To test if exon 6 *PPM1D* mutants are preferentially selected in irradiated esophageal epithelium, we performed CRISPR–Cas9 editing of *Ppm1d* in primary murine esophageal epithelioid cultures (Fig. 3c and Supplementary Note)^33–35^. Deletion of *Ppm1d* by guide RNAs (gRNAs) targeting exon 1 reduced clonal fitness whereas selection of gRNAs targeting exon 6 was increased in irradiated epithelioids compared with those treated with 5-FU or untreated cultures (Fig. 3d,e). We conclude that radiation selectively expands clones carrying truncating, gain of function (GOF) *PPM1D* mutants in esophageal epithelium, although this experiment does not test the carboplatin, paclitaxel and radiation combination given to CROSS patients.

**Fig. 4:**
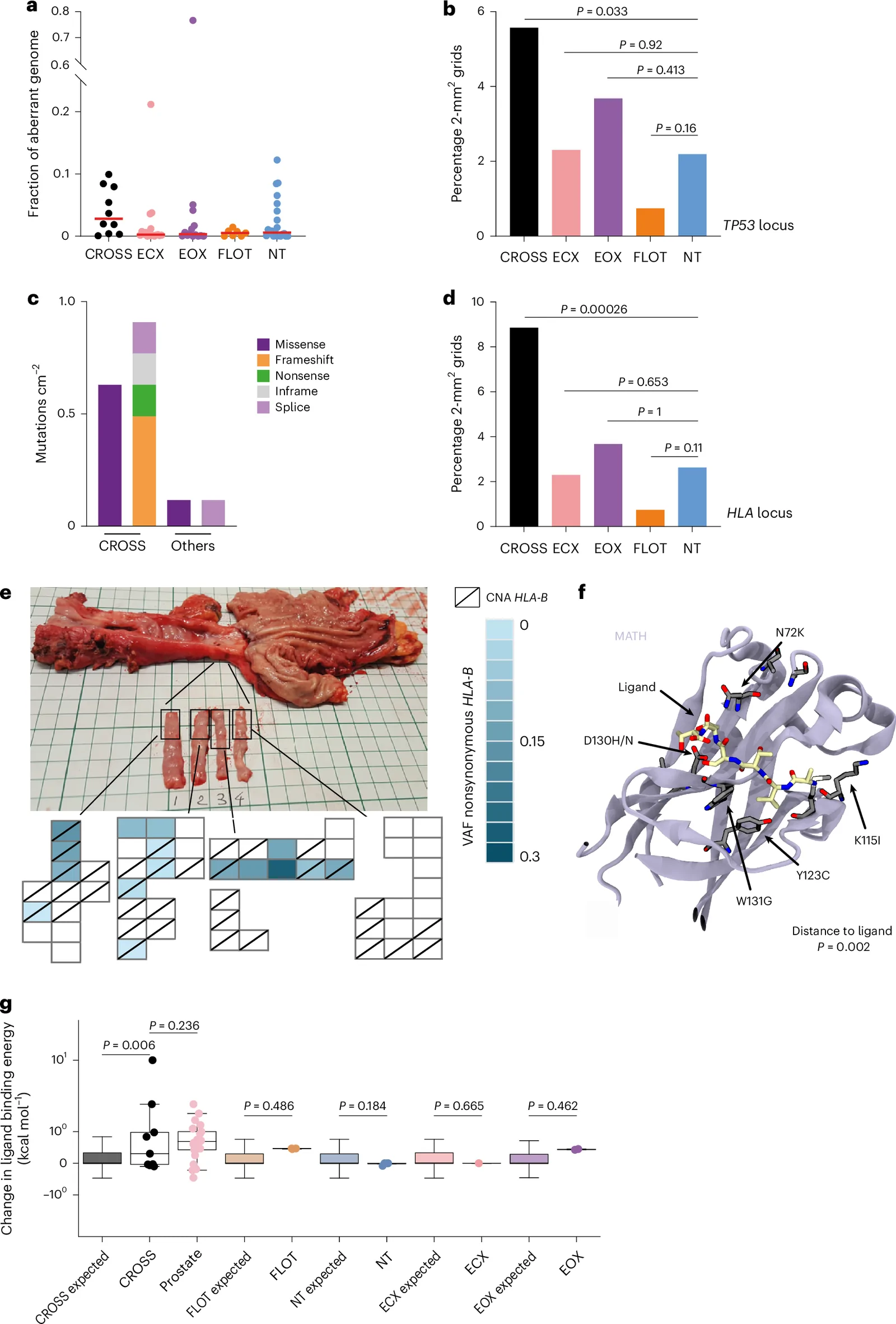
Selected mutations and CNAs in CROSS patients. **a**, Average fraction of the genome carrying CNAs. Each dot represents a patient. The median is represented by a red line. CROSS *n* = 10, ECX *n* = 17, EOX *n* = 14, FLOT *n* = 7, NT *n* = 21 patients. *P* = 0.069, Kruskal–Wallis test. **b**, Percentage of 2-mm^2^ samples carrying copy number events affecting the *TP53* locus (chr17:7571731–7580757) in each treatment group; *P*values from a two-tailed Fisher’s exact test with Benjamini–Hochberg multiple test correction. **c**, Selection of HLA mutants. Types of HLA mutations cm^−^^2^ in CROSS patients and all other treatment groups (others) (*P* = 5.3 × 10^−7^ Freeman–Halton–Fisher test). **d**, Percentage of 2-mm^2^ grids carrying copy number events covering the HLA locus (chr6:29691116–31373046); *P* values from a two-tailed Fisher’s exact test with Benjamini–Hochberg multiple test correction. **e**, Overview of all samples from patient PD47501. Each square is a 2-mm^2^ grid sample. The spatial location of adjacent 2-mm^2^ samples is shown; disconnected groups of samples were from the same area but were not physically joined. Copy number events at 6p are denoted by diagonal strikethrough. Nonsynonymous mutations are indicated by the blue shading; depth of color indicates the VAF. Eleven independent *HLA-B* mutant clones were detected in this patient. **f**, Structure of SPOP. The MATH domain with ligand bound is shown, with mutations in CROSS patients highlighted in black. **g**, Change in ligand binding energy (ΔΔG binding) for mutations observed in each condition compared to an expected distribution. Also included are known LOF mutations in SPOP observed in prostate cancer. The *P* value represents a two-tailed Monte Carlo test applied to mean ΔΔG binding for observed mutations versus the computed neutral distribution. The box plot central line shows the median; the box indicates the quartiles. Whiskers extend to the furthest data point within 1.5 times the interquartile range (IQR) of each box edge. Source data

**Extended Data Fig. 7:**
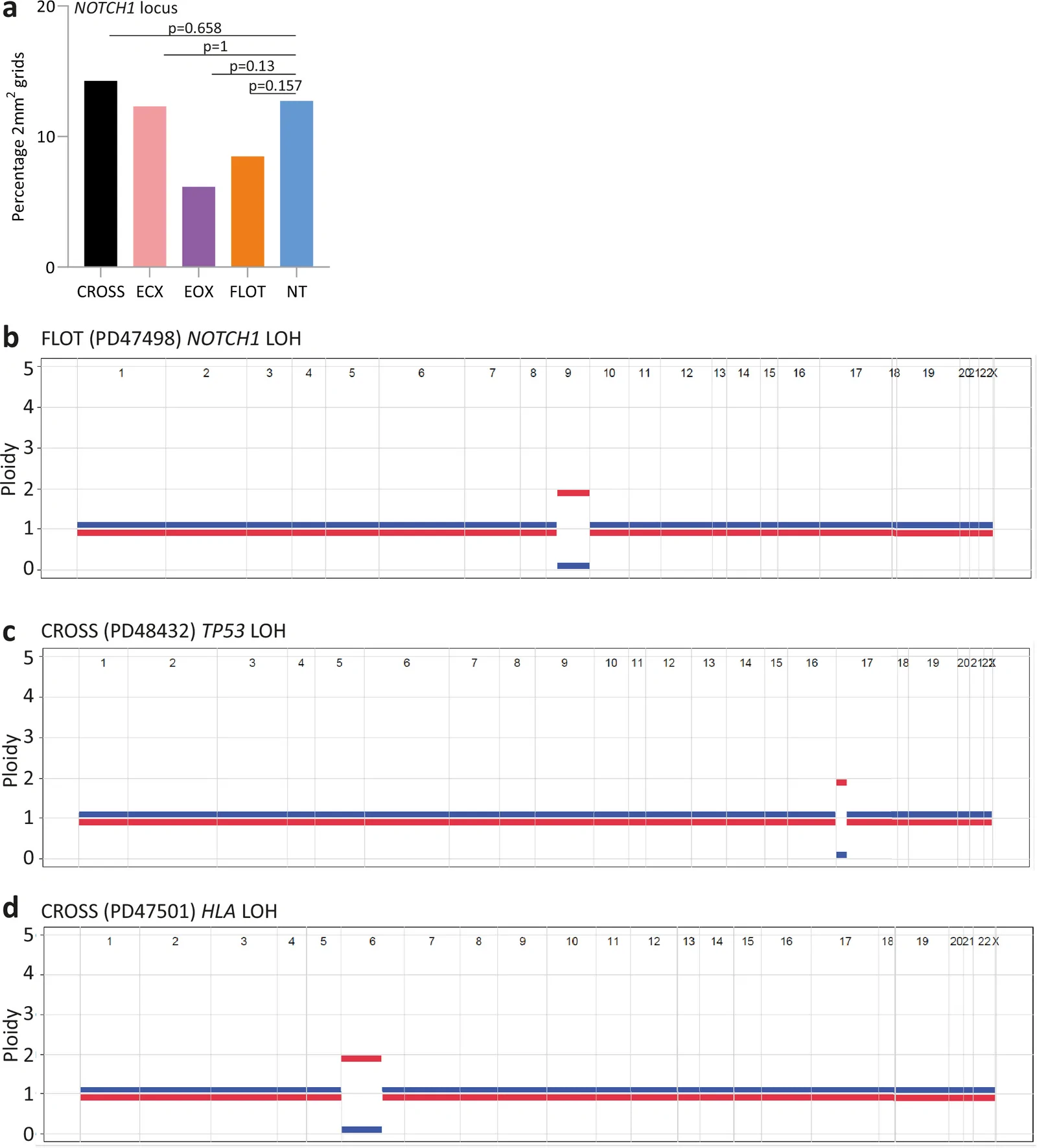
Copy number alterations detected by targeted sequencing. **a** Percentage of 2mm^2^ samples carrying copy number events covering the *NOTCH1* locus (chr9: 139388885- 139407300). CROSS n = 610, ECX n = 260, EOX n = 163, FLOT n = 401, NT n = 456. P-values derived from two-tailed Fisher’s exact test with Benjamini-Hochberg multiple test correction. Examples of altered copy number at the *NOTCH1* locus (**b**), *TP53* locus (**c**) and *HLA* locus (**d**). Panels b-d depicted chromosome lengths are linearly scaled according to the number of usable heterozygous SNPs. Source data

The increased indel burden and expanded *TP53* mutant clones within the CROSS group led us to speculate that copy number alterations (CNAs) may be occurring in mutant clones in these patients because of loss of functional *TP53*. Therefore, we performed copy number calling from targeted sequencing in the 2-mm^2^ samples (Supplementary Table 16). This revealed an increased fraction of the genome with CNA within the CROSS group, although this did not reach statistical significance (*P* = 0.069, two-tailed Kruskal–Wallis test) (Fig. 4a). Patients in all groups had CNA events involving the *NOTCH1* locus on chromosome 9q (Extended Data Fig. 7a,b). These changes are also seen in patients without cancer and are common as biallelic disruption of *NOTCH1* increases clonal fitness^1–3,36^. However, over 5% of CROSS samples carried a CNA event on chromosome 17p covering the *TP53* locus, a significantly higher proportion than for other treatment groups (Fig. 4b and Extended Data Fig. 7c).

**Fig. 5:**
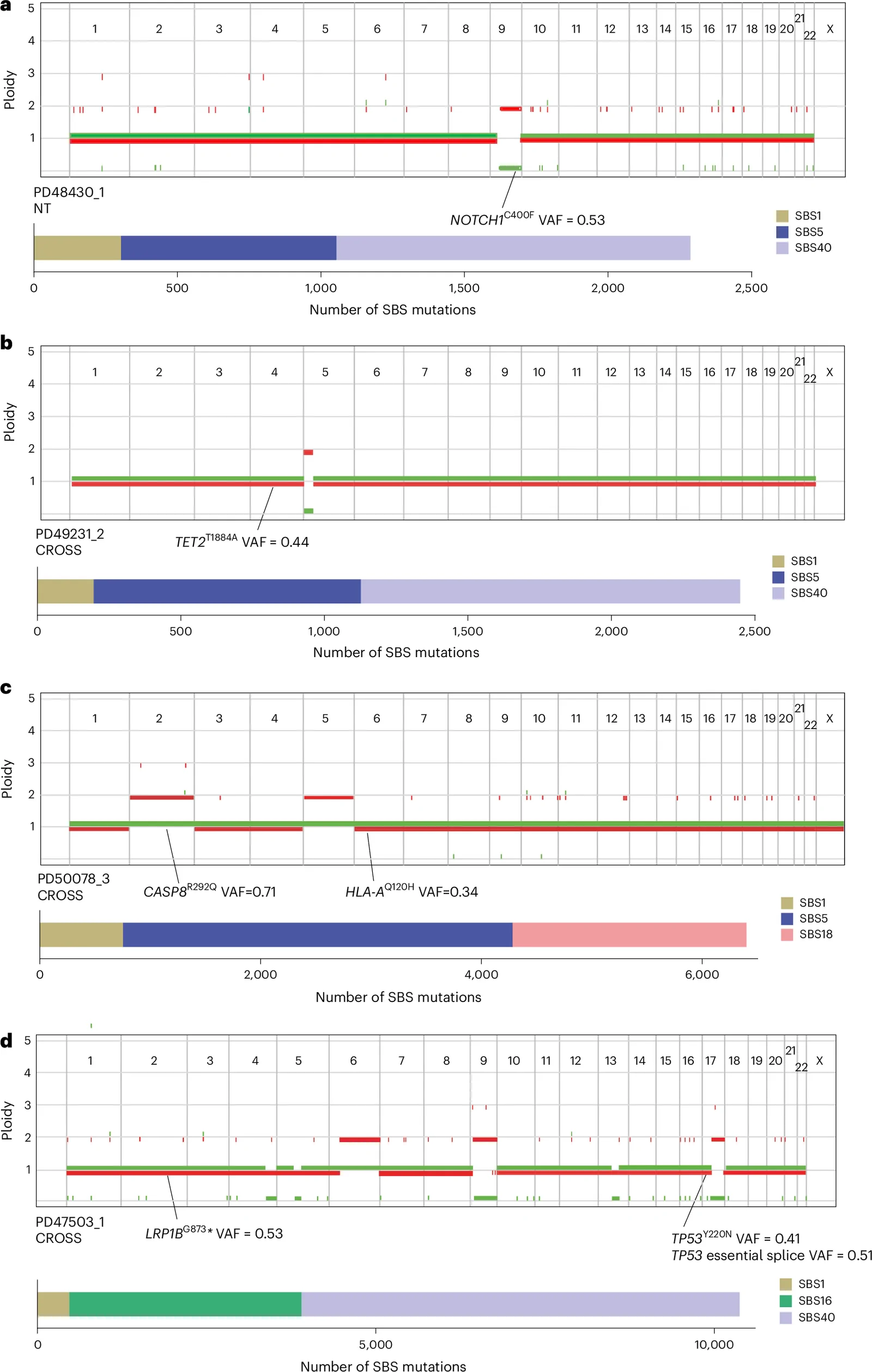
WGS reveals CNAs in CROSS patients. **a**–**d**, Examples of allele-specific copy number analysis of tumors (ASCAT) profiles showing copy number changes in typical clones from NT (**a**) and CROSS groups (**b**–**d**), ranging from small focal LOH events (**b**) to whole-chromosome gains (**c**) and, in occasional cases, multiple complex events (**d**). Decomposed signatures are shown below the ASCAT profile along with potential driver mutations and associated VAF. Depicted chromosome lengths are linearly scaled according to the number of usable heterozygous single-nucleotide polymorphisms (SNPs). Source data

To augment the CNA analysis, we performed WGS of 37 clonal 0.05-mm^2^ samples from 12 patients in the NT, FLOT and CROSS groups, who had a mutation with variant allele frequency (VAF) > 0.35 (Supplementary Tables 17 and 18). Mutational signature analysis confirmed the absence of chemotherapy signatures and a nonsignificant trend toward increased indels in the CROSS group consistent with the NanoSeq data (Supplementary Table 18). Loss of heterozygosity (LOH) of the *NOTCH1* gene was observed as expected^2,3,36^. However, CNA in the CROSS group ranged from LOH of driver genes, including *TP53* and *PPM1D*, to events involving entire chromosomes (Fig. 5a–c and Supplementary Table 18). Three of 27 CROSS clones had biallelic *TP53* disruption, which was not seen in the four FLOT or six NT samples (Fig. 5d and Supplementary Table 18). Biallelic *TP53* disruption occurs early in esophageal squamous carcinogenesis and is associated with CNA^37–39^.

### *HLA-B* and *SPOP* mutants are selected only in CROSS patients

The *d*N-to-*d*S ratio and NMC analyses revealed mutant genes that were positively selected in specific treatment group(s) (Fig. 2a). In CROSS patients these included *HLA-B* (Fig. 2a and Supplementary Tables 9–13) with five of 11 patients carrying at least one nonsynonymous mutation. Thirteen of 22 (59%, equating to 0.91 mutations cm^−^^2^) *HLA-B* nonsynonymous mutations detected were frameshift, splice or nonsense mutations, which are likely to result in no functional protein (Fig. 4c). This contrasts with 0.12 predicted *HLA-B* protein loss mutations cm^−^^2^ across all other treatment groups (Fig. 4c). The *HLA* locus on chromosome 6p also carried CNA events in 8.7% of the 2-mm^2^ samples from CROSS patients. (Fig. 4d,e and Extended Data Fig. 7d). Such disruption of *HLA* genes drives clonal expansion in cancer and autoimmune anemia, in which selection is driven by T cell immunity^40,41^.

**Extended Data Fig. 8:**
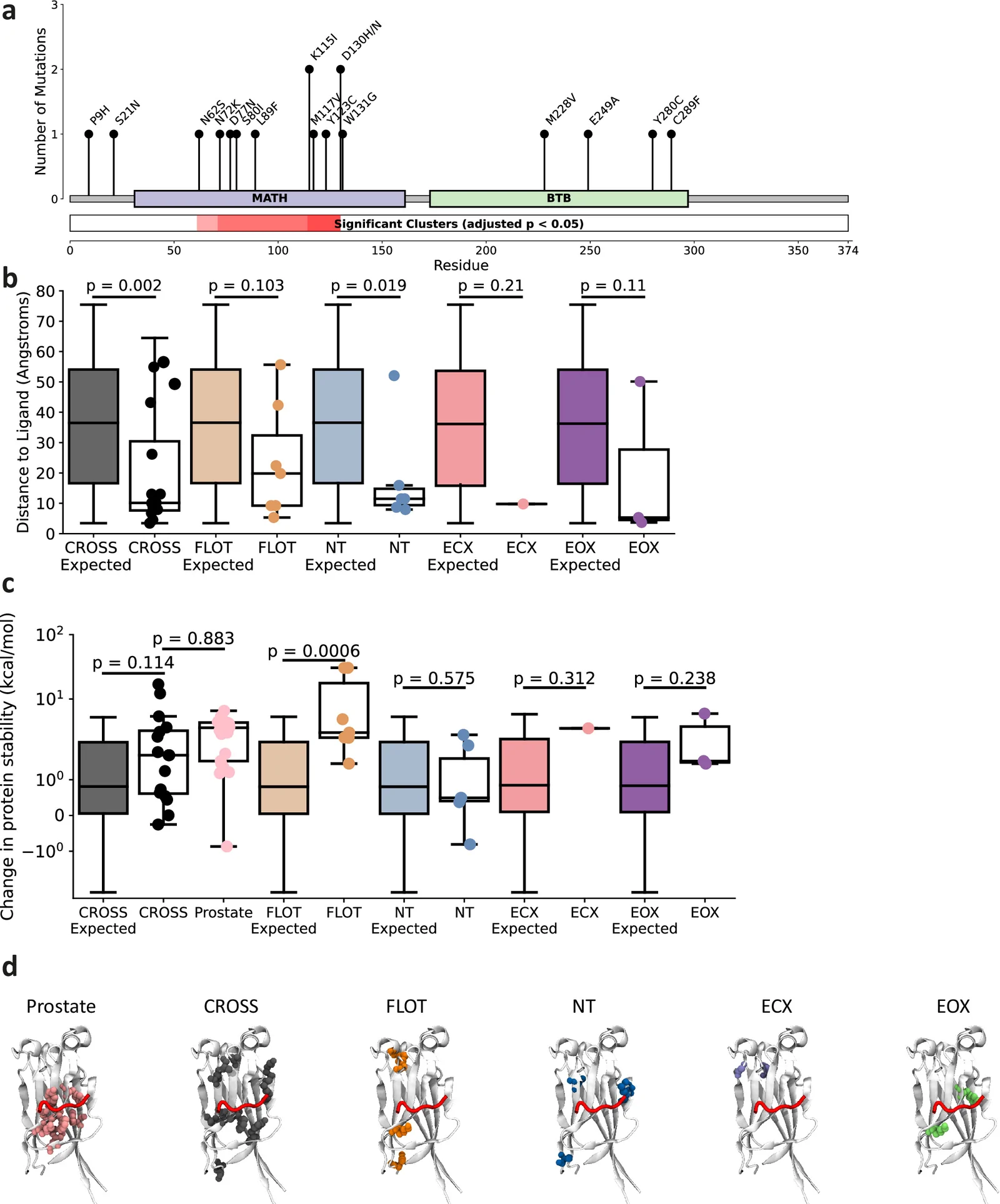
SPOP mutations in CROSS patients. **a** Lollipop plot of mutations in SPOP in observed in patients exposed to CROSS. 1D clusters are calculated using NMC (Methods). **b** Distance from mutations to ligand in SPOP compared to an expected distribution. P value represents Monte Carlo test applied to median distance from ligand vs an expected distribution given the mutational signature. **c** Change in SPOP protein stability compared to an expected distribution. P value represents two-tailed Monte Carlo test applied to observed change in protein stability versus expected distribution given the mutational signatures within each treatment group. **d** 3D structure of SPOP showing mutations in all treatment groups and those observed in prostate cancer. Box plot central line shows median and box indicates quartiles. Whiskers extend to furthest data point within 1.5 x inter quartile range of each box edge.

Nonsynonymous mutants of *SPOP*, encoding an adapter protein involved in the DNA damage response and cell death were also selected only in CROSS patients^40,41^. Eighteen of the 21 *SPOP* mutants in the CROSS group were missense and were significantly clustered in the 1D analysis (Extended Data Fig. 8a). *SPOP* mutants may either be loss of function (LOF), reported to confer resistance to taxane chemotherapy and activate MTOR signaling in prostate cancer^42–44^, or GOF as seen in endometrial cancer^45,46^. We calculated the 3D distance of mutations to the ligand binding site and compared this to an expected distribution generated through Monte Carlo sampling based on the mutational context for each treatment group. The mutations in the CROSS and NT groups were significantly closer to the ligand than the expected distribution within each treatment group (Fig. 4f and Extended Data Fig. 8b). However, in the CROSS group, mutations that increased the binding energy of SPOP to its ligand were significantly enriched compared with the neutral distribution, suggesting that they had a functional impact (Fig. 4g). Multiple mechanisms can be responsible for LOF aside from changes in ligand binding energies; these include protein abundance and folding stability. We investigated the predicted stability of SPOP proteins carrying mutations seen in CROSS patients and found that no strong effect was observed in the folding contribution. Our analysis finds that ΔΔG is the primary observed difference between CROSS and NT groups (Extended Data Fig. 8c). *SPOP* mutations in CROSS patients showed similar 1D positional enrichment, 3D proximity to interface and ligand binding energy to the LOF mutations seen in prostate cancer (Extended Data Fig. 8d and Fig. 4g)^43,47–49^. We hypothesize that LOF missense *SPOP* mutants may be quantitatively selected by chemoradiation.

### Mutant selection enhanced by FLOT

Mutant *NFE2L2*, *RAC1* and *MTOR* showed 1D clustering in multiple groups but were only under positive selection in the FLOT group. All three mutant genes are potentially druggable, so we investigated them in more detail^50–52^.

**Extended Data Fig. 9:**
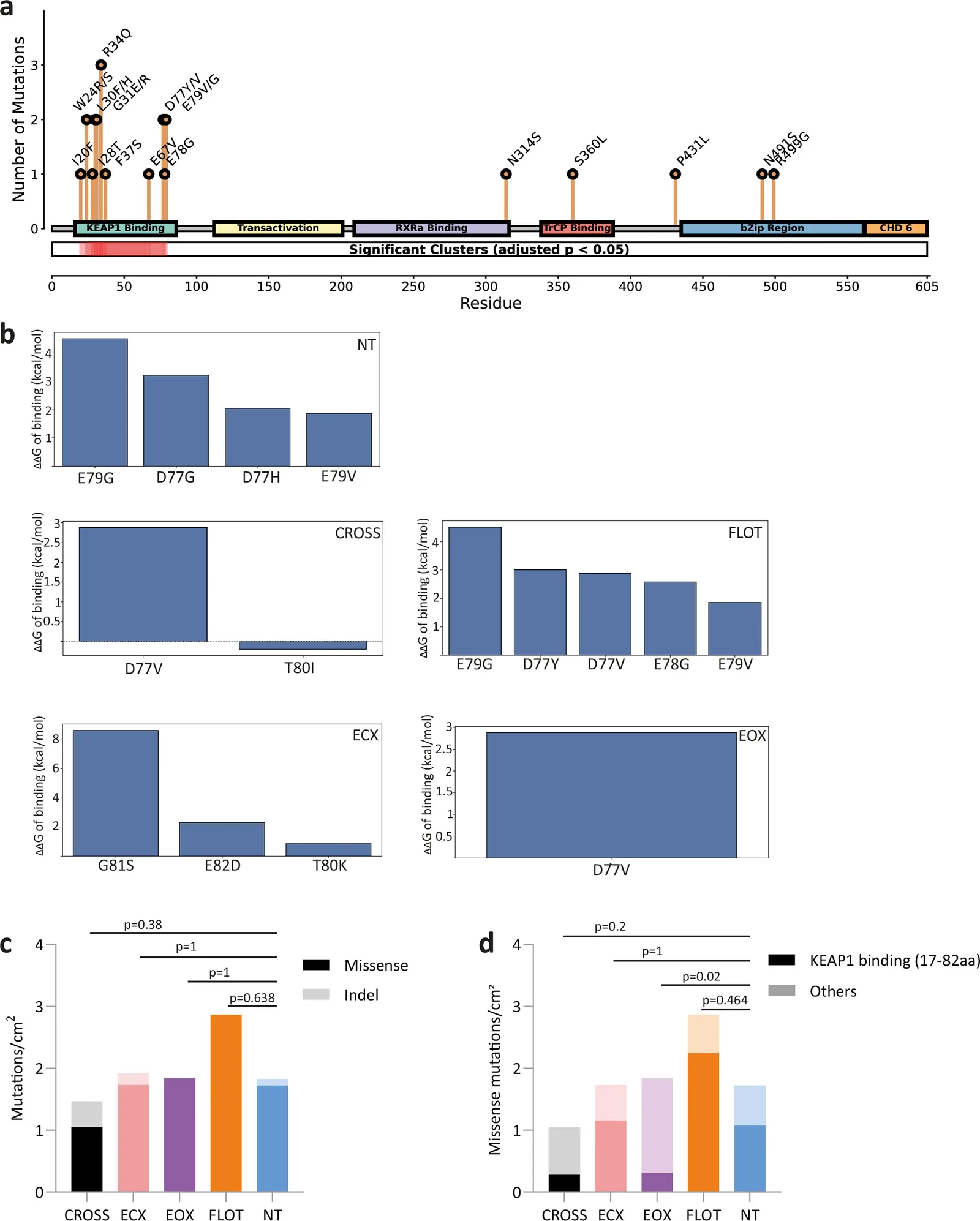
*NFE2L2* mutations. **a** Lollipop plot showing 1D clustering based on NMC (Methods) for NFE2L2 using missense mutations in the FLOT group. b Binding DDG of NFE2L2 for amino acids 71–87 involved in KEAP1 binding for each treatment cohort. **c**. Number of NFE2L2 mutations/cm2 of epithelia tissue in each of the treatment groups. CROSS n = 21, ECX n = 10, EOX n = 6, FLOT n = 23, NT n = 17 **c** Number of mutations/cm^2^ localized in the KEAP1 binding domain (defined as amino acids 17–82) and elsewhere (Others) NFE2L2 in each of the treatment groups CROSS n = 15, ECX n = 9, EOX n = 6, FLOT n = 23, NT n = 16. P values determined by two-tailed Fisher’s exact test. Source data

NFE2L2 is a transcription factor with a key role in regulating cellular responses to oxidative stress and has been linked to chemoresistance in a range of cancers^50,53^. The levels of NFE2L2 protein are tightly regulated by KEAP1, the primary ubiquitin ligase that mediates NFE2L2 degradation. We observed 1D clustering around the KEAP1 binding domain in the FLOT, ECX and NT groups, suggesting that selection of mutations in this domain occurs in normal esophagus (Extended Data Fig. 9a). This domain includes two highly conserved motifs—DLG and ETGE—which bind KEAP1 under homeostatic conditions, leading to the ubiquitination and degradation of NFE2L2. Nearly all mutations observed across the cohort showed a predicted increase ligand binding energy, therefore decreasing the likelihood of KEAP1 binding (Extended Data Fig. 9b). Mutations in *NFE2L2* that disrupt KEAP1 binding stabilize the protein, increase NFE2L2 transcriptional target gene expression and may increase cellular proliferation in the esophagus^53–56^. Most *NFE2L2* mutations we detected were missense, suggesting that complete loss of the protein does not confer a fitness advantage (Extended Data Fig. 9c). The FLOT group showed the most significant clustering (Extended Data Fig. 9a) with the highest density of mutations cm^−^^2^ (Extended Data Fig. 9c), that is, 78% of missense mutations occurring within the exDLG-ETGE KEAP1 binding region (amino acids 17–82) (Extended Data Fig. 9d), although this did not reach statistical significance. This suggests that GOF mutations in NFE2L2 may be selected in normal esophageal epithelium and that selection is further enhanced by FLOT chemotherapy.

**Fig. 6:**
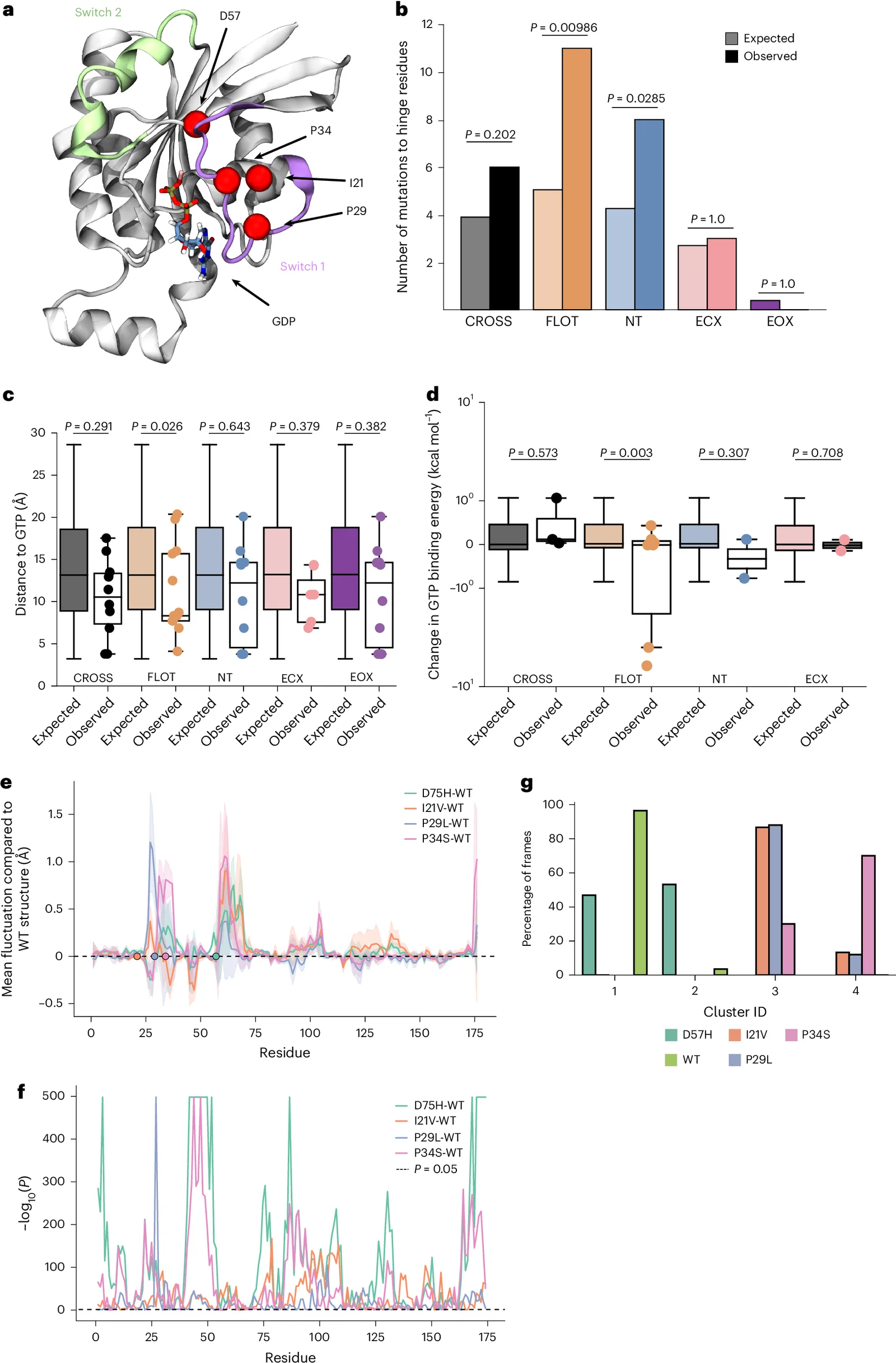
*RAC1* mutations with increased affinity for GTP are selected in the FLOT group. **a**, 3D structure of RAC1 highlighting the Switch domains (Extended Data Fig. 10). GTP in the binding pocket is shown. **b**, Observed number of mutations in the hinge regions in each treatment group compared to an expected distribution. *P*values were determined using a two-tailed analysis of variance. **c**, Distance of mutational sites to GTP in each condition. The *P*value represents a two-tailed Monte Carlo test applied to the median distance versus an expected distribution given the mutational signature. **d**, Change in energy of GTP binding (ΔΔG binding) for mutations observed in each condition versus an expected distribution. EOX mutations were too far from GTP to calculate the binding energy. The *P*value represents a two-tailed Monte Carlo test applied to mean ΔΔG binding for observed mutations versus an expected distribution based on the mutational signature. **e**, Molecular dynamic simulations showing mean fluctuation of mutant structures compared to WT. **f**, Comparison of root mean squared function between all mutant structures and WT. *P* = 0.05 is indicated by a dashed line. Flexibility of the Switch domain is significantly increased in all mutant structures. **g**, Clustering of molecular dynamic simulation frames according to mutant type. Mutant structures are significantly different from the WT (*P* < 10^−30^, chi-squared statistic = 33355). The box plot central line shows the median; the box indicates the quartiles. Whiskers extend to the furthest data point within 1.5 times the IQR of each box edge.

**Extended Data Fig. 10:**
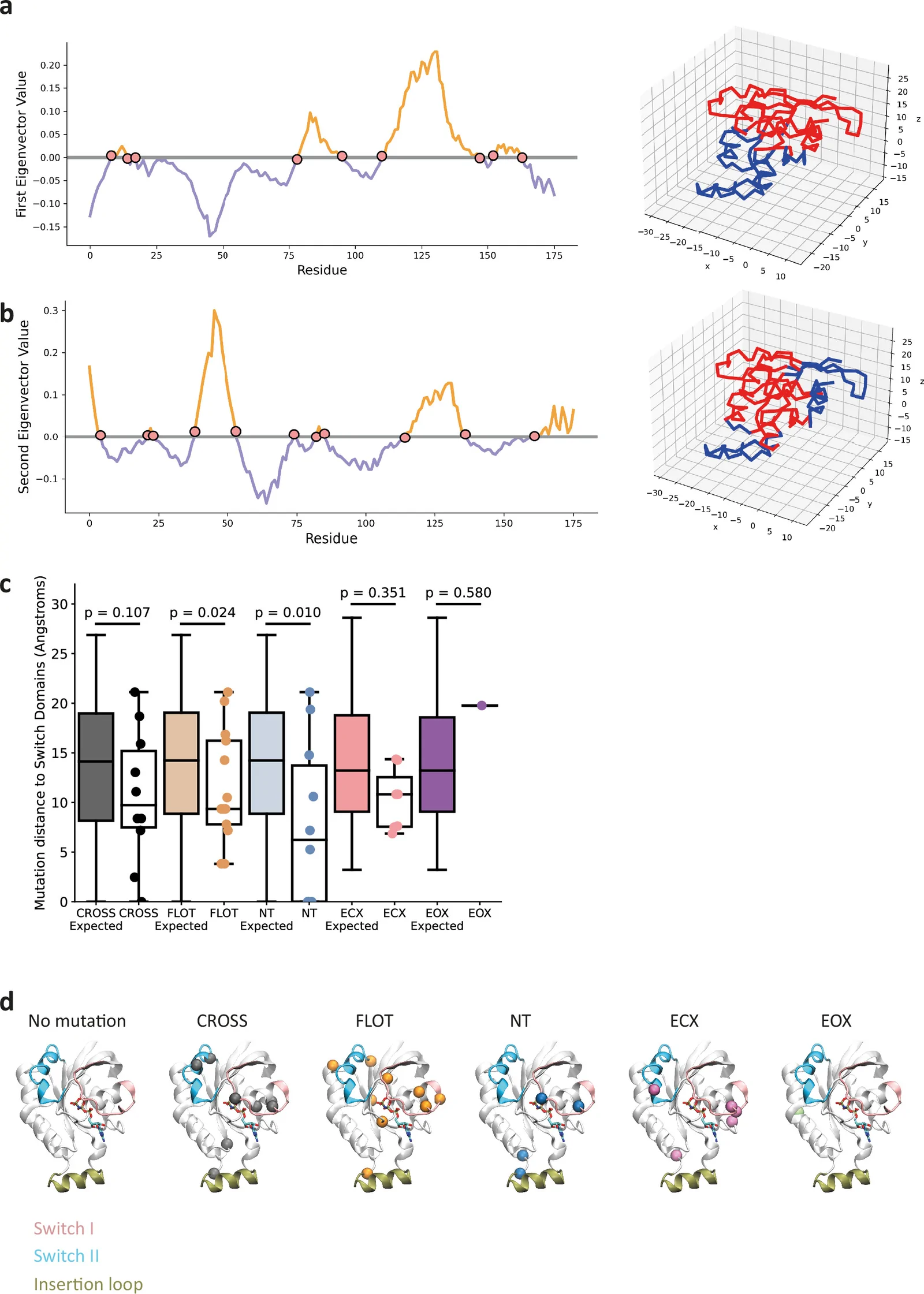
Effects of *RAC1* mutations. Elastic network modelling of RAC1 structure. The first eigenvector (**a**) describes the hinging of the protein through its center. The second eigenvector (**b**) identifies hinge amino acids (marked with dots) linking higher flexibility regions altering the ease/ restriction of GTP binding. **c** Distance of mutations to the Switch domains compared to expected distribution. P-value represents two-tailed Monte Carlo test applied to observed mutation distance vs an expected distribution based on the mutational signature for each treatment group. **d** 3D structure of RAC1 showing mutations in each treatment group. Box plot central line shows median and box indicates quartiles. Whiskers extend to furthest data point within 1.5 x inter quartile range of each box edge.

Next, we explored *RAC1* mutants in the FLOT group. *RAC1* is a member of the RHO family of GTPases that maintains the proliferative potential of wild-type (WT) keratinocyte stem cells and their ability to respond to injury^57,58^. RAC1 switches between a guanosine diphosphate-bound state to an active guanosine triphosphate (GTP)-bound state through the movement of two ‘Switch’ domains that open and close. Using computational elastic network modeling, we predicted the conformational dynamics of the protein (Methods). This technique showed two major movements within the protein, the first hinging within the central region of RAC1 (Extended Data Fig. 10a) and the second movement within the Switch domains (Extended Data Fig. 10b). The hinge points of this latter motion are amino acids that surround the GTP binding pocket (Fig. 6a). Changes within these hinge regions may alter the flexibility of the protein and binding of GTP through accessibility of the binding site^59^. Both the FLOT and NT groups showed significantly increased numbers of mutations within the hinge residues compared to an expected Monte Carlo sampled distribution based on the mutational spectrum of each treatment group (Fig. 6b). These two groups also showed significantly closer mutational distance to the Switch domains compared with an expected distribution based on the mutational signature (Extended Data Fig. 10c). This suggests that selection of mutations within these regions occurs in untreated esophageal epithelium. However, in FLOT patients, the mutant codons lie significantly closer to bound GTP (Fig. 6c) and have a significantly decreased binding energy compared to the neutral distribution (Fig. 6d). To assess the effects of individual mutations, we performed molecular dynamic simulations of RAC1 structures for the WT protein and for common mutants observed in the FLOT group—I21V, P29L, P34S and D57H. P29L is a known GOF mutation linked to cancer progression and its effect is induced by altering the dynamics of the Switch domains (Fig. 6e)^59–61^. A comparison of the root mean squared fluctuation of each mutated structure to the WT structure shows that the flexibility of the Switch domains in all four mutations is significantly increased compared to WT (Fig. 6f). Finally, we grouped every frame from our molecular dynamic simulations in four clusters as defined by *k*-means. This demonstrates that the WT simulation clusters differ significantly (chi-squared *P* = 0) from the mutated structures (Fig. 6g). These results are consistent with the hypothesis that mutations within the Switch regions can increase the flexibility of the hinge region to increase activity. We suggest that normal epithelium harbors a pool of *RAC1* mutations involving the hinge region but that treatment with FLOT selects those which may act through increased affinity of GTP for the binding site, although activating mechanisms involving altered conformational cycling, impaired hydrolysis or altered effector engagement are also possible.

**Fig. 7:**
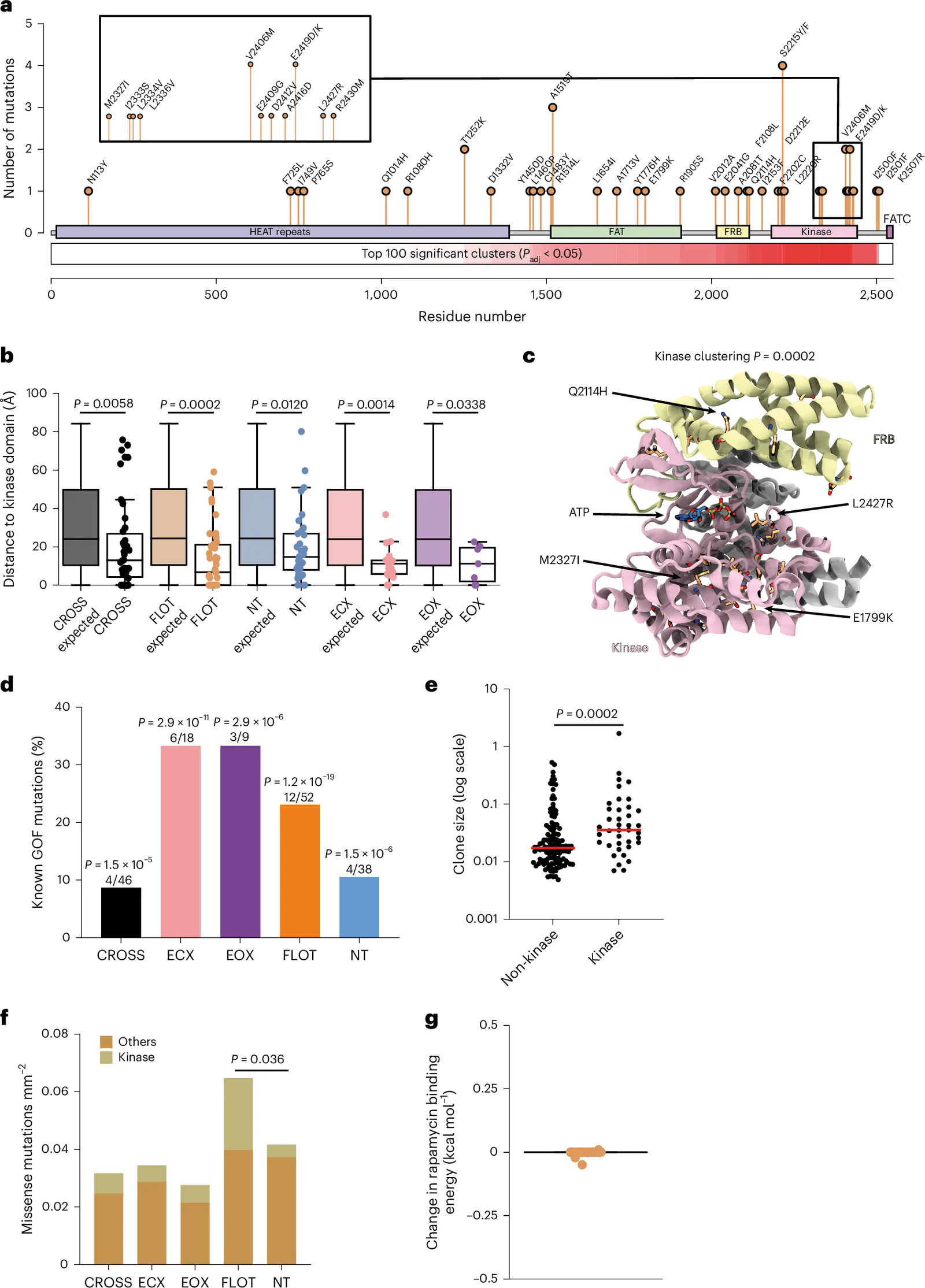
Positive selection of *MTOR* mutants in patients undergoing FLOT treatment. **a**, Lollipop plot of mutations in MTOR observed in patients exposed to FLOT. 1D clusters were calculated using NMC; the top 100 significant (*P*_adj_ < 0.05) clusters are plotted. **b**, Distance of mutational sites to the MTOR kinase domain in each condition. *P* represents a two-tailed Monte Carlo test applied to median distance versus an in silico expected distribution of mutations given the mutational signature. **c**, Structure of the MTOR kinase domain. Known GOF mutations are labeled. *P* represents a two-tailed Monte Carlo test applied to the median distance from the kinase domain versus an expected distribution given the mutational signature for the FLOT treatment group. **d**, Percentage of mutations in each condition that are known GOF mutations. *P* represents a two-tailed binomial test versus an expected distribution given the mutational signature. **e**, Clone size distribution (summed VAF) of missense mutations within and outside the kinase domain. The kinase domain is defined as amino acids 2,181–2,441. All conditions are pooled. The *P* value was calculated using a two-tailed Wilcoxon rank-sum test (*n* = 163 mutations). **f**, Number of *MTOR* missense mutations mm^−^^2^ of epithelia sequenced. Mutations are divided into those within the kinase domain and those outside (others). Two-tailed Fisher’s exact test with Benjamini–Hochberg multiple test correction. **g**, Changes in binding energy (ΔΔG binding) for rapamycin for every mutation observed in patients exposed to FLOT. The box plot central line shows the median; the box indicates the quartiles. Whiskers extend to the furthest data point within 1.5 times the IQR of each box edge. Source data

Finally, we investigated *MTOR* mutants. MTOR is a protein kinase that regulates cell growth and proliferation, so activating mutations would be expected to increase clone size^52^. 1D NMC and 3D structural modeling determined that the observed mutations were significantly closer to the kinase domain than the neutral distribution and that this was most significant in the FLOT group (Fig. 7a–c). A significant proportion of *MTOR* mutations in all treatment groups overlapped with known, experimentally validated GOF mutations (Fig. 7d); these activating mutations can arise though both direct kinase-site effects and allosteric relief of autoinhibition. Clones carrying kinase domain mutants are significantly larger than those with other missense mutations (Fig. 7e)^62–64^. The FLOT group showed an increased density of kinase missense mutations (Fig. 7f) compared to the other groups. Taken together, these findings suggest that activating mutations within the MTOR kinase domain drive clonal expansion in normal epithelium but are most strongly selected by FLOT treatment.

Finally, we investigated if the *MTOR* mutants seen in FLOT patients were likely to be druggable. Some *MTOR* mutants are inhibited by rapamycin but others are resistant^62,65^. We calculated the change in binding energy between WT MTOR and the *MTOR* mutations observed in the FLOT group. This analysis showed that the FLOT group mutants were not predicted to alter the binding affinity of rapamycin, suggesting that the selected clones would likely remain sensitive to inhibition by rapalogs (Fig. 7g).

## Discussion

These findings demonstrate that the mutational landscape of a normal epithelium that has evolved over decades may be altered dramatically in just a few weeks under the selective pressure of anticancer treatment. This leads to the selective expansion of preexisting mutant clones in certain treatment groups.

The absence of a chemotherapy mutational signature in both NanoSeq and whole-genome samples of normal epithelium is notable. In contrast, in other normal tissues of patients treated with chemotherapy, drug-induced mutations have been detected with duplex sequencing approaches as used here^66–69^. The mutational signatures observed in other tissues include those from platinum compounds, 5-FU and capecitabine, all of which were included in the treatment protocols used in this study^20,21,68,70^. The lack of detectable mutational signatures in the esophagus from treated individuals is unusual and may reflect loss of epithelial cells that acquire sufficient chemotherapy-induced damage because of cell death or differentiation and/or the short duration of neoadjuvant treatment.

The CROSS schedule results in a mutational landscape with double the area of *TP53* and *PPM1D* mutant epithelium compared with other groups. This is consistent with the effect of radiation in transgenic mice, in which repeated subtherapeutic exposures substantially expand *TP53* mutant clones^71^. Selection for *PPM1D* and *TP53* has been observed in other normal tissues, including the hematopoietic system, liver and lung in patients treated with radiation, platinum compounds, *TOP2A* inhibitors or immunotherapy^7–10,66^. We also observed more CNA at the *TP53* locus in CROSS patients. Biallelic *TP53* disruption is required for the development of most premalignant lesions and esophageal SCC (ESCC)^37–39^. In humans, a history of esophageal irradiation is associated with an increased risk of ESCC in survivors of breast cancer^72,73^. Our findings suggest radiation-induced expansion of *TP53* mutant clones, associated with *TP53* CNA, as a potential mechanism for increased cancer risk after esophageal radiation exposure and reinforce the case of minimizing normal tissue exposure during radiation treatment^74,75^.

The enhanced selection of mutants in a treatment-specific manner is notable. Activating mutants of *MTOR*, *RAC1* and *NFE2L2* were selected after treatment with FLOT, which differs from the schedules in containing 5-FU, whose activity is potentiated by leucovorin^76^. The selected mutants are GOF and may protect normal esophageal cells against 5-FU. Do these findings in normal tissue correlate with treatment resistance in cancer with its altered genome and microenvironment? MTOR and NFE2L2 activation is linked to 5-FU resistance in tumors and is associated with 5-FU resistance in cancer cells^55,56,77,78^. There are no reports linking *RAC1* mutants to 5-FU resistance clinically, although expression of the RAC1b isoform confers 5-FU resistance in vitro^4^. However, while it cannot be assumed that mutant selection in normal tissues will be the same in cancer cells, identifying mutants selected by treatment in normal tissues may provide a catalog of potential targets that modulate cellular resilience to therapy. These in turn may guide investigation of tumor resistance. More generally, even small areas of epithelium like the esophagus may carry many thousands of mutant clones, offering substantial statistical power for the study of treatment-induced selection in humans^2,27,79^.

Limitations of this study include the relatively small number of patients per treatment group and the available meta-data concerning environmental exposures. We found significant associations between some measures of the mutational landscape and treatment but larger studies will be needed to explore whether factors such as age, sex and smoking interact with treatment to shape the normal tissue mutational landscape in patients with esophageal cancer. We were unable to detect negative selection in this study (defined as *d*N/*d*S < 1). Detecting negative selection requires sufficient synonymous mutations to reach significance. We only detected 3,669 synonymous mutations across all 324 genes and speculate that the study was underpowered to find negative selection.

Sequencing of normal tissues has the potential to uncover altered selection after a wide range of cancer treatments, even if only given for a short duration. Treatment may enhance the selection of mutants whose fitness was neutral or only weakly positive before therapy. The significance of missense mutations can be further explored by extending the analysis to high-confidence-predicted protein structures. Thus, mutants in normal tissues provide in vivo evidence of actionable targets for mitigating normal tissue toxicity and may inform strategies for overcoming drug resistance in tumors.

## Methods

### Tissue collection and ethical approval

Esophageal tissue was collected at San Raffaele Research Hospital (Milan, Italy) from patients who had recently undergone neoadjuvant or no treatment regime for esophageal cancer and Southampton General Hospital (UK). After recovery from treatment, patients underwent standard of care esophagectomy with 5-cm margins of esophageal tissue along with the tumor to remove all cancerous tissue and ensure clear surgical margins. The San Raffaele Hospital Oncology board made therapy decisions based on patients’ clinical condition and the current European Society for Medical Oncology Clinical Practice Guideline for esophageal cancer treatment^15,81^. Written informed consent was obtained from all participants. Ethical approval for patients from Milan was under study protocol no. ESO-CA001, approved by the San Raffaele Research Hospital Institutional Ethics Committee. Ethical approval for patients from Southampton was obtained under Local Research Ethics Committee no. 09/H0504/66, approved by the Southampton and South West Hampshire Research Ethics Committee, UK. Sequencing was performed at the Wellcome Trust Sanger Institute under ethical approval no. 15/EE/0218 from the East of England-Cambridge East Research Ethics Committee.

Details of patient demographics can be found in Supplementary Table 2.

A full-thickness segment of mid-distal esophagus was excised. Esophageal samples were opened longitudinally and the muscle and submucosa removed. Only tissue above the gastroesophageal junction was used. Samples were cut into approximately 0.5 × 0.5-cm pieces and incubated in 20 mM EDTA at 37 °C for 2 h. After this, the epithelium was peeled away from the remaining submucosa using fine forceps. The epithelium was fixed in 4% paraformaldehyde (Sigma-Aldrich) for 30 min before being washed three times in 1× PBS.

For sequencing, the esophageal epithelium was cut into a gridded array of 2-mm^2^ samples and DNA extracted using the QIAMP DNA microkit (QIAGEN) by digesting overnight and according to the manufacturer’s instructions. DNA was eluted using prewarmed AE buffer where the first eluent was passed through the column two further times. For the biopsy and resection samples (PD59778–PD63256) samples were not peeled but were cut into 2-mm^2^ samples and extracted as described. Samples PD47498–PD50080 used flash-frozen esophageal muscle DNA as the germline control; samples PD59778–PD59811 used serum as the germline control. In both cases, DNA was extracted as for the epithelial samples.

For WGS, 0.25-mm diameter samples of the epithelium were collected using a brain punch biopsy (Stoelting). DNA was extracted using the Arcturus PicoPure Kit (Applied Biosystems) according to the manufacturer’s instructions.

### Wholemount staining

A subset of epithelial tissue was stained to ensure that the tissue had normal histology. For this, paraformaldehyde-fixed wholemounts were blocked for 3 h in blocking buffer (0.5% BSA, 0.25% fish skin gelatin, 0.5% Triton X-100 and 10% donkey serum) dissolved in PHEM buffer (60 mM PIPES, 25 mM HEPES, 10 mM EGTA and 4 mM MgSO_4_ × 7H_2_0). Wholemounts were costained with Ki-67 (cat. no. ab15580, Abcam) and Alexa Fluor m488 wheat germ agglutinin. Both primary antibodies were diluted 1:500 in blocking buffer and incubated overnight at room temperature with continuous rocking. Samples were washed for a minimum of 48 h with 0.2% Tween-20 in PHEM buffer changing daytime washes every 2–3 h. Tissue was then stained with Alexa Fluor 555 anti-rabbit (1:1,000), Alexa Fluor 488 wheat germ agglutinin (1:500) and TO-PRO-3 647 (1:3,000); samples were incubated overnight at room temperature with continuous rocking. Samples were washed for a minimum of 48 h with 0.2% Tween-20 in PHEM buffer changing daytime washes every 2–3 h. Samples were mounted with 50% glycerol before imaging on a Leica SP8 confocal microscope (Leica Microsystems).

### CRISPR–*Cas9* analysis of *Ppm1d* mutants in mouse esophageal epithelioids

The methods are described in the Supplementary Note. gRNA sequences are listed in Supplementary Table 19.

### DNA sequencing

#### Targeted sequencing

In this study, we used two Agilent SureSelect custom-designed bait panels; one has been described previously^2,82^ and includes many genes implicated in cancer. A list of genes covered by the bait set can be found in Supplementary Table 7.

A total of 200 ng of genomic DNA was fragmented (average size distribution ~150 bp, LE220, Covaris), purified, libraries prepared (NEBNext Ultra II DNA Library Prep Kit, New England Biolabs) and index tags applied (Sanger 168-tag set). Index-tagged samples were amplified (six cycles of PCR, KAPA HiFi kit, KAPA Biosystems), quantified (Accuclear dsDNA Quantitation Solution, Biotium) then pooled together in equimolar fashion; 500 ng of pooled material was taken forward for hybridization, capture and enrichment (SureSelect Target Enrichment System, Agilent Technologies, normalized to ~6 nM), and submitted to cluster formation for sequencing on a NovaSeq 6000 system (Illumina) to generate 100-bp paired-end reads.

BAM files were mapped to the GRCh37d5 reference genome using the Burrows–Wheeler Aligner-MEM (v.0.7.17)^83^. Duplicate reads were marked using SAMtools (v.1.11)^84^. Depth of coverage was also calculated using SAMtools to exclude reads which were unmapped, not in the primary alignment, failing platform or vendor quality checks, or were PCR/optical duplicates. BEDTools (v.2.23.0) was then used to calculate the depth of coverage per base across samples (Extended Data Fig. 5a^85^). Samples with sequencing coverage less than 250× or more than 800× were excluded from the downstream analysis. We saw no association between the number of mutations per kb per gene and the mean gene sequencing coverage (*R*^2^ = 0.01).

#### WGS

The NEBNext Ultra II Fragmentase System was used for punched epithelial sample shearing and library preparation. DNA was hybridized to the same custom bait panel (as described for targeted sequencing). Material was subjected to 12 PCR cycles (initial denaturation: 95 °C for 5 min, 98 °C for 30 s, 65 °C for 30 s, 72 °C for 2 min 12 cycles, 72 °C for 10 min). Variants were called using the CaVEMan (v.1.15.1) and Pindel (v.3.7.0) algorithms^86,87^. For single-nucleotide variants, CaVEMan was run with the major copy number set to ten and the minor copy number set to two. Only single-nucleotide variants that passed all CaVEMan filters were kept. Samples were then put forward for WGS if they carried a nonsynonymous mutation with VAF > 0.35.

#### NanoSeq

A total of 50 ng DNA was used for duplex sequencing library preparation according to Abascal et al.^17^. Briefly, DNA was digested with mung bean nuclease, A-tailed, repaired and tagged. Then, 0.3 fmol of indexed tagged library were sequenced with 14 PCR cycles before quantifying and sequencing on a NovaSeq 6000 system (Illumina) with 150-bp paired-end reads to give a median 0.9× duplex coverage; 30× coverage whole-genome sequence of esophageal muscle from the same patient was used as the germline control for calling of single-nucleotide polymorphisms and indels. Only samples with a verifyBAMID alpha freemix less than 0.005 were used in the downstream analysis. For both SNVs and indels, only calls that passed all the defined filters were used (https://github.com/cancerit/NanoSeq). The germline sample from patient PD59796 failed NanoSeq library preparation. Therefore, this patient was excluded from the NanoSeq analysis.

#### Mutation calling

For targeted sequencing data, subclonal mutation variant calling was made using the deepSNV R package (also commonly referred to as ShearwaterML), v.1.21.3, available at https://github.com/gerstung-lab/deepSNV, used in conjunction with R v.3.3.0 (2016-05-03)^2^. Variants were annotated using VAGrENT^88^. Mutations called by ShearwaterML were filtered using the following criteria: the positions of called SNVs must have a coverage of at least 100 reads; germline variants called from the same individual were removed from the list of called variants. Samples PD63225–PD63256 did not have an associated germline sample. For these patients germline variants were filtered if they appeared in all the grid samples from the same individual with VAF > 0.3; adjustment for the false discovery rate and mutations demanded support from at least one read from both strands for the mutations identified; pairs of SNVs on adjacent nucleotides within the same sample were merged into a dinucleotide variant if at least 90% of the mapped DNA reads containing at least one of the SNV pair, contained both SNVs; identical mutations found in multiple contiguous tissue biopsies were merged and considered as a single clone to prevent duplicate clone counting; and ShearwaterML was run with a normal panel of approximately 19k reads.

#### Mutational signature analysis

Mutational matrices for SBS were generated using SigProfilerMatrixGenerator with default options (v.1.2.12). SBS signatures were extracted using SigProfiler extractor (v.1.1.12) with default parameters; the ndsvd_min initialization algorithm used was nndvsd_min and the number of NMF replicates was 500. De novo SBS signatures were extracted within the SBS288 context. For the NanoSeq analysis, the optimal number of signatures using the mean sample cosine distance and average stability was determined to be two: SBS288A and SBS288B. Deconvolving these signatures onto the known COSMIC signatures (v.3.4) (cancer.sanger.ac.uk/signatures) using SigprofilerAssignment (v.0.0.13) showed that signature A included a combination of SBS1 (10%), SBS5 (49%) and SBS40a (41%) (cosine similarity 0.97), whereas signature B consisted of a combination of SBS1 (4%), SBS5 (17%), SBS16 (24%) and SBS40a (54%) (cosine similarity 0.98). For the genome samples, signatures were extracted in the 1,536 context; all other parameters remained the same.

Mutational signature presence was tested using mSigAct v.3.0.1 (https://github.com/steverozen/mSigAct), with SBS96 mutational spectra generated from PASS-filtered variant call format using ICAMS v.3.0.12 (VCFsToCatalogs, reference genome hg19, genome region)^22^. Reference signatures were taken from COSMIC v.3.3 (GRCh37, SBS96; cosmicsig).

For each sample, four signatures were included as a fixed background set that was always available to the model: SBS1, SBS5, SBS16 and SBS40. Each of four candidate signatures—SBS17b, SBS25, SBS31 and SBS35—was then tested independently by adding it to this background set and applying the SignaturePresenceTest function. This performs a likelihood ratio test comparing the maximum-likelihood reconstruction of the spectrum with versus without the candidate signature, with the test statistic evaluated against a chi-squared distribution (1 d.f.); the null hypothesis is that the spectrum can be reconstructed without the candidate signature. A multinomial likelihood was used (m.opts = DefaultManyOpts(likelihood.dis = ‘multinom’)). The reconstruction was significantly improved by a candidate signature at *P* < 0.05 after Bonferroni correction applied across all sample × signature tests.

#### Copy number calling

For WGS data, the copy number was inferred using ascatNgs, using esophageal muscle from the same patient as a germline control^89^. For samples PD63225–PD63256 without a germline sample, a random 2-mm^2^ grid sample was used as the germline.

The copy number of each 2-mm^2^ grid using the targeted data was called using ASCAT (v.3.2.0). Captured regions were specified, allowing only SNPs in those regions to be identified using the normal samples. Allele counts were generated for SNP positions from these normal samples allowing calling for both 2-mm^2^ grid and germline samples. These were used to derive logR and BAF values used to generate segment calls of allele-specific copy number (https://github.com/VanLoo-lab/ascat). Patient PD49363 showed considerable stretches of homozygosity throughout the genome, making copy number calling difficult. This patient was excluded from this analysis.

#### Calculations of percentage mutant epithelia

In this study, the percentage of mutant tissue and *d*N-to-*d*S analysis was performed as described previously^2^(https://github.com/im3sanger/dndscv).

#### Statistics and reproducibility

No statistical method was used to predetermine sample size. Experiments were not randomized. Investigators were not blinded to patient treatment regime. All samples were included in the analysis.

In general, *P* < 0.05 was considered statistically significant, but for the *d*N-to-*d*S ratios calculated using dNdScv, a global *q* < 0.01 was considered statistically significant. To reduce a probability of artificially inflated *d*N-to-*d*S ratios in genes for which there were no synonymous mutations^90,91^, we filtered such genes if the total number of nonsynonymous mutations was eight or fewer. This cutoff is based on estimating the probability of observing no synonymous mutations in a sequence of mutations; hence the likelihood of the *d*N-to-*d*S ratio being artificially inflated. This requires estimating the probability of a ‘non-event’. It is possible to put an upper confidence limit on event risk, when no events are observed in a sequence of trials. A simple ‘rule of three’ has been proposed, such that if no events are observed in a group, then the upper confidence interval limit for the occurrence of events is three, and the risk is 3/*N*, where *N* is the sample size of the group^92^. The value 3 coincides with the upper limit of a one-tailed 95% confidence interval (CI) from the Poisson distribution. It is 3.7 for the 97.5% CI and 4.6 for the 99% CI. Based on this, we estimated that the minimum number of nonsynonymous mutations should be at least eight for 99% confidence (1–0.01) that a *d*N/*d*S value is reliable if there are zero synonymous mutations.

All significance tests were two-tailed unless otherwise stated. The numbers of mutations or patients are given in the figure legends and source data tables.

For the calculation of statistics related to structural mutational selection, we used the ‘Darwinian shift’ method^93^, via the Python implementation available at https://github.com/michaelhall28/darwinian_shift. This is detailed further in the protein structure section.

#### 1D clustering analysis

To identify the positional clustering of missense mutations along the primary amino acid sequence, we performed a 1D NMC analysis on positively selected genes within each treatment group separately. Only nonsynonymous missense mutations were included in this analysis. We performed NMC clustering using the NMC function within the iPAC R package^28^. Clustering significance was assessed against the null hypothesis that mutations were randomly distributed along the coding sequence; multiple-testing correction was applied within each gene and treatment-group analysis. Clusters with *P*_adj_ < 0.05 were considered significant. For visualization, lollipop plots show the significant clusters identified using NMC.

#### Structural mutational selection analysis

Protein structures were downloaded from the Protein Data Bank^94^. Statistical tests of mutation enrichment were applied using the Python library Darwinian_shift^93^, available at https://github.com/michaelhall28/darwinian_shift. We performed calculation of selection by comparing observed properties with those expected if only the mutational signature was occurring in the tissue. We have applied this method previously to a selection of mutant clones in the skin^27^. Mutations observed in each group of patients (CROSS, ECX, EOX FLOT or NT) were used to calculate the mutational spectrum for each condition. For each gene studied, the calculated signature was then used to generate an expected distribution for the mutational property being investigated. For example, for mutations observed in the kinase domain, the number of mutations expected in the kinase domain was calculated by taking the mutational spectrum, total number of mutations observed in that gene and the DNA sequence of the kinase domain. This allows the generation of how many mutations would be expected to be seen in the kinase domain if the only process occurring was mutation consistent with the mutational spectrum. This expected number was then compared to the observed and a *P* value was generated using a binomial test. For *P* values relating to continuous metrics, we used a Monte Carlo test applied to distances (using differences in median distance to a defined part of the structure, such as a small molecule to calculate the *P* value) and binding energy calculations (using differences in mean energy to calculate a *P* value). All code used to generate these calculations is available at https://github.com/shorthouse-lab/fowler_selection (ref. ^80^).

#### Energy calculations

Energy calculations were performed using FoldX^95^. We calculated every possible mutation for folding energies, and every possible mutation within 10 Å of the ligand for binding energies. The code for calculating the binding energies is available at https://github.com/shorthouse-lab/binding_ddg (ref. ^96^). These energies were then used to construct the expected distribution. To generate rapamycin parameters for use with FoldX, we used pyFoldX^97^. Ligand parameters were generated according to a protocol described previously in which each atom within the ligand is mapped to a chemically matched FoldX recognized atom name; coordinates from structures are used to build the ligand file that is recognized by FoldX^96^.

#### Elastic network modeling

Elastic network analysis was performed in Python using the ProDy package^98^. In this framework, a protein structure is represented as a simplified network in which residues are treated as nodes positioned at their Cα atoms. Pairs of nodes that lie within a specified interaction distance are connected by harmonic springs, allowing the elastic network to approximate the intrinsic mechanical flexibility of the protein. For this study, node interactions were defined using a distance cutoff of 1 nm.

Normal mode analysis was then applied to the resulting network to calculate the collective motions accessible to the structure. The eigenvectors derived from this analysis describe the dominant displacement patterns of residues relative to one another; therefore, they capture the large-scale conformational dynamics predicted for the protein. These low-frequency modes were used to identify regions of coordinated motion and hinge points within the structure.

The scripts used to construct and analyze the elastic network models are available from the GitHub repository associated with this manuscript and follow the general workflow described in the ProDy documentation (www.bahargroup.org/prody/tutorials/enm_analysis/gnm.html).

#### Molecular dynamics simulations

Molecular dynamics simulations were performed using Gromacs v.2023.3 (ref. ^99^). The WT structure of RAC1 (Protein Data Bank ID: 5N60) was mutated using the FoldX BuildModel^95^ Each protein variant was parameterized using the AMBER99SB-ILDN force field^100^. The system was solvated in a triclinic box with at least 10 Å between the protein and the box edges using the TIP3P water model. Counter ions (Na^+^/Cl^−^) were added to neutralize the system and adjust the ionic strength to 150 mM NaCl.

Energy minimization was performed using the steepest descent algorithm until the maximum force fell below 1,000 kJ mol^−1^ nm^−1^. The system was then equilibrated with: (1) NVT equilibration for 100 ps at 310 K using the V-rescale thermostat, followed by (2) NPT equilibration for 100 ps at 1 bar using the Parrinello–Rahman barostat. Position restraints were applied to all heavy atoms during equilibration.

Production molecular dynamic simulations were then run for 200 ns for each system under periodic boundary conditions with a 2-fs time step. Three repeats with different random seeds were run for each protein. Long-range electrostatics were treated with the particle mesh Ewald method with a cutoff of 1.0 nm. The LINCS algorithm was used to constrain all bonds to hydrogen atoms.

Analysis was performed using mdanalysis (www.mdanalysis.org)^101,102^. For each simulation, trajectories were first aligned to the starting structure using backbone atoms to remove global translational and rotational motion. Root mean square fluctuation values were then calculated per residue from the aligned trajectories; mutant trajectories were compared with WT to assess local changes in flexibility, particularly within the RAC1 Switch regions.

To compare conformational state occupancy between WT and mutant *RAC1* simulations, frames were represented by aligned Cα coordinates of the RAC1 backbone and partitioned into four clusters using *k*-means clustering. Differences in cluster occupancy between WT and mutant proteins were assessed using a chi-squared test on the resulting contingency table.

### Reporting summary

Further information on research design is available in the Nature Portfolio Reporting Summary linked to this article.

## Online content

Any methods, additional references, Nature Portfolio reporting summaries, source data, extended data, supplementary information, acknowledgements, peer review information; details of author contributions and competing interests; and statements of data and code availability are available at https://doi.org/10.1038/s41588-026-02738-0.

## Supplementary information

Supplementary Information Supplementary Note

Reporting Summary

Peer Review file

Supplementary Tables Supplementary Tables 1–19.

## Source data

Source Data Fig. 1 Source data.

Source Data Fig. 2 Source data.

Source Data Fig. 3 Source data.

Source Data Fig. 4 Source data.

Source Data Fig. 5 Source data.

Source Data Fig. 7 Source data.

Source Data Extended Data Fig. 1 Source data.

Source Data Extended Data Fig. 3 Source data.

Source Data Extended Data Fig. 4 Source data.

Source Data Extended Data Fig. 5 Source data.

Source Data Extended Data Fig. 6 Source data.

Source Data Extended Data Fig. 7 Source data.

Source Data Extended Data Fig. 9 Source data.

## Data Availability

The sequencing datasets generated during the study will be available at the European Genome-phenome Archive with the following accession numbers: EGAD00001011181 (WGS); EGAD00001011182 (targeted sequencing from 0.25-mm samples); EGAD00001011183 (NanoSeq); and EGAD00001011184 (targeted sequencing from 2-mm^2^ samples). Data are accessible to researchers by contacting datasharing@sanger.ac.uk. Source data are provided with this paper.

## Code availability

The pipeline for the analysis of the CRISPR–Cas9 experiments is available at at https://github.com/PHJonesGroup/Fowler_etal_code, https://zenodo.org/records/21495557 and Zenodo (https://doi.org/10.5281/zenodo.21495556)^103^. The code used to perform the analysis of selection, along with notebooks containing the figures generated from the raw data, is available at https://github.com/shorthouse-lab/fowler_selection, https://zenodo.org/records/21505780 and at Zenodo (https://doi.org/10.5281/zenodo.21505780)^80^.
