# Supplementary material for: Cancer treatment alters mutant selection in normal esophagus
Source: Nat Genet. 2026 Sep 11;58(10):2566–78. doi: 10.1038/s41588-026-02738-0 (PMC13649571; doi:10.1038/s41588-026-02738-0)
Supplement: Supplementary file 1 [file 41588_2026_2738_MOESM1_ESM.pdf]

nature genetics

Supplementary information https://doi.org/10.1038/s41588-026-02738-0

Cancer treatment alters mutant selection in normal esophagus

In the format provided by the authors and unedited

This note describes the methods used in CRISPR/*Cas9* experiments to study *Ppm1d* mutant selection in mouse esophageal epithelioid cultures (Section 1) and statistical analyses of potential cofounding features in the data set (Section 2).

Section 1: Methods for CRISPR/Cas9 experiments

This section details methods for the experiments shown in Figure 4 c-e and described in the main text.

gRNA library design and synthesis

A minipool gRNA library was designed against genes showing changes in fitness under chemotherapy or radiation. gRNA sequences were selected from the Brie library or designed using chop-chop tool v3 (https://chopchop.cbu.uib.no , **Supplementary Table 19**) [1, 2].

A 90-mer oligo pool (oPool) was purchased from Twist Bioscience in the format ATATATCTTGTGGAAAGGACGAAACACCGN20GTTTAAGAGCTATGCTGGAAACAGCATAGCAAGTTTAAATA, where N20 specifies the variable gRNA sequence. gRNAs were PCR-amplified from the oPool under the following conditions:

**Component**	**25 µl Reaction**	**Final concentration**
2X Q5 Mastermix (NEB M0492)	12.5 µl	1X
10 µM Forward Primer	1.25 µl	0.5 µM
10 µM Reverse Primer	1.25 µl	0.5 µM
Template DNA (1ng/µl)	1.0 µl	0.04ng/ul
Nuclease-Free Water	9.0 µl	Up to 25 µl

**Step**	**Temperature**	**Time**
Initial Denaturation	98°C	30s
14 Cycles	98°C	10s
	67°C	10s
	72°C	15s
Final Extension	72°C	2mins
Hold	4°C	Hold

Forward Primer:
ATCATATGCTTACCGTAACTTGAAAGTATTTCGATTTCTTGGCTTTATATATCTTGTGGAAAGGACGAAACACC
Reverse Primer:
TGCCACTTTTTCAAGTTGATAACGGACTAGCCTTATTTAAACTTGCTATGCTGTTTCCAGCATAGCTCTTAAAC

KLV2-U6gRNA5(BbsI)-ccdb-PGKpuro2ABFP-W vector was digested with Fastdigest BbsI (Fermentas) for 1h at 37°C. The vector digest and PCR products (4 pooled 25 µl PCR reactions) were purified using the QIAquick PCR purification kit (Qiagen)) according to the manufacturer's instructions.

Gibson assembly reactions were set up using 100ng each of the PCR-amplified oligo library and the BbsI-digested gRNA vector and incubated at 50°C for 1 hour. Gibson assembly products were purified and concentrated by ethanol precipitation (1h at 4 °C) and resuspended in RNAse-free water to a final concentration of 100 ng/µl. 100µl of Lucigen Endura electrocompetent cells were combined with 1 µl of Gibson assembly product and electroporated using the BTX ECM630 (Harvard Apparatus) (2000 V, 200 Ω, 25 µF). Cells were recovered in 1 ml of Lucigen recovery medium for 1 hour at 37°C. Bacterial plates were prepared using a serial dilution of recovered cells to estimate the total number of transformants and calculate library coverage. The remaining

cells were grown overnight in a 250 ml low-salt 2 x LB + ampicillin liquid culture at 37°C. Plasmid DNA was extracted using the Endofree Maxi Kit (Qiagen) according to the manufacturer's instructions. The 680 gRNA library was transformed at an estimated coverage of >100,000X.

Lentivirus production

Lentiviruses were generated by transfection of $HEK293^{FT}$ cells using Lipofectamine 3000 (Invitrogen). One day before transfection, 4 million $HEK293^{FT}$ cells were plated in a 10cm dish to reach 80 – 90% confluence after 24h. On the day of transfection, 6 µg of lentiviral vector, 7.5 µg of the 2nd generation lentiviral packaging plasmid psPAX2, 2.5 µg of VSV-G envelope expressing plasmid pMD2.G and 35µl of P3000 Enhancer were added to 1.5ml of serum-free OptiMEM (Invitrogen). 41 µl of Lipofectamine 3000 was diluted in 1.5ml serum-free OptiMEM and incubated at room temperature for 5 minutes, before combining with the prepared DNA mixture and incubating at room temperature for a further 30 minutes. $HEK293^{FT}$ media was replaced with 6ml serum-free OptiMEM, before adding the transfection complexes. After 6 hours, the transfection medium was replaced with complete DMEM. Lentiviral supernatants were collected 72hours post-transfection. Harvests were centrifuged to remove cellular debris and filtered (0.45µm pore, PES)(Merck). Virus aliquots were stored at -80 °C.

Animals

No experiments were performed on live animals. Rosa26-Cas9 knockin mice (JAX laboratories, RRID:IMSR_JAX:026179) were bred and culled to harvest esophageal tissue for cell cultures[3]. Mice were housed in Specific and Opportunistic Pathogen Free unit at Charles River UK Ltd, Margate, Kent UK. A 12-hour light/12-hour dark cycle was used. Lights were switched off at 19:30 and there was no twilight period. Ambient temperature was 21 ± 2 °C, and humidity 55 ± 10%. Mice were kept at 3–5 mice per cage, in cages measuring 365 mm × 207 mm × 140 mm (length × width × height), with a floor area, of 530 cm^2. Individually ventilated caging (Tecniplast, Sealsafe 1284L) with 60 air changes/ hour was used. As well as Aspen bedding substrate, environmental enrichment with Nestlets, a cardboard tunnel and chew blocks was provided. Water and standard chow diet were provided ad libitum. Experiments were carried out with male and female animals, and no sex specific differences were observed. Animals were randomly assigned to experimental groups.

Epithelioid culture and CRISPR screening

Epithelioid cultures were set up from oesophageal epithelium from 4 Rosa26-Cas9 knockin mice (JAX laboratories, RRID:IMSR_JAX:026179) as described[3]. Briefly pieces of oesophageal epithelium were placed on transparent ThinCert (0.4 µm pore size, Grenier 657640) with the epithelium facing upwards. FAD (1:1 mix DMEM/F12 (Invitrogen cat no. 31330-038) and DMEM (Invitrogen 19971-025) and 5% fetal calf serum, 1% Penicillin/Streptomycin) was supplemented with 5µg/ml insulin (Sigma-Aldrich I5500), 18mM adenine (Sigma-Aldrich A3159), 100 pM cholera toxin (Sigma-Aldrich C8052), 10ng/ml EGF (PeproTech 100-15), 0.5 µg/ml hydrocortisone (Calbiochem 386698) and 5µg/ml apo-transferrin (Sigma Aldrich T2036) (cFAD). 6 x 4.5 cm^2 ThinCerts were set up per mouse and cells were maintained in cFAD until confluence.

For lentiviral gRNA transduction, cells were trypsinized with TrypLE (Thermo Fisher Scientific) for 30 mins at 37 ^oC. After centrifugation cells were resuspended in cFAD containing lentiviral gRNA library and 6-10 µM (final concentration) Y-27632 and re-seeded 6 x 4.5 cm^2 ThinCerts per mouse. Viral media was removed the following day and cells continued to be cultured in cFAD. For time point 0 cells were trypsinised 4 days after infection. Genomic DNA was extracted from one fifth of the cells using DNeasy Blood & Tissue kit (Qiagen). The remaining of the cells were replated on 3 x 4.5cm^2 inserts per sample and cultured under different conditions for three weeks. Cells were either untreated, treated with either 5 µM 5-Fluorouracil or 1 Gy ionising radiation/ week (total dose 3Gy). After this time cells were collected by trypsinization and genomic DNA was extracted. Transduction efficiency was determined by flow cytometry at time point 0. The Blue Fluorescent Protein (BFP) + cell population was also measured at the end point of the experiment (see Table 1 below).

		3 week timepoint		
Sample	Timepoint 0	Untreated	Ionising radiation	5FU
1	29.48	34.61	36.77	47.43
2	22.33	24.65	28.08	34.58
3	22.81	27.78	31.14	38.53
4	22.62	23.76	27.8	32.71

Table 1: Percentage BFP positive cells as assessed by flow cytometry

PCR amplification, Illumina sequencing (20-bp single-end sequencing with custom primers on the MiSeq platform) and sgRNA counting were performed as described previously[4].

CRISPR/*Cas9* analysis

We used MAGeCK for its well-established QC metrics and some statistical analysis[5]. However, MAGeCK's p-values can be unreliable in targeted CRISPR screens due to assumptions about sgRNA distribution not being met.

We therefore developed an alternative method to determine significant gRNA enrichment/depletion accounting for both FDR and FNR rates. Briefly, we determined the likelihood of the observed gRNA fold change for each gene differing from the distribution of the set of true neutral control gRNAs in the screen. The code is available on https://github.com/PHJonesGroup/Fowler_etal_code , and zenodo, https://zenodo.org/records/21495557 .

Section 2: Potential cofounding features in the data set

2.1 Associations between treatment type and mutational measurements

Our analysis reveals apparent relationships between treatment and mutational measurements observed in normal esophageal tissue in 69 patients with cancer. To explore whether this association could arise from other patient features, we analysed the associations between different *factors* (characteristics of the patient) and *responses* (measured mutational features). Factors in our study are treatment, age, gender, smoking, tumour type, and tumour location. The responses are Substitutions/Mb, and Indel/Mb, and the signatures SBS288A/Mb and SBS288B/Mb for Nanoseq data. For targeted data, the responses are the densities (/mm^2) of the total number of mutations, SBS, DBS, Indels, mutational type (missense, synonymous, splice, frameshift, or nonsense), positively selected mutant genes, and the percentage of epithelium mutant for the genes under positive selection.

We performed Kruskal-Wallis non-parametric tests for each factor and response pair, except for age, the only continuous variable, for which we used ANCOVA. For each analysis, multiple test correction was performed using the Benjamini-Hochberg method and p-values adjusted accordingly.

For the Nanoseq data, we found a significant association between treatment type and indel burden (Indel/Mb), and a borderline significant association of treatment and substitution burden (SBS/Mb) (Table 2).

For targeted data, we found a significant effect of treatment on the percentage of epithelia mutated for *TP53*, *PPM1D*, and *SPOP* (Table 2).

We conclude that the only significant effect on responses is from treatment and that other factors do not confound our analysis.

P_response	ME_treatment	ME_Sex	ME_Smoke	ME_Tumourtype	ME_Tumourlocation	ME_Age
**Nanoseq**						
p_Indel	0.0003	0.9295	1	0.9295	1	0.9295
p_Sub	0.0734	0.3705	0.3705	0.3705	0.7072	0.0734
p_SBS288A	0.905	0.5431	0.8518	0.905	0.905	0.905
p_SBS288B	0.1163	0.7007	0.4527	0.7007	0.329	0.318
**Targeted data**						
p_Mutations	1	1	1	1	0.396	1
p_SBS	0.915	0.915	0.915	1	0.915	0.915
p_DBS	0.25	0.988	0.988	0.723	0.723	0.09
p_Indel	1	1	1	1	0.08	1
p_Nonsyn	1	1	1	1	0.396	1
p_Missense	1	1	1	1	1	1
p_Frameshift	1	1	1	1	0.08	1
p_Splice	0.796	0.796	0.876	0.876	0.796	0.876
p_Nonsense	0.31	0.261	0.785	0.261	0.399	0.387
Percentage mutant epithelia						
p_NOTCH1	0.93	0.93	0.344	0.93	0.93	0.344
p_TP53	0.05	1	1	0.93	0.276	1
p_PPM1D	0.01	0.757	0.32	0.301	0.757	0.757
p_MTOR	0.221	0.782	0.221	0.171	0.761	0.299
p_NFE2L2	0.926	1	1	1	1	1
p_SPOP	0.044	0.806	1	1	0.1	1
p_RAC1	0.386	1	1	1	1	1
p_HLAB	0.166	0.875	0.48	0.639	0.921	0.76
Mutation density						
p_NOTCH1	0.531	0.907	0.907	0.268	0.531	0.952
p_TP53	0.379	1	1	1	0.349	0.379
p_PPM1D	1	1	1	0.602	1	1
p_MTOR	1	1	1	1	1	1
p_NFE2L2	1	1	1	1	1	1
p_SPOP	0.099	0.708	0.784	0.708	0.117	0.708
p_RAC1	0.44	1	1	1	1	1
p_HLAB	0.099	1	0.532	0.852	1	1

Table 2. Main effects of Treatment and other factors (association of responses and factors). Multiple test corrected p-values of non-parametric Kruskal Wallis are shown, except for age where ANCOVA was used. Effects where corrected p ≤ 0.05 are highlighted in blue.

Next, we investigated which treatments were responsible for the significant associations between treatment and factors using post hoc tests to compare each treatment with the NT group (Kruskal Wallis and Dunn's multiple comparison test). This showed CROSS patients had a higher burden of SBS and Indels as measured by Nanoseq (**Main text, Fig. 1d, 1f**) and a higher proportion of epithelium mutant for *TP53*, *PPM1D*, and *SPOP* (**Main text, Fig. 2b, 2c**).

2.2 Interaction effects

A lack of association with a main factor does not imply that there will be no significant association with the interaction of factors[6]. We therefore investigated if there is evidence of sensitivity in subcategories, for example if treatment affects males and females differently, by computing the significance of treatment and other factors' interaction effects on responses. We found no significant interactions between treatment and other factors using ANCOVA for age and ART ANOVA for all other factors[7] (**Table 3**).

p_response	inT_Sex	inT_smoking	inT_Tumour location	inT_Age
**Nanoseq**				
p_ints_Indel	1	1	1	1
p_ints_Sub	1	1	1	1
p_ints_SBS288A	0.958	1	1	1
p_ints_SBS288B	0.39	0.814	0.648	0.414
**Targeted data**				
p_ints_mutations	0.1921	1	1	1
p_ints_SBS	0.0684	1	1	1
p_ints_DBS	0.2095	0.733	0.2498	0.4164
p_ints_Indel	1	1	1	1
p_ints_Nonsyn	0.189	1	0.8158	1
p_ints_Missense	0.135	1	1	1
p_ints_Frameshift	1	1	1	1
p_ints_Splice	0.0922	0.3377	0.3377	0.595
p_ints_Nonsense	0.1958	0.9018	0.1958	0.4999
Percentage mutant epithelia				
p_ints_NOTCH1	1	1	1	1
p_ints_TP53	1	1	1	1
p_ints_PPM1D	0.2042	0.4497	0.4497	0.9158
p_ints_MTOR	0.1402	0.4033	0.1402	0.2761
p_ints_NFE2L2	0.4823	0.5722	0.7381	0.7895
p_ints_SPOP	1	1	1	0.3534
p_ints_RAC1	0.8072	0.8072	0.6228	0.8072
p_ints_HLAB	0.6099	0.3088	0.3088	0.3088
Mutation density				
p_ints_NOTCH1	0.43	0.06	0.43	0.965
p_ints_TP53	0.807	1	1	1
p_ints_PPM1D	0.956	0.956	0.203	0.984
p_ints_MTOR	0.162	0.696	0.483	0.693
p_ints_NFE2L2	0.649	0.699	0.699	0.983
p_ints_SPOP	0.177	0.177	0.945	0.945
p_ints_RAC1	1	1	1	1
p_ints_HLAB	1	1	1	1

Table 3. Interaction effect of Treatment and other factors. P-values from ANCOVA (age) or ART ANOVA (other factors) with Benjamini Hochberg multiple test correction are shown.

2.3 Potential confounders of missense mutation clustering results

We next investigated if factors other than treatment confounded that mutation clustering analysis. We used a Fisher exact test on the association of clustering domains with possible confounders for the three genes showing clustering associated with a treatment group (SPOP, NFE2L2 and MTOR). There were no significant associations with sex, tumor type, smoking status or tumour location. Therefore, they are not confounders (**Table 4**).

	Smoking	Sex	Tumour type (Adeno/SCC)	Tumour location
SPOP MATH vs other (CROSS group)	1	N/A, all males	1	1
NFE2L2 KEAP1 binding vs other (FLOT group)	0.27	0.19	N/A, all Adeno	N/A, all cardias
MTOR Kinase vs other (FLOT group)	0.51	1	N/A, all Adeno	N/A all cardias

Table 4. Possible confounders of 1D clustering results. P-values from Fisher exact test with Bonferroni multiple test correction are shown, N/A not applicable.

Supplementary Note References

1. Doench JG*, et al.* Optimized sgRNA design to maximize activity and minimize off-target effects of CRISPR-Cas9. *Nat Biotechnol* **34**, 184-191 (2016).

2. Labun K, Montague TG, Krause M, Torres Cleuren YN, Tjeldnes H, Valen E. CHOPCHOP v3: expanding the CRISPR web toolbox beyond genome editing. *Nucleic Acids Research* **47**, W171-W174 (2019).

3. Herms A*, et al.* Self-sustaining long-term 3D epithelioid cultures reveal drivers of clonal expansion in esophageal epithelium. *Nature Genetics* **56**, 2158-2173 (2024).

4. Tzelepis K*, et al.* A CRISPR Dropout Screen Identifies Genetic Vulnerabilities and Therapeutic Targets in Acute Myeloid Leukemia. *Cell Rep* **17**, 1193-1205 (2016).

5. Li W*, et al.* MAGeCK enables robust identification of essential genes from genome-scale CRISPR/Cas9 knockout screens. *Genome Biology* **15**, 554 (2014).

6. Szklo M, Nieto FJ. *Epidemiology: Beyond the basics*, 3 edn. Jones & Bartlett Learning, LLC (2012).

7. Wobbrock JO, Findlater L, Gergle D, Higgins JJ. The aligned rank transform for nonparametric factorial analyses using only anova procedures. In: *Proceedings of the SIGCHI Conference on Human Factors in Computing Systems*). Association for Computing Machinery (2011).
