# Supplementary material for: Cancer treatment alters mutant selection in normal esophagus
Source: Nat Genet. 2026 Sep 11;58(10):2566–78. doi: 10.1038/s41588-026-02738-0 (PMC13649571; doi:10.1038/s41588-026-02738-0)
Supplement: Supplementary file 2 [file 41588_2026_2738_MOESM2_ESM.pdf]

nature portfolio

Corresponding author(s): Philip Jones

Last updated by author(s): Jul 23, 2026

Reporting Summary

Nature Portfolio wishes to improve the reproducibility of the work that we publish. This form provides structure for consistency and transparency in reporting. For further information on Nature Portfolio policies, see our Editorial Policies and the Editorial Policy Checklist.

Statistics

For all statistical analyses, confirm that the following items are present in the figure legend, table legend, main text, or Methods section.

n/a	Confirmed	
☐	☒	The exact sample size (*n*) for each experimental group/condition, given as a discrete number and unit of measurement
☐	☒	A statement on whether measurements were taken from distinct samples or whether the same sample was measured repeatedly
☐	☒	The statistical test(s) used AND whether they are one- or two-sided<br>*Only common tests should be described solely by name; describe more complex techniques in the Methods section.*
☐	☒	A description of all covariates tested
☐	☒	A description of any assumptions or corrections, such as tests of normality and adjustment for multiple comparisons
☐	☒	A full description of the statistical parameters including central tendency (e.g. means) or other basic estimates (e.g. regression coefficient) AND variation (e.g. standard deviation) or associated estimates of uncertainty (e.g. confidence intervals)
☐	☒	For null hypothesis testing, the test statistic (e.g. *F*, *t*, *r*) with confidence intervals, effect sizes, degrees of freedom and *P* value noted<br>*Give P values as exact values whenever suitable.*
☒	☐	For Bayesian analysis, information on the choice of priors and Markov chain Monte Carlo settings
☒	☐	For hierarchical and complex designs, identification of the appropriate level for tests and full reporting of outcomes
☐	☒	Estimates of effect sizes (e.g. Cohen's *d*, Pearson's *r*), indicating how they were calculated

Our web collection on statistics for biologists contains articles on many of the points above.

Software and code

Policy information about availability of computer code

Data collection	Confocal images were obtained using the Leica Adquisiton Software LAS X

Data analysis	Targeted sequencing: BAM files were mapped to the GRCh37d5 reference genome using BWA-mem (version 0.7.17) . Duplicate reads were marked using SAMtools (v1.11). Depth of coverage was also calculated using SAMTools to exclude reads which were: unmapped, not in the primary alignment, failing platform/vendor quality checks or were PCR/Optical duplicates. BEDTools (version 2.23.0) coverage program was then used to calculate the depth of coverage per base across samples . For targeted sequencing data sub-clonal mutation variant calling was made using the deepSNV R package (also commonly referred to as ShearwaterML), version 1.21.3, available at https://github.com/gerstung-lab/deepSNV, used in conjunction with R version 3.3.0 (2016-05-03) 5. Whole genome sequencing: Variants were called using the CaVEMan (v.1.15.1) and Pindel (v3.7.0) algorithms 11 12. For SNVs CaVEMan was run with the major copy number set to 10 and the minor copy number set to 2. Nanoseq: For both SNV and indels only calls which passed all the defined filters were used https://github.com/cancerit/NanoSeq. Mutational spectra and signatures are described using the notation employed by the PCAWG Mutational Signatures working group 15. COSMIC signature definitions (v3.2) (https://cancer.sanger.ac.uk/signatures/sbs/) were used for Single Base Substitutions (SBS), Double base Substitutions (DBS) and Indel signature classification. Using SigProfiler packages MatrixGenerator (v1.2.12), Extractor (v1.1.12), Assignment (v0.0.13), Plotting (1.2.2). Statistical tests of mutation enrichment were applied using the python library Darwinian_shift20, available at https://github.com/michaelhall28/darwinian_shift. Code for calculating binding energies is available at https://github.com/shorthouse-lab/binding_ddg. Molecular dynamics simulations were performed using Gromacs version 2023.325. Analysis was also performed using mdanalysis (https://www.mdanalysis.org). All code used to perform analysis of selection, along with notebooks containing the figures generated from the raw data is available at https://github.com/shorthouse-lab/fowler_selection. Code for the simulation of clonal dynamics is available at https://github.com/hallba/King2026 [github.com] . Code for CRISPR/CAS9 analysis is available at https://github.com/PHJonesGroup/Fowler_etal_code , https://zenodo.org/records/21495557, DOI 10.5281/zenodo.21495556.

For manuscripts utilizing custom algorithms or software that are central to the research but not yet described in published literature, software must be made available to editors and reviewers. We strongly encourage code deposition in a community repository (e.g. GitHub). See the Nature Portfolio guidelines for submitting code & software for further information.

Data

Policy information about availability of data

All manuscripts must include a data availability statement. This statement should provide the following information, where applicable:

- Accession codes, unique identifiers, or web links for publicly available datasets
- A description of any restrictions on data availability
- For clinical datasets or third party data, please ensure that the statement adheres to our policy

The sequencing datasets generated during study will be available at the European Genome-phemone Archive (EGA) upon publication with the following dataset accession numbers:
EGAD00001011181 (WGS)
EGAD00001011182 (Targeted sequencing from 0.25mm punches)
EGAD00001011183 (Nanoseq)
EGAD00001011184 (Targeted sequencing from 2mm2 grids)

To access data email datasharing@sanger.ac.uk .

Research involving human participants, their data, or biological material

Policy information about studies with human participants or human data. See also policy information about sex, gender (identity/presentation), and sexual orientation and race, ethnicity and racism.

Reporting on sex and gender	Sex of all participants is specified in demographics table.
Reporting on race, ethnicity, or other socially relevant groupings	Race/Ethnicty was not recorded.
Population characteristics	Population characteristics are presented in the Demographics table.
Recruitment	All eligible patients were offered the study, those who consented were recruited.
Ethics oversight	Informed consent for the use of tissue was obtained in all cases. Ethical approval for patients from Milan was under study protocol ESO-CA001, approved by the OSR Institutional Ethics Committee. Ethical approval for patients from Southampton was under LREC number: 09/H0504/66, approved by the Southampton and South West Hampshire Research Ethics Committee, UK. Sequencing was performed at the Wellcome Trust Sanger Institute, Hinxton, UK under ethical approval 15/EE/0218 from the East of England - Cambridge East Research Ethics Committee.

Note that full information on the approval of the study protocol must also be provided in the manuscript.

Field-specific reporting

Please select the one below that is the best fit for your research. If you are not sure, read the appropriate sections before making your selection.

☒ Life sciences ☐ Behavioural & social sciences ☐ Ecological, evolutionary & environmental sciences

Life sciences study design

All studies must disclose on these points even when the disclosure is negative.

Sample size	This was a pilot study, so effect sizes were unknown.
Data exclusions	Copy number analysis was infeasible in one patient due to sparsity of heterozygous SNPs.
Replication	Each patient is an experimental unit. All groups achieved 7 or more replicates. Findings were consistent within each group.
Randomization	All eligible patients were included and analysed, there was no randomization in the study design.
Blinding	No blinding was used, investigators were aware of the treatment received by each patient.

Reporting for specific materials, systems and methods

We require information from authors about some types of materials, experimental systems and methods used in many studies. Here, indicate whether each material, system or method listed is relevant to your study. If you are not sure if a list item applies to your research, read the appropriate section before selecting a response.

Materials & experimental systems

n/a	Involved in the study	
☐	☒	Antibodies
☒	☐	Eukaryotic cell lines
☒	☐	Palaeontology and archaeology
☒	☐	Animals and other organisms
☒	☐	Clinical data
☒	☐	Dual use research of concern
☒	☐	Plants

Methods

n/a	Involved in the study	
☒	☐	ChIP-seq
☒	☐	Flow cytometry
☒	☐	MRI-based neuroimaging

Antibodies

Antibodies used	Ki67 ab15580, Abcam
Validation	Absent staining after Ki67 deletion in HAP1 cells, Abcam Website

Plants

Seed stocks	*Report on the source of all seed stocks or other plant material used. If applicable, state the seed stock centre and catalogue number. If plant specimens were collected from the field, describe the collection location, date and sampling procedures.*
Novel plant genotypes	*Describe the methods by which all novel plant genotypes were produced. This includes those generated by transgenic approaches, gene editing, chemical/radiation-based mutagenesis and hybridization. For transgenic lines, describe the transformation method, the number of independent lines analyzed and the generation upon which experiments were performed. For gene-edited lines, describe the editor used, the endogenous sequence targeted for editing, the targeting guide RNA sequence (if applicable) and how the editor was applied.*
Authentication	*Describe any authentication procedures for each seed stock used or novel genotype generated. Describe any experiments used to assess the effect of a mutation and, where applicable, how potential secondary effects (e.g. second site T-DNA insertions, mosiacism, off-target gene editing) were examined.*
