# Supplementary material for: Cancer treatment alters mutant selection in normal esophagus
Source: Nat Genet. 2026 Sep 11;58(10):2566–78. doi: 10.1038/s41588-026-02738-0 (PMC13649571; doi:10.1038/s41588-026-02738-0)
Supplement: Supplementary file 3 [file 41588_2026_2738_MOESM3_ESM.pdf]

nature portfolio

Peer Review File

Cancer treatment alters mutant selection in normal esophagus

Corresponding Author: Professor Philip Jones

Version 0:

Decision Letter:

5th Jan 2026

Dear Professor Jones,

Your Article, "Cancer treatment selects resistant mutants in normal esophagus" has now been seen by 3 referees. You will see from their comments copied below that while they find your work of considerable potential interest, they have raised quite substantial concerns that must be addressed. In light of these comments, we cannot accept the manuscript for publication, but would be very interested in considering a revised version that addresses these serious concerns.

We hope you will find the referees' comments useful as you decide how to proceed. If you wish to submit a substantially revised manuscript, please bear in mind that we will be reluctant to approach the referees again in the absence of major revisions.

If you choose to revise your manuscript taking into account all reviewer and editor comments, please highlight all changes in the manuscript text file. At this stage we will need you to upload a copy of the manuscript in MS Word .docx or similar editable format.

We are committed to providing a fair and constructive peer-review process. Do not hesitate to contact us if there are specific requests from the reviewers that you believe are technically impossible or unlikely to yield a meaningful outcome.

If revising your manuscript:

*1) Include a “Response to referees” document detailing, point-by-point, how you addressed each referee comment. If no action was taken to address a point, you must provide a compelling argument. This response will be sent back to the referees along with the revised manuscript.

*2) If you have not done so already please begin to revise your manuscript so that it conforms to our Article format instructions, available here.
Refer also to any guidelines provided in this letter.

*3) Include a revised version of any required Reporting Summary: https://www.nature.com/documents/nr-reporting-summary.pdf
It will be available to referees (and, potentially, statisticians) to aid in their evaluation if the manuscript goes back for peer review.
A revised checklist is essential for re-review of the paper.

Please be aware of our guidelines on digital image standards.

EXTENDED DATA FIGURES

When re-submitting your manuscript, please ensure that any supplementary figures and tables that are crucial to the manuscript’s conclusions are converted into Extended Data figures and tables to increase visibility of these data. Extended Data figures and tables are online-only (present in the online PDF and full-text HTML versions of the paper), peer-reviewed display items that provide essential background to the article but are not included in the main article due to space constraints. A maximum of ten Extended Data display items (figures and tables) is permitted.

You may use the link below to submit your revised manuscript and related files:

Link Redacted

Note: This URL links to your confidential home page and associated information about manuscripts you may have submitted, or that you are reviewing for us. If you wish to forward this email to co-authors, please delete the link to your homepage.

If you wish to submit a suitably revised manuscript we would hope to receive it within 6 months. If you cannot send it within this time, please let us know. We will be happy to consider your revision so long as nothing similar has been accepted for publication at Nature Genetics or published elsewhere. Should your manuscript be substantially delayed without notifying us in advance and your article is eventually published, the received date would be that of the revised, not the original, version.

Please do not hesitate to contact me if you have any questions or would like to discuss the required revisions further.

Nature Genetics is committed to improving transparency in authorship. As part of our efforts in this direction, we are now requesting that all authors identified as ‘corresponding author’ on published papers create and link their Open Researcher and Contributor Identifier (ORCID) with their account on the Manuscript Tracking System (MTS), prior to acceptance. ORCID helps the scientific community achieve unambiguous attribution of all scholarly contributions. You can create and link your ORCID from the home page of the MTS by clicking on ‘Modify my Springer Nature account’. For more information please visit please visit www.springernature.com/orcid.

Thank you for the opportunity to review your work.

Sincerely,

Safia Danovi, PhD
Senior Editor, Nature Genetics
ORCiD: 0009-0007-7822-5479

Referee expertise:

Referee #1: cancer genomics

Referee #2: somatic evolution

Referee #3: structural modelling

Reviewers' Comments:

Reviewer #1 (Remarks to the Author):

The manuscript by Fowler et al. studies a cohort of histopathologically normal esophageal epithelia sampled from patients with esophageal cancer who underwent esophagectomy after different standard of care cancer treatments (including patients not previously treated). The main question addressed in the study is whether and how the distinct treatments modify the clonal landscape found in normal epithelium.

To answer this question the authors sequenced the esophageal samples, deep sequencing tissue patches in a grided array against a custom panel of genes (>500x) and whole genome sequencing smaller punch samples. Upon variant calling, they studied the mutagenesis landscape as well as different surrogates of the tissue clonal composition, including dN/dS, the proportion of covered epithelium by mutant clones and a few other signals of positive selection via enrichment tests.
The question that the study addresses is pertinent and timely in view of the ongoing efforts in the field of somatic evolution to understand the effect of cancer therapies on different somatic tissues. The design and general approach of the study is sound, owing to established workflows that have been put forth by the authors and others in previous studies. The resulting data is valuable and the manuscript is well written.

In my view there are three important contributions of the study:
- Combined evidence of clonal composition differences between untreated and treated samples, comparable mutation burden, with a reported activity of chemotherapy-related mutational signatures which seems to be negligible in treated samples, suggesting a selective expansion of preexisting mutant clones. The absence of mutational footprints specific to chemotherapy is remarkable, suggesting a widespread removal of cells exposed to the genotoxic damage induced by chemotherapy.
- Evidence of relative expansion of certain clones specific to the type of treatment. For example, the relative expansion of TP53 and PPM1D mutant clones in response to chemo-radiation shows a very clear effect. Furthermore, the authors further validate PPM1D exon 6 mutants in esophageal epithelioid cultures exposed to radiation. Although this might not come as a complete surprise, this is a novel result in normal esophagus.

- Statistical analyses and modeling pointing towards the mechanism of action induced by the mutations reported under differential selection as well as the potential for druggability.

Although this reviewer acknowledges the value and importance of the study, there are two fundamental concerns: 1) the statistical association study drawing differences by treatment is not robust; 2) the framing of the differentially selected clones in normal esophagus as “resistant” would require further discussion and justification.

Next, I will articulate these concerns in the form of specific questions and comments:

Major issues
=========
- The statistical analyses from which the differential somatic evolutionary patterns are drawn are oblivious of confounding factors of somatic evolution described in other somatic tissues, such as age, alcohol consumption, smoking and sex. Beyond the questions about whether significance can hold while adjusting for these covariates, the study misses what in this reviewer’s view is an interesting question by itself, namely, whether there are any discernible effects which may be statistically attributable to either of these factors?

- The only five SCC cases in the cohort belong to the CROSS group. Is there any specific features of these samples clonal composition wise? Are there any clonal structure differences when accounting for different anatomical sites? In sum, are there any chances that these samples are biasing the results reported for CROSS?

- The authors do not provide a minimum correlation study that informs which covariates to include as potential confounding factors. Given the effect sizes that the authors report, it seems plausible that most results will continue to hold after adjusting for covariates, whichever are relevant in the light of a the correlation analysis. But this should be provided as evidence that there is no confusion due to the cohort composition.

- Despite the relevance of the absence of chemotherapy-induced mutagenesis to draw the conclusion that the relatively expanded clones were preexisting, the manuscript does not provide a concrete description of the signature deconstruction approach followed. Did the authors extract the signatures de novo? Did they refit with a predefined compendium of signatures learned from esophageal tumors? In the methods section only a generic paragraph enumerating the tools is provided, which in my opinion is not sufficient to cast reliability on the result.

- The above mentioned combined evidence of clonal differences, comparable mutation burden and absence of activity of chemotherapy-related mutational signatures, suggests the selective expansion of preexisting mutant clones. Are there any clear signals of negative selection gene wise? The authors mention briefly in the discussion the case of PTEN mutant clones, which seem to be positively selected in normal epithelium, although this effect seems to be lost upon treatment. I also wonder whether this can be a numerical artifact due to the low number of samples in the treatment groups compared to the non-treated.

- The use of resistance as leitmotif throughout the paper and the conceptual link with treatment resistance in tumors renders, in my opinion, a somewhat confusing framing of the results. The study addresses somatic evolution in the normal esophagus after exposure to cancer therapy, a system with evolutionary constraints that differ from those of a tumor. The authors posit that from the study of clonal dynamics in the normal esophagus a catalog of potential targets implicated in cancer therapy resistance can be learned. The reviewer agrees with this view. But the authors must make sure that they draw a very clean distinction between the normal and tumor evolutionary systems. In my opinion, the possibility possibility of finding false candidates should be explicitly discussed, as well as presenting what are the new potential targets that the study contributes.

Minor issues
=========

- The method 1D NMC is not described in the Methods section (but it is referenced in l. 353)

- How did the authors correct for uneven sequencing depth per site and sample when computing mutagenesis, dN/dS and other surrogates of selection and compared them?

- Supp. Fig. 5b-d, Supp. Fig. 6, what happened with the chromosome lengths?

- l. 361-362: Should this be pointing to the Methods section?

- l. 366-367: Non-treated show significantly increased number of mutations within the hinge residues. Increase with respect to what? Significantly higher than expected by neutral mutagenesis?

- l. 717: What does "statistics related to mutational selection" mean? Could the authors be more specific?

- Paragraph starting at l. 722: could the authors succinctly explain here how is the so-called 3D protein structure enrichment computed?

- Same paragraph: written “signature” where it should be read “profile”

- l. 50-52: The authors state “we hypothesized that anticancer treatment may selectively expand mutant clones that confer resistance to treatment in the densely mutated normal esophagus”. It might seem that what is implied is that the clonal status in the normal esophagus is somehow inducing the therapy response of the tumors adjacent in the same organ. I do not think this is what the authors intended to imply, although it leads to an interesting question, namely, whether there is any causal link between the evolutionary bottlenecks in the normal esophagus upon cancer treatment and the response to treatment of an hypothetical tumor arising in the same esophagus. This might well be true, but the current study design does not allow to make claims of this kind.

Reviewer #2 (Remarks to the Author):

I greatly enjoyed the manuscript by Fowler et al., and while there are interesting assertions worthy of publication at this level, the work falls short of being suitable at this time because of fundamental analytical shortcomings. I would support acceptance on resubmission, should the major issues that I raise be addressed and no subsequent issues arise.
======================================

Summary
Originality and significance: The work is novel and will be of general interest
Data & methodology: The data are generally of high quality, though biases in sampling are not clear
Appropriate use of statistics and treatment of uncertainties: The statistics driving many statements are occasionally superficial, lacking the appropriate rigor for suitable assertion of their conclusions. Conclusions:
Alternative explanations addressing potential sources of uncertainty are not applied readily. Interpretation of these results and potential future gaps in our knowledge would benefit from these being stated. Though their findings make theoretical sense, the inappropriate analyses used to test their system weaken their arguments.
Suggested improvements: More rigorous analyses and application of better statistical inference. It’s not immediately obvious how the associated github repo fits with the results presented- cleaning that up would improve accessibility to the author’s conclusions and methods.
References: The authors should check for previous evidence for some of their major findings- that there is evidence for chemotherapy in sequencing data and that PPM1D confers radio-resistance.
Clarity and context: For the most part the manuscript is extremely well written and clear.

Theory states that populations will face selective pressure from strong, persistent environmental perturbation. It stands to reason that somatic tissue will undergo selection when faced with pressure from chemotherapeutic regimes.

Fowler et al. attempt to explore this through characterisation of a cohort of patients with esophageal cancer, for whom they are able to group by different therapeutic regimes, each involving exposure to DNA damaging agents. They biopsy normal tissue and dissect it into multiple gridded subunits, before using nanoseq to demonstrate treatment-specific mutational patterns across ~30% of the genome. No mutations in these data are attributed to any of the chemotherapeutic exposures, therefore the authors reason that clones existed before treatment and that any difference in mutation rate between groups reflects selection for different mutants as a result of treatment regime. They subsequently apply targeted, high depth sequencing to many more gridded subunits to define signals of selection across a panel of 324 genes. A range of subsequent analyses are performed with the aim of exploring the functional consequences of mutations under selection.
======================================

Major points

1) Mutation signature analysis not robust enough

That none of the mutations from mutagenic exposure are found is surprising, given the range of evidence found using the same techniques across multiple tissue types already published and as partly alluded to in the text. There is no evidence of smoking, though many of the patients are, or were, smokers. Given the importance of this finding in driving the narrative, it is noteworthy that there are many discrepancies in the characterisation of mutational processes in this work.

The authors list use of Sigprofiler extractor, but there is no evidence that they performed de novo extraction of signatures. Figure 1e, which is a summary of the mutation signatures in these data, isn’t representative of the heterogeneity of signals across the cohort. For example, the signal from SBS16 represents only 3 samples in CROSS, and some of the samples have a surprising lack of diversity; 2 are 100% SBS40 and 16 have no SBS5.

In Lawson et al. 2025, where nanoseq was applied to a much greater depth, SBS5 is deconvoluted in a mixture along with SBS1 and presented as SBSA, for which not a single individual lacks signal- see figure 4a and c in https://www.nature.com/articles/s41586-025-09584-w. That Lawson et al. are unable to distinguish well-known and generally ubiquitous signatures suggests that de novo deconvolution is vital for recognising the active mutational processes in these data, as they may appear differently, or even as composites of previously documented signatures.

Looking across the rate of substitutions per Mb suggests that many of the mutations in these data are unattributed during signature fitting, e.g. PD47501 has a Sub/Mb of 0.59 and, when multiplied by 3200 Mb, suggests that there are likely 882 unattributed mutations, with 1006 attributed across the 4 signatures. This also suggests that the mutational signature fitting has not been adequately performed.

In order for this work to sufficiently address these shortcomings, the authors should provide evidence of de novo deconvolution and strive to find alternative signals for exposure in their data. For example, fitting of double base substitutions associated with platinum-based therapies and tobacco smoke would be useful. It is possible that mutational signatures among individual samples could form the bases for a cleaner analysis, e.g. further justification for the inclusion of the outlier in NT, and the two in CROSS.

The excess of non-exposure derived signatures between NT and treatment regimes would therefore be the measure of selection to move forward with. The presence of signatures from treatment or tobacco smoke doesn’t necessarily mean that treatment hasn’t caused selection for specific variants already present in the population, however it would need to be stated that the mutations found are possibly the result of enrichment through mutagenesis rather than selection. It would also need to be acknowledged that clock-like processes might be enriched in rapidly expanding populations under selection, though this could be addressed with additional analyses.

2) Confounding sources of variation throughout and superficial statistical analysis

There are many assertions throughout the manuscript that could be made more emphatically if they demonstrated that they weren't potentially an artifact of confounding variables, such as those in the metadata. An example of this is that all 5 of the SCC samples in these data are in the CROSS group (n=11) and is therefore a potentially confounding variable where the CROSS group is suggested to be under selection. More appropriate statistics, aiming to define the role of these effects and that I suggest subsequently, would make for stronger evidence supporting the author’s narrative.

To emphasise this point, the author’s own analyses of smokers vs. non-smokers (1a, https://www.biorxiv.org/content/10.1101/2024.09.26.615123v1) suggests that this will lead to an increase in mutation load). Though there doesn’t appear to be a bias for smokers across exposure groups (Fisher’s exact test), it is clear that their analyses obfuscate the potential role of this mutational process in these data. While it is important for many aspects relating to quantification of mutational processes, controlling for sample representation will also be important where their representation is uneven, e.g. calculation of clone size; how do I know that the increase in TP53 clone size in CROSS (Fig 2d) isn't driven by a single patient or samples with inadequate sequencing? I especially want it to be clear if the signals for selection, i.e. differences in mutation rate, dn/ds and results in Fig S7 for SPOP, are actually driven by SCC, tissue location or tobacco smoke.

To ensure that the author’s statements are robust to the potential impact of confounding variables, I would hope to see some attempts to pull apart the data based upon these variables, such as multivariate regression. For example, this could be applied to the data in Fig S2c and d, where none of the variability between samples and groups is explored with statistical rigor, but is used to state that there is no difference between mutation type in each group.

Similar analyses for individual genes is problematic in its own right, such as is shown in Fig 3C. It is important to bear in mind the inherent biases associated with gene length and codon use. An example is that 43% of the possible mutations over exons 5-8 of TP53 are missense (https://www.nature.com/articles/s41588-024-02039-4#MOESM4). The authors could instead calculate the log of odds from fishers exact test on each patient and compare against the same gene of bootstrapped mutations randomly sampled from all patients in NT.

For these data, where clonal inference is so important, it is striking that mutational processes from nanoseq are assumed to be equally relevant in the panel sequencing data. I acknowledge that many of these measures will be biased in these data, but the authors should attempt to demonstrate that the clones analysed here are under the same selective pressure through by estimating the mutation rate at synonymous sites as a proxy for the neutral mutation rate (presented in Fig S3d), to show that it correlates with your previous nanoseq data. Mutation signatures would also emphasise that the data are well matched.

In terms of strengthening the inference of these analyses, especially where smoking is so relevant, I find it hard to understand why the “UK normal” data weren’t included wherever else they’re applicable. This would be really useful to show that smoking has no impact on your analyses.

3) Inclusion of FLOT and in vitro experiment

As it stands, there's no evidence for therapy-associated clonal outgrowth for FLOT in the same way as the evidence suggests for CROSS. It doesn’t fit with the narrative of the work, building that excess mutations from nanoseq signify selection and then disregarding this evidence based upon unique, high dn/ds.

It would make for a nice segue into the section if you could create the same analyses from Fig S4D for NFE2L2, which is uniquely under selection under this regime and relevant to 5FU. A statement about the potential differences in strength of selection or the fact that these clones might need more time than those under CROSS to rise in number and be detectable in your nanoseq analyses would help here.

It’s worth mentioning here that the in vitro work from L226 is a nice, complementary experiment, but doesn't tell us if it's actually the combined effect of carboplatin, paclitaxel and radiation in combination as is assessed in vivo. The best you can say without running more explicit experiments is that PPM1D confers resistance to radiotherapy in vitro. for which there appears to be some literature already. There are no statistics for Fig S4D, when there should be.
====================================

Minor points

Fig S3B and C looks like the percentage of mutant epithelia decreases with age, as does its variance. That super interesting and it wouldn't take much to formally test this. Fig S1d is also of interest and it would be useful to show that slopes the same for each exposure group, if true.

The authors cite Mitchell et al. 2025 (64) to show that signatures for chemotherapy are found in duplex sequencing. I would add other work by Nuria Lopez Bigas, Sam Behjati and Jyoti Nangalia, who have all demonstrated that similar signals are prevalent and to drive that these findings are unusual.

Fig S2a Is there no cut off for poorly/excessively sequenced samples? If there is QC for inclusion, please state any thresholds or tests.

L258 How were these samples selected? Sample numbers are very biased towards CROSS samples, so it would be good to know upfront the sample numbers. It's a shame that the FLOT and NT samples originate from only two patients, but it should be stated clearly.
=====================================

Formatting etc

Fig 3e In the block for sample 3, there are two unjoined groups of squares- what does that represent?

L89 I don't think these papers need to be cited with respect to a statement about your own results.

L261 Please include a reference for "Loss of heterozygosity (LOH) of the NOTCH1 gene were seen as expected"

L167 This sentence is redundant and should be removed.

Table s3 sample PD59796 is missing

L59 It isn't obvious that the combinations of chemotherapeutics associated with each regime is detailed in Table S1 and their administration in Fig 1b. The description of the treatments also isn't exactly representative of FLOT, which is a platinum agent, a taxane AND an antimetabolite with folonic acid. The description of drug combinations. Could be expressly written in full on 1b and make up for the white space of 1a, which could be stacked.

L641 a non synonymous mutations with VAF>0.35 // one of these is wrong: XaX non or synonymous mutationXsX

L153 This is turn suggests that …

L67 “excised” d
=====================================

Reviewer #2 (Remarks on code availability):

Data availability and processing:

Data under EGA accessions are unavailable as of 11.12.25

I decided to follow the notebook in ATM, as this gene isn’t mentioned in the manuscript and not considered among any of the genes under selection.

Following their instructions, I installed a conda environment for the darwinian_shift package and downloaded the human genome reference data required from the earlier version of this package. I had to manually call the genome and annotation with the reference_fasta and exon_file options, where the authors called the source_genome option. I needed to install another dependency to open the external data: pip install openpyxl. I was able to run all the code and reproduce all the figures but the last, which was very different. It is not clear to me how this plot fits in with those of the rest of the manuscript- the plot is included in the attached PDF.

On code for data processing not included in the github, suitable github repositories are provided in the methods section.

On the github link provided for this work, there exist the following folders:
ATM - jupiter notebook as other genes
Foldx - nothing to run
Foldx_binding - nothing to run
MTOR - jupiter notebook as other genes
RAC1 - jupiter notebook as other genes
SPOP - jupiter notebook as other genes
clustering - nothing to run

Reviewer #3 (Remarks to the Author):

This manuscript examines how neoadjuvant chemotherapy and chemoradiation shape somatic clonal selection in histologically normal human oesophageal epithelium. By combining high-depth targeted sequencing with NanoSeq, the authors show that treatment does not substantially increase mutation burden but instead selectively expands pre-existing mutant clones in a treatment-specific manner. In particular, TP53 and PPM1D mutant clones are enriched following chemoradiation, while mutations in genes such as MTOR, RAC1, and NFE2L2 are preferentially selected in patients receiving FLOT chemotherapy.

My comments focus primarily on the protein- and structure-level interpretation of missense mutation selection, including the use of structural clustering, molecular modelling, and ΔΔG-based analyses to infer functional consequences of selected variants.

General methodological and reporting issues

• The manuscript refers to "genes for which structural information was available", but it is unclear how this was defined. Given the widespread availability of high-quality predicted structures, clarification is needed as to whether this refers specifically to experimentally determined PDB structures, ligand-bound conformations, or other criteria. This distinction is important for understanding the scope and potential biases of the 3D clustering and ΔΔG analyses.

• The 1D clustering analysis using NMC is reasonably well defined. However, the manuscript refers to "3D clustering" without clearly specifying a single metric or statistical framework. It would be helpful to clarify how 3D clustering is defined, including how distances are computed, what constitutes a cluster versus enrichment, and how significance is assessed and corrected for multiple testing. In addition, clustering is generally reported in binary terms (significant vs non-significant), which makes it difficult to compare the strength of clustering across genes and treatment groups. Providing quantitative measures of clustering or enrichment (for example, observed versus expected mutation density within predefined functional regions, or effect sizes with confidence intervals) would allow more direct comparison between conditions and help distinguish baseline selection from treatment-specific effects. As currently presented, multiple conceptually distinct analyses are grouped under the term "3D clustering", limiting assessment of robustness and reproducibility.

• The ΔΔG-binding analyses rely heavily on FoldX-based scoring. FoldX was originally developed for protein stability estimation and is not well validated for ligand binding, particularly for non-standard ligands. The manuscript would benefit from clarification of ligand parameterisation, benchmarking against known effects, and more cautious interpretation of binding energy changes.

• The dN/dS analyses appear to be performed across the full targeted gene panel, but only a small subset of genes are reported in the supplementary tables. Providing complete gene-level results (including non-significant genes), or at least ranked summaries per treatment group, would greatly aid interpretation and assessment of the specificity and relative strength of the reported selection signals.

Gene-specific interpretation of missense selection

-SPOP

• The interpretation of selected SPOP missense mutations as loss-of-function under chemoradiation would benefit from clearer definition and stronger calibration. While prior literature supports classification of prostate cancer SPOP mutations as loss-of-function, the manuscript does not show whether these reference mutations exhibit comparable 1D or 3D clustering patterns to the CROSS-selected variants. Demonstrating similar spatial clustering would strengthen the inference that the two sets share a common functional mechanism.

• In addition, the ΔΔG analyses focus exclusively on ligand-binding energetics. Loss of function in adaptor proteins such as SPOP is often mediated by effects on folding stability, abundance, or regulation rather than selective disruption of a single interaction. Assessing predicted stability effects, or clarifying why ligand binding is expected to be the dominant mechanism in this context, would help justify the functional interpretation.

• Finally, spatial clustering of missense mutations supports functional relevance but does not distinguish loss- from gain-of-function, as clustering has frequently been associated with activating mutations in cancer. It is also notable that significant clustering relative to the ligand-binding site is observed in the no-treatment group. This suggests that clustering reflects baseline selection on SPOP rather than a chemoradiation-specific effect, and therefore does not, by itself, support a treatment-specific loss-of-function mechanism. Notably, in Fig. 3g the distribution of predicted binding energy changes for CROSS-selected mutations appears closer to the neutral distribution than to the prostate cancer loss-of-function set, suggesting that effects may be modest or heterogeneous. A more cautious framing of the conclusions, or additional quantitative comparisons, would therefore be appropriate.

-NFE2L2

• The NFE2L2 analysis is biologically plausible and broadly consistent with prior work on KEAP1-NFE2L2 regulation. However, clustering of missense mutations within the KEAP1-binding region is also observed in ECX and no-treatment groups, indicating baseline selection on this interface. The treatment-specific effect therefore appears quantitative rather

than qualitative, and this distinction would benefit from clearer emphasis.

• In addition, the conclusion that selected NFE2L2 mutations are protein-stabilising and confer chemoresistance is inferred from positional clustering and prior literature rather than demonstrated directly in this system. Incorporating predicted ΔΔG effects on protein stability for the observed missense variants, or otherwise quantifying their likely impact on NFE2L2 stability or abundance, would help support the proposed mechanism and distinguish stabilising regulatory effects from alternative functional consequences. Clarifying this point, or moderating the language accordingly, would strengthen this section.

-RAC1
• The RAC1 analysis integrates clustering, structural proximity metrics, ΔΔG-binding predictions, and MD simulations to infer selection for activating mutations under FLOT. While the biological framework is plausible, several aspects would benefit from clarification. Notably, enrichment of mutations in hinge residues is observed in both FLOT and no-treatment groups, indicating baseline selection on RAC1 rather than a treatment-specific effect. The FLOT-specific conclusion therefore rests primarily on differences in proximity to GTP and predicted binding energetics, for which effect sizes and biological significance are not fully established.

• In addition, RAC1 functions as a molecular switch, and activating mutations need not act by increasing GTP affinity alone. Many oncogenic GTPase mutations alter conformational cycling, impair GAP-mediated hydrolysis, or modify effector interactions, often through effects on Switch I or Switch II regions. Clarifying whether the identified hinge mutations overlap these functionally critical regions, rather than relying primarily on proximity to bound GTP, would strengthen the mechanistic interpretation.

• The MD simulations are performed for a small, selected set of mutations, including known gain-of-function variants, and no comparison is shown for mutations observed in untreated samples. As presented, these simulations support plausibility but do not independently distinguish treatment-specific activating effects. A more cautious framing of the conclusions, or additional quantitative comparison across groups, would strengthen this section.

-MTOR
• The MTOR analysis is compelling and benefits from strong prior knowledge of kinase-domain gain-of-function mutations and their association with increased clonal fitness. The enrichment of kinase-domain mutations, their overlap with known activating variants, and their association with larger clone sizes together provide convincing support for selection of activating MTOR mutations, particularly under FLOT treatment.

• However, activation of MTOR can arise through allosteric relief of autoinhibition as well as direct effects on the kinase domain. Clarifying whether the selected mutations map to regions associated with allosteric regulation or structural unlocking would further strengthen the interpretation of these variants as activating, and may help explain why pathway activation is observed without predicted disruption of rapamycin binding.

• The subsequent interpretation regarding druggability and resistance would also benefit from clarification. Predicted lack of effect on rapamycin binding does not directly imply pathway sensitivity or resistance in vivo, and the conclusion that "druggable GoF MTOR mutants confer resistance" appears broader than what is supported by the presented data. A more cautious framing distinguishing selection under chemotherapy from therapeutic resistance, or clarification of the specific form of resistance being implied, would strengthen this section.

Version 1:

Decision Letter:

1st Jun 2026

Dear Professor Jones,

Your Article, "Cancer treatment alters mutant selection in normal esophagus" has now been seen by 3 referees. You will see from their comments below that while they find your work of interest, some important points are raised. We are interested in the possibility of publishing your study in Nature Genetics, but would like to consider your response to these concerns in the form of a revised manuscript before we make a final decision on publication.

We therefore invite you to revise your manuscript taking into account all reviewer and editor comments. Please highlight all changes in the manuscript text file. At this stage we will need you to upload a copy of the manuscript in MS Word .docx or similar editable format.

We are committed to providing a fair and constructive peer-review process. Do not hesitate to contact us if there are specific requests from the reviewers that you believe are technically impossible or unlikely to yield a meaningful outcome.

When revising your manuscript:

*1) Include a “Response to referees” document detailing, point-by-point, how you addressed each referee comment. If no

action was taken to address a point, you must provide a compelling argument. This response will be sent back to the referees along with the revised manuscript.

*2) If you have not done so already please begin to revise your manuscript so that it conforms to our Article format instructions, available
here.
Refer also to any guidelines provided in this letter.

*3) Include a revised version of any required Reporting Summary: https://www.nature.com/documents/nr-reporting-summary.pdf
It will be available to referees (and, potentially, statisticians) to aid in their evaluation if the manuscript goes back for peer review.
A revised checklist is essential for re-review of the paper.

Please be aware of our guidelines on digital image standards.

EXTENDED DATA FIGURES

When re-submitting your manuscript, please ensure that any supplementary figures and tables that are crucial to the manuscript's conclusions are converted into Extended Data figures and tables to increase visibility of these data. Extended Data figures and tables are online-only (present in the online PDF and full-text HTML versions of the paper), peer-reviewed display items that provide essential background to the article but are not included in the main article due to space constraints. A maximum of ten Extended Data display items (figures and tables) is permitted.

Please use the link below to submit your revised manuscript and related files:

Link Redacted

Note: This URL links to your confidential home page and associated information about manuscripts you may have submitted, or that you are reviewing for us. If you wish to forward this email to co-authors, please delete the link to your homepage.

We hope to receive your revised manuscript within four to eight weeks. If you cannot send it within this time, please let us know.

Please do not hesitate to contact me if you have any questions or would like to discuss these revisions further.

Nature Genetics is committed to improving transparency in authorship. As part of our efforts in this direction, we are now requesting that all authors identified as 'corresponding author' on published papers create and link their Open Researcher and Contributor Identifier (ORCID) with their account on the Manuscript Tracking System (MTS), prior to acceptance. ORCID helps the scientific community achieve unambiguous attribution of all scholarly contributions. You can create and link your ORCID from the home page of the MTS by clicking on 'Modify my Springer Nature account'. For more information please visit please visit www.springernature.com/orcid.

We look forward to seeing the revised manuscript and thank you for the opportunity to review your work.

Sincerely,

Safia Danovi, PhD
Senior Editor, Nature Genetics
ORCiD: 0009-0007-7822-5479

Reviewers' Comments:

Reviewer #1 (Remarks to the Author):

The authors have done a commendable effort to address the issues raised in the first review. As far as this reviewer is concerned, comprehensive, sufficient amount of clarification has been provided in response to the comments and questions by the panel of reviewers and in the text of the manuscript. In particular, the manuscript is supported by more robust statistical analyses, the reporting is comprehensive, the methodology better described, the mechanistic interpretation has been refined. All in all, the way the results are now framed makes more sense.

With all that said, there is a reminder comment regarding the very remarkable, almost puzzling result that there were no traces of mutational signatures of 5-FU, oxaliplatin or carboplatin (SBS25, 31 and 35) and only the CROSS samples witnessed a significant increase of the mutation burden. The authors postulate that this may reflect a selective loss of normal

oesophageal cells damaged by these agents. In the response to reviewers the authors also refer to the possibility that clonal expansions after the CROSS regime can explain more mutations being detected in the corresponding samples due to an increase in the proportion of mutated epithelium without requiring foreign mutational processes: according to this hypothesis, we would expect that the clock like signatures + SBS16 in specific cases are predominantly due to low chemo exposed cells, with the higher CROSS burden being a result of a mixture of expanded clock-like mutations and maybe foreign mutations specific to ionizing radiation. Another possible explanation may be that, even under completely neutral selection, some endogenous mutational signatures, most prominently SBS5, may funnel heterogeneous sources of DNA damage as postulated in this preprint (https://www.biorxiv.org/content/10.1101/2025.08.28.672844v2).

As much as these explanations can make sense, in view of the technical difficulties to completely rule out the chemotherapy mutational footprint – and also for the sake of establishing sensitivity bounds due to the methodology in place – this reviewer suggests, at the discretion of the authors, a few more stringent tests that could be conducted as part of this analysis.

How to be extra confident that there are no traces of chemotherapy induced mutations? What would be the sensitivity bounds owing to fitting method of choice?

This reviewer suggests two potential extra avenues to completely clarify this point, insofar as this is one of the most striking observations of the study.

- To check whether the de novo signature extraction of choice is in agreement with an alternative method. By experience of our group, this reviewer would suggest looking into the results of HDP (https://github.com/McGranahanLab/HDP_sigExtraction) or MSA (https://gitlab.com/s.senkin/MSA) to confirm the consistency of the mapping of the mutation catalogs with mutational processes.

- In order to be extra confident of the presence or absence of a footprint of a certain foreign signature, it might also be useful to conduct a likelihood test, whereby a collection of baseline signatures representing the typical mutational processes operative in the oesophagus can be used, e.g. SBS1, 5, 40a, 16, to test whether the reconstruction would significantly improve by including an additional query signature (in this case, treatment-associated) like SBS25, 31, 35. This can be accomplished by e.g. an off-the-shelf method like mSigAct (https://github.com/steverozen/mSigAct).

Reviewer #2 (Remarks to the Author):

The authors have responded quickly and clearly worked hard. I am very happy with their responses, and in particular am pleased to see supplementary note 1. I would be happy to see this accepted if the authors address two minor points that I'll raise in summary at the end.

I'll go through my thoughts on the author responses as I formatted my initial review and unless I refer to something specifically, it should be taken that I am satisfied that the authors have sufficiently addressed any issues.

Major point 1) Mutation signature analysis not robust enough

This is a much stronger analysis and feel that their efforts of mutation signature deconvolution are defensible. I am still surprised that there are no chemo or smoking signatures, but that’s the data and I accept the author’s arguments as to why this is. Their reason for not using DBS to try and infer exposure-based signatures is also very fair and the outcome of the simulations is acceptable, especially as there are no treatment-specific signature.

I like the inclusion of the SBS288 signatures and their fitting as supplementary figures. The authors very graciously handled my misunderstanding as to the extent of genome coverage of nanoseq and made a good attempt to link mutational processes therein with their panel-based sequencing. Though the outcome is not very satisfying, I am happy that to push for anything else is beyond the scope of their efforts.

Major point 2) Confounding sources of variation throughout and superficial statistical analysis

Reviewer figs 6 and 8 are not insightful and do not do enough to convey the impact of bias on the analyses through a lack of statistical analysis, though this does not impact the manuscript. In spite of this, supplementary note 1 does do a good job and I am happy that there are no confounding aspects of patient cohorts in their findings. To this point, I took the VAF data for TP53 SNVs from Table 5 and ran a mixed effects model that accounts for patient and sample as mixed effects to assess the impact of treatment, smoking, gender, disease state and location on VAF. My analysis agrees with the findings of the authors, that only the CROSS treatment type shows a significant effect on TP53 clone size ($p = 0.0349$).

The author’s use Kendall’s concordance test to define significant differences the proportion of types of mutations, but if I’m not mistaken, this uses ranks as input and therefore isn’t appropriate for this purpose.

In their attempt to compare neutral mutations between panel-based sequencing and nanoseq, I appreciate that the inherent bias in each of the methods makes it difficult to derive comparable mutational process. Despite this, the authors attempt mutation signature fitting of the panel-based data, and while broadly similar, there are a couple of points that stand out: SBS4 is present, with nonsmokers making up 2/7 with considerable sbs4 burden (Pd49362, Pd49363). The authors state that there are 5 patients with sbs4, but there are 7. There are inconsistencies here that don’t gel with the narrative in the

manuscript, but I appreciate that the comparison of the two data types and use of signature fitting on pull-downs of the genome is not necessarily a fair or very accurate way of comparing the two sequencing methods. I appreciate their effort and will leave it at that.

Major point 3) Inclusion of FLOT and in vitro experiment

I'm happy with the Author's responses. Given the level of detail that they go into with regard to Nfe2l2, it's fair to say that they have attempted this experiment and that I can't ask for more. The lack of this makes the narrative a bit nonsensical, but the results are sufficiently worthwhile that most readers won't be bothered by it.

Minor points:
I can see the EGA accessions now, though the github repository has not been updated despite the author's response. It says the last update was 13.07.25, therefore it was not rerun. Was an update pushed?

Summary:

I would like to see the github repository actually updated and a justification for, or alteration of, use of Kendall's concordance test.

Reviewer #2 (Remarks on code availability):

The github repository has not been updated despite the author's response. It says the last update was 13.07.25, therefore it was not rerun.

Reviewer #3 (Remarks to the Author):

The authors have responded constructively to my comments and have substantially improved the structural and protein-level interpretation. In particular, the revised manuscript now defines the structural datasets used, separates 1D clustering from structural enrichment analyses, provides clearer descriptions of the null models and statistical tests, expands the dN/dS reporting, and moderates several of the mechanistic conclusions. I am satisfied that the my main concerns have been addressed.

Version 2:

Decision Letter:

Our ref: NG-A70761R1

3rd Jul 2026

Dear Dr Jones,

Thank you for submitting your revised manuscript "Cancer treatment alters mutant selection in normal esophagus" (NG-A70761R1). It has now been seen by two of your original referees and their comments are below. The reviewers find that the paper has improved in revision, and therefore we'll be happy in principle to publish it in Nature Genetics, pending minor revisions to satisfy the referees' final requests and to comply with our editorial and formatting guidelines.

If the current version of your manuscript is in a PDF format, please email us a copy of the file in an editable format (Microsoft Word or LaTex)-- we can not proceed with PDFs at this stage.

We are now performing detailed checks on your paper and will send you a checklist detailing our editorial and formatting requirements soon. Please do not upload the final materials and make any revisions until you receive this additional information from us.

Thank you again for your interest in Nature Genetics Please do not hesitate to contact me if you have any questions.

Sincerely,

Safia Danovi, PhD
Senior Editor, Nature Genetics
ORCiD: 0009-0007-7822-5479

Reviewer #1 (Remarks to the Author):

I owe sincere thanks to the authors for answering what I assume will be the last round of Q&A. As far as this reviewer is concerned, the authors have fulfilled a relevant bundle of questions, enough to ensure a solid, compelling analysis.

In particular, thanks for providing the two signature analyses that this reviewer suggested. In view of the outputs, I would like to submit a couple of final remarks.

As much as it is slightly surprising, the output cast by MutationalPatterns highlights the limitations, conducive to the lack of reproducibility, of signature extraction methods when it comes to input data that does not meet the ideal conditions in terms of cohort size, homogeneity and balanced exposures. While I do not find the output of this analysis particularly compelling per se, it points towards the challenge that some datasets might represent for these methods and to what extent well-established de novo extraction methodologies fail to provide a reliable, comprehensive account of the footprints of operative mutational processes.

I found the outcome of the statistical tests with the mSigAct method more compelling. While it does not cast a clear conclusion either in terms of the biology, in this reviewer's view it provides a more systematic and intuitive grasp of what makes the signature analysis methodology so challenging in this particular cohort, suggesting that there might be a fundamental data limitation whereby not all operative signatures can be learned, particularly those that might come at low exposures or might be unevenly or sparsely operative across samples, both in treated and untreated samples. Last but not least, on account of the fact that it has been previously linked with 5FU treatment, I think signature SBS17b must also be included in this analysis. I would encourage the authors to submit this statistical analysis as a supplemental data item.

No more answers are requested nor expected in response to this final round of remarks.

Reviewer #2 (Remarks to the Author):

I'm satisfied with the author's response to my question about the statistical method used and I'm satisfied that the github has been updated and better represents the plots presented in the manuscript.

I've nothing else to add and am grateful to the authors for their hard work.

Reviewer #2 (Remarks on code availability):

I haven't run the code freshly, but I have checked that the github repo is updated, as requested. The output and figures in their Jupyter notebook match the analyses and plots in the manuscript.

INTERNAL USE ONLY AIP modules required: ["Clinical","Life Science"]

Open Access This Peer Review File is licensed under a Creative Commons Attribution 4.0 International License, which permits use, sharing, adaptation, distribution and reproduction in any medium or format, as long as you give appropriate credit to the original author(s) and the source, provide a link to the Creative Commons license, and indicate if changes were made.

In cases where reviewers are anonymous, credit should be given to 'Anonymous Referee' and the source.
The images or other third party material in this Peer Review File are included in the article's Creative Commons license, unless indicated otherwise in a credit line to the material. If material is not included in the article's Creative Commons license and your intended use is not permitted by statutory regulation or exceeds the permitted use, you will need to obtain permission directly from the copyright holder.
To view a copy of this license, visit https://creativecommons.org/licenses/by/4.0/

Response to reviewers, NG-A70761: Cancer treatment alters mutant selection in normal esophagus

We are most grateful to the reviewers for their careful critique of the manuscript. Their comments have substantially improved the revised text. In the below, our comments are in blue.

Reviewer #1 (Remarks to the Author):

The manuscript by Fowler et al. studies a cohort of histopathologically normal esophageal epithelia sampled from patients with esophageal cancer who underwent esophagectomy after different standard of care cancer treatments (including patients not previously treated). The main question addressed in the study is whether and how the distinct treatments modify the clonal landscape found in normal epithelium.

To answer this question the authors sequenced the esophageal samples, deep sequencing tissue patches in a grided array against a custom panel of genes (>500x) and whole genome sequencing smaller punch samples. Upon variant calling, they studied the mutagenesis landscape as well as different surrogates of the tissue clonal composition, including dN/dS, the proportion of covered epithelium by mutant clones and a few other signals of positive selection via enrichment tests.

The question that the study addresses is pertinent and timely in view of the ongoing efforts in the field of somatic evolution to understand the effect of cancer therapies on different somatic tissues. The design and general approach of the study is sound, owing to established workflows that have been put forth by the authors and others in previous studies. The resulting data is valuable and the manuscript is well written.

In my view there are three important contributions of the study:
- Combined evidence of clonal composition differences between untreated and treated samples, comparable mutation burden, with a reported activity of chemotherapy-related mutational signatures which seems to be negligible in treated samples, suggesting a selective expansion of preexisting mutant clones. The absence of mutational footprints specific to chemotherapy is remarkable, suggesting a widespread removal of cells exposed to the genotoxic damage induced by chemotherapy.
- Evidence of relative expansion of certain clones specific to the type of treatment. For example, the relative expansion of TP53 and PPM1D mutant clones in response to chemo-radiation shows a very clear effect. Furthermore, the authors further validate PPM1D exon 6 mutants in esophageal epithelioid cultures exposed to radiation. Although this might not come as a complete surprise, this is a novel result in normal esophagus.
- Statistical analyses and modeling pointing towards the mechanism of action induced by the mutations reported under differential selection as well as the potential for druggability.

We are most grateful to the reviewer for their critical reading of the text and their positive suggestions which have improved the revised manuscript.

Although this reviewer acknowledges the value and importance of the study, there are two fundamental concerns: 1) the statistical association study drawing differences by treatment is not robust; 2) the framing of the differentially selected clones in normal esophagus as “resistant” would require further discussion and justification.

Next, I will articulate these concerns in the form of specific questions and comments:

Major issues

=========

- The statistical analyses from which the differential somatic evolutionary patterns are drawn are oblivious of confounding factors of somatic evolution described in other somatic tissues, such as age, alcohol consumption, smoking and sex. Beyond the questions about whether significance can hold while adjusting for these covariates, the study misses what in this reviewer's view is an interesting question by itself, namely, whether there are any discernible effects which may be statistically attributable to either of these factors?

Thank you for highlighting this important issue. We agree that age, and sex, as well as tobacco and alcohol exposures may all impact somatic evolution, as has been demonstrated in hematopoiesis, bladder, and oral epithelium for example[1, 2, 3]. As in most other tissues, the bulk of the mutational burden of normal esophagus is attributable to the 'clock like' mutational signatures SBS 1, 5 and 40 that increase in proportion with age[1, 4, 5]. Smoking and alcohol exposure also impact mutational burden, mutational signatures, and mutant selection[1, 5].

We have now analysed the effect of potential confounders on the mutational landscape in this study. To explore whether this association could arise from other patient features, we analysed the associations between different 'factors' (characteristics of the patient) and 'responses' (measured mutational features). Factors in our study are treatment, age, gender, smoking, tumour type, and tumour location. For the Nanoseq data, the responses are Substitutions/Mb, and Indel/Mb, and the signatures SBS288A/Mb and SBS28B/Mb. For targeted data, the responses are the density (/mm^2) of the total number of mutations, SBS, DBS, Indels, mutational type (missense, nonsynon, splice, frameshift, nonsense), positively selected mutant genes, and the percentage of epithelium mutant for the genes under positive selection.

We performed Kruskal-Wallis non-parametric tests for each factor and response pair, except for age, the only continuous variable, for which we used ANCOVA. For each test, multiple test correction was performed using the Benjamini-Hochberg method and p-values adjusted accordingly. This analysis is reported in a statistical **Supplementary Note**.

For the Nanoseq data, we found a significant association between treatment type and indel burden (Indel/Mb), and a borderline significant association of treatment and substitution burden (SBS/Mb), using (**Reviewer Table 1**).

For targeted data, we found a significant effect of treatment on the percentage of epithelia mutated for *TP53*, *PPM1D*, *SPOP* (**Reviewer Table 1**).

We conclude that the only significant effect on responses is from treatment and that the other factors do not confound our analysis.

P_response	ME_treatment	ME_Sex	ME_Smoke	ME_Tumourtype	ME_Tumourlocation	ME_Age
**Nanoseq**						
p_Indel	0.0003	0.9295	1	0.9295	1	0.9295
p_Sub	0.0734	0.3705	0.3705	0.3705	0.7072	0.0734
p_SBS288A	0.905	0.5431	0.8518	0.905	0.905	0.905
p_SBS288B	0.1163	0.7007	0.4527	0.7007	0.329	0.318
**Targetted data**						
p_Mutations	1	1	1	1	0.396	1
p_SBS	0.915	0.915	0.915	1	0.915	0.915
p_DBS	0.25	0.988	0.988	0.723	0.723	0.09
p_Indel	1	1	1	1	0.08	1
p_Nonsyn	1	1	1	1	0.396	1
p_Missense	1	1	1	1	1	1
p_Frameshift	1	1	1	1	0.08	1
p_Splice	0.796	0.796	0.876	0.876	0.796	0.876
p_Nonsense	0.31	0.261	0.785	0.261	0.399	0.387
Percentage mutant epithelia						
p_NOTCH1	0.93	0.93	0.344	0.93	0.93	0.344
p_TP53	0.05	1	1	0.93	0.276	1
p_PPM1D	0.01	0.757	0.32	0.301	0.757	0.757
p_MTOR	0.221	0.782	0.221	0.171	0.761	0.299
p_NFE2L2	0.926	1	1	1	1	1
p_SPOP	0.044	0.806	1	1	0.1	1
p_RAC1	0.386	1	1	1	1	1
p_HLAB	0.166	0.875	0.48	0.639	0.921	0.76
Mutation density						
p_NOTCH1	0.531	0.907	0.907	0.268	0.531	0.952
p_TP53	0.379	1	1	1	0.349	0.379
p_PPM1D	1	1	1	0.602	1	1
p_MTOR	1	1	1	1	1	1
p_NFE2L2	1	1	1	1	1	1
p_SPOP	0.099	0.708	0.784	0.708	0.117	0.708
p_RAC1	0.44	1	1	1	1	1
p_HLAB	0.099	1	0.532	0.852	1	1

Reviewer Table 1. Associations of treatment and other factors and responses: Multiple test corrected p-values of Kruskal-Wallis tests are shown, except for age which was tested by ANCOVA. Effects where corrected p≤0.05 are highlighted.

In the Nanoseq data, there is an association between treatment and SBS and indel burden (**Figure 1d, f**), but no association was observed with sex, smoking status, tumour type, tumour location or age. Although we may expect an association of SBS burden with age, the age effect was of borderline significance (p=0.07). This may reflect the narrow age distribution and number of patients in our cohort. We find no association between mutations attributed to SBS288A and SBS288B and treatment or any other factors.

In the targeted data we find an effect of treatment on percentage of epithelia mutant of *TP53* (detailed in **Fig. 2b**), PPM1D (**Fig. 2c**), and SPOP. However, no associations with sex, smoking status, tumour type, tumour location, or age were found. None of the associations with treatment were confounded by gender, age, smoking status or tumour type. We have detailed all our analyses in the supplementary statistical note.

Caveats with this analysis are the size of the study, and the reliability of smoking history. We have added a paragraph on the limitations of the study in the discussion as follows:

'Limitations of this study include the relatively small number of patients per treatment group and the available metadata concerning environmental exposures. We found significant associations between some measures of the mutational landscape and treatment. Larger studies will be needed to explore whether factors such as age, sex and smoking interact with treatment to shape the normal tissue mutational landscape.' Line number 565-569.

- The only five SCC cases in the cohort belong to the CROSS group. Is there any specific features of these samples clonal composition wise? Are there any clonal structure differences when accounting for different anatomical sites? In sum, are there any chances that these samples are biasing the results reported for CROSS?

Thank you for highlighting this potential confounder of the results in the CROSS group. All SCC patients fit for neoadjuvant treatment were given CROSS following standard protocols in the hospitals in the study. We find no confounding factors in terms of sex, tumour type, tissue location and smoking status (see **reviewer table 1** above).

We also compared the expansion of *TP53* and *PPM1D* mutant clones in normal esophagus in AC and SCC patients within the CROSS group, finding no significant difference between tumor types. Both mutant genes were significantly increased compared with the NT group (**Reviewer Figure 1**).

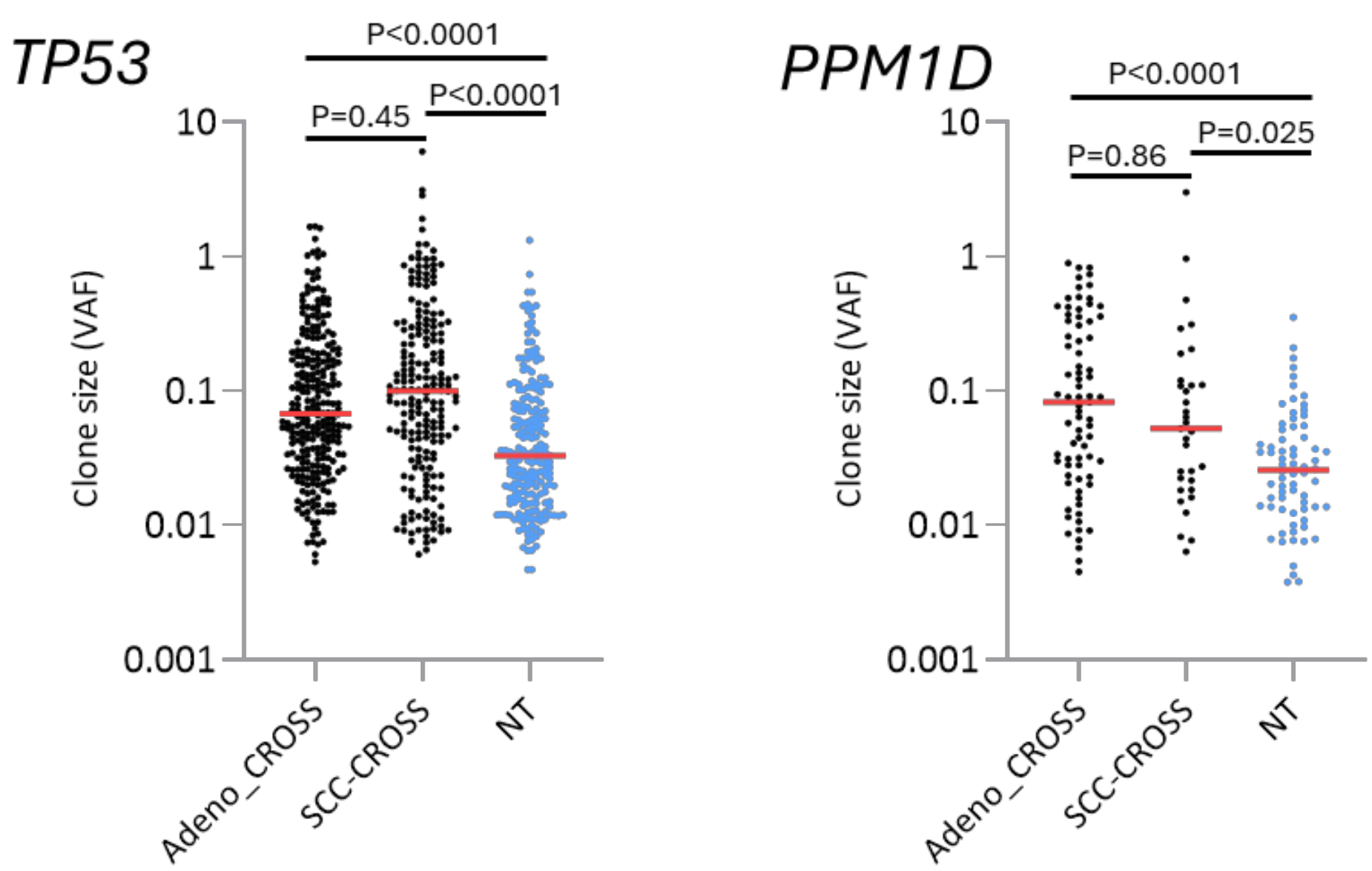

Reviewer Figure 1: Clone size distribution for *TP53* and *PPM1D* mutants in NT patients and CROSS patients with Adenocarcinoma (Adeno_CROSS) or SCC (SCC_CROSS), Kruskal-Wallis test with Dunn's multiple comparison test.

Similarly, the distribution of missense mutations in *SPOP*, which are also positively selected in CROSS patients, did not differ by tumor type (**Reviewer Figure 2**).

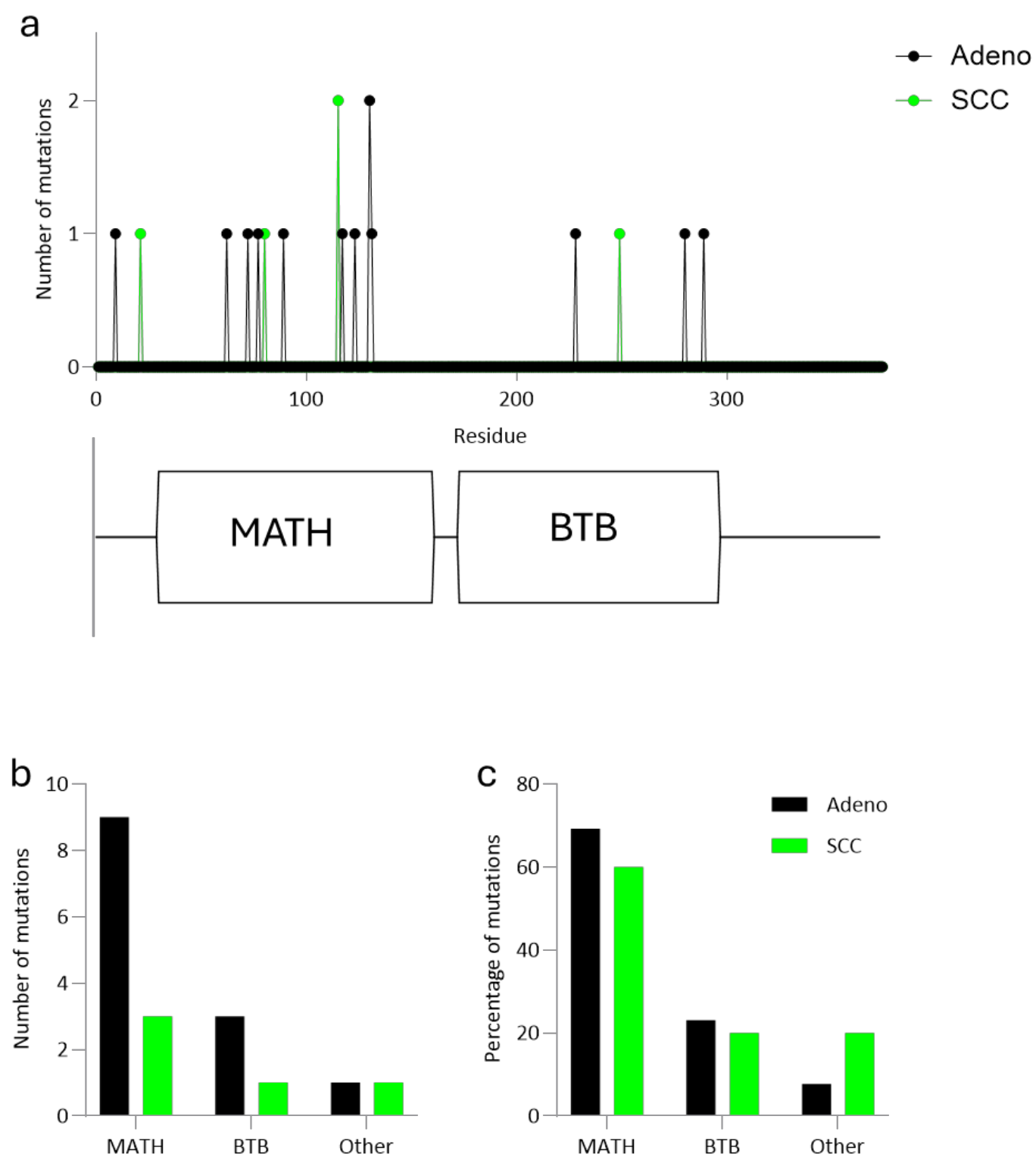

Reviewer Figure 2: a. Distribution of mutations across the SPOP protein in CROSS-Adeno and CROSS-SCC patients. The MATH and BTB domains are indicated. **b, c** – Number (**b**) and percentage (**c**) of mutations found in the MATH, BTB domains of SPOP in CROSS-Adeno and CROSS-SCC patients. Clustering association with tumour type (Adeno/SCC) p=1, smoking p=1, Tumour location p=1.

As expected, tumor location differed between SCC and AC patients (4/5 SCC were located in the middle third and 5/6 AC in the distal esophagus/cardia). As discussed above, tumor location did not impact the mutational landscape.

We now discuss these results in the text as follows:

'Most patients had esophageal adenocarcinoma (AC), only 5 had SCC and all of these were in the CROSS group. However, neither within the CROSS group nor over all groups was there any significant variation in any parameter with tumor type (**Supplementary statistical note, Extended data table 5, Table 5**).' Line number 202-205

- The authors do not provide a minimum correlation study that informs which covariates to include as potential confounding factors. Given the effect sizes that the authors report, it seems plausible that most results will continue to hold after adjusting for covariates, whichever are relevant in the light of the correlation analysis. But this should be provided as evidence that there is no confusion due to the cohort composition.

Thank you for raising this important point. As discussed above we now report our investigation of potential confounders in the Supplementary statistical note.

- Despite the relevance of the absence of chemotherapy-induced mutagenesis to draw the

conclusion that the relatively expanded clones were preexisting, the manuscript does not provide a concrete description of the signature deconstruction approach followed. Did the authors extract the signatures de novo? Did they refit with a predefined compendium of signatures learned from esophageal tumors? In the methods section only a generic paragraph enumerating the tools is provided, which in my opinion is not sufficient to cast reliability on the result.

Thank you for raising these important issues. We have revised our presentation of the mutational signature analysis and the description of this in the methods as follows:

'Mutational matrices for SBSs were generated using SigProfilerMatrixGenerator with default options (v1.2.12). SBSs signatures were extracted using SigProfiler extractor (v1.1.12) using default parameters, the ndsvd_min initialization algorithm used was nndvsd_min and the number of NMF replicates was 500. De novo SBS signatures were extracted within the SBS288 context. For the nanoseq analysis the optimal number of signatures using the mean sample cosine distance and average stability was determined to be 2: SBS288A and SBS288B. Deconvolving these signatures onto the known COSMIC signatures (v.3.4; cancer.sanger.ac.uk/signatures) using SigprofilerAssignment (V0.0.13) showed signature A comprised a combination of SBS1 (10%), SBS5 (49%) and SBS40a (41%) (cosine similarity 0.97), whereas signature B consisted of a combination of SBS1 (4%), SBS5 (17%), SBS16 (24%) and SBS40a (54%) (cosine similarity 0. 98). For the genome samples signatures were extracted in the 1536 context and all other parameters remained the same.' Line number 777-886.

We now present the analysis of SBS signatures in the main text as follows:

' De novo signature analysis revealed two signatures SBS288A and SBS288B (**Fig. 1e, Extended data Fig. 2, 3, Extended data Table 3**). Signature A was a combination of the 'clock like' signatures SBS1 (10%), SBS5 (49%) and SBS40a (41%) (https://cancer.sanger.ac.uk/signatures/sbs/, cosine similarity 0.97). Signature B showed a combination of SBS1 (4%), SBS5 (17%), and SBS40a (54%), together with SBS16 (24%) (cosine similarity 0. 98) (**Fig. 1e, Extended data Table 3**)[18]. SBS16 is correlated with alcohol ingestion in normal esophagus and ESCC (**Extended data Table 3, Extended data Fig. 4**)[19]. There was no significant effect of treatment in the number of Signature A mutations/Mb (p=0.905, Kruskal-Wallis Test, **Supplementary note, Extended data Table 4)** or Signature B mutations/ Mb (p=0.1163 Kruskal-Wallis Test, **Supplementary note, Extended data Table 4**)'. Line number 92-102

De novo signature profiles are shown in Extended data Fig. 2. Per patient attributions of de novo and deconvolved signatures are shown in Extended data Figs 3 and 4 respectively.

- The above mentioned combined evidence of clonal differences, comparable mutation burden and absence of activity of chemotherapy-related mutational signatures, suggests the selective expansion of preexisting mutant clones. Are there any clear signals of negative selection gene wise?

We were unable to detect negative selection (dN/dS<1) in this study, but this does not mean it did not occur, indeed mutations that increase susceptibility to chemotherapy are likely to be present in the tissue prior to exposure. We have detected negative selection in two previous targeted sequencing studies in the skin. We speculate this is because of the large number of mutations detected across the 75 gene panels used (48000 and 94000 SBS[7, 8]). In both studies we obtained sufficient synonymous mutations to detect negative selection in multiple genes. In this study we found a total 20,278 SBS across 324 genes, which may explain the absence of detectable negative selection. We comment on the lack of sufficient power to detect negative selection as one of the limitations of the study in the discussion.

‘It should be noted that we were unable to detect negative selection within this study (defined as dN/dS<1). Detecting negative selection requires sufficient synonymous mutations to reach significance. We only detected 3,669 synonymous mutations across all 324 genes and speculate that the study was underpowered to detect negative selection. ‘ Line number 569-573.

The authors mention briefly in the discussion the case of PTEN mutant clones, which seem to be positively selected in normal epithelium, although this effect seems to be lost upon treatment. I also wonder whether this can be a numerical artifact due to the low number of samples in the treatment groups compared to the non-treated.

This is an important point. We now present a complete listing of dN/dS values for all genes (**Extended Data Tables 6-10**). From this it can be seen that mutant *PTEN* is significantly selected in the NT group (global q 0.0069) but not in the treated groups, though in CROSS patients mutant *PTEN* selection is borderline (global q 0.0622), see **Reviewer Table 2.**

	Number of mutations					dN/dS ratio				
	Synonymous	Missense	Nonsense	Splice	Indel	wmis_cv	wnon_cv+wspl_cv	wind_cv	pglobal_cv	qglobal_cv
CROSS	0	14	1	0	4	3.96	2.65	3.29	0.00	0.0622
ECX	0	1	0	0	0	0.80	0.00	0.00	0.99	0.9990
EOX	0	3	0	0	1	3.48	0.00	5.52	0.15	0.9994
FLOT	0	7	0	1	0	4.18	5.73	0.00	0.18	0.9986
NT	1	13	1	0	5	4.66	3.79	7.57	0.00	0.0069

Reviewer Table 2: dN/dS values for *PTEN* mutants.

However, there are 0 synonymous mutations for all treatment groups except NT. We applied the ‘rule of three, such that if no events are observed in a group, then the upper confidence interval limit for the occurrence of events is three, and the risk is 3/N, where N is the sample size of the group[9]. This leads us to a minimum of 8 nonsynonymous mutations to determine the significance of dN/dS values if there are 0 synonymous mutations. We explain this in the methods as follows

‘In general, P values <0.05 were considered statistically significant, but for dN/dS ratios calculated by dNdScv, a global q<0.01 was considered statistically significant. To reduce a probability of artificially inflated dN/dS ratios in genes for which there were no synonymous mutations[10, 11], we filtered such genes if the total number of nonsynonymous mutations was 8 or fewer. This cut off is based on estimating the probability of observing no synonymous mutations in a sequence of mutations and hence the likelihood of dN/dS being artificially inflated. This requires estimating the probability of a ‘non-event’. It is possible to put an upper confidence limit on event risk, when no events are observed in a sequence of trials. A simple ‘rule of three’ has been proposed such that if no events are observed in a group, then the upper confidence interval limit for the occurrence of events is three, and the risk is 3/N, where N is the sample size of the group[9]. The value 3 coincides with the upper limit of one-tailed 95% confidence interval from the Poisson distribution. It is 3.7 for 97.5% confidence interval, and 4.6 for 99% confidence interval. Based on this we estimate the minimum number of nonsynonymous mutations should be at least 8 for 99% confidence (1-0.01) that a dN/dS value is reliable if there are 0 synonymous mutations.’ Line number 809-821.

As we have insufficient synonymous mutations to determine dN/dS values for *PTEN* mutants in three of the five treatment groups, we have now removed the comment on mutant *PTEN* selection in the NT group from the text.

-The use of resistance as leitmotif throughout the paper and the conceptual link with treatment resistance in tumors renders, in my opinion, a somewhat confusing framing of the results. The study addresses somatic evolution in the normal esophagus after exposure to cancer therapy,

a system with evolutionary constraints that differ from those of a tumor. The authors posit that from the study of clonal dynamics in the normal esophagus a catalog of potential targets implicated in cancer therapy resistance can be learned. The reviewer agrees with this view. But the authors must make sure that they draw a very clean distinction between the normal and tumor evolutionary systems. In my opinion, the possibility of finding false candidates should be explicitly discussed, as well as presenting what are the new potential targets that the study contributes.

Thank you for this important corrective comment. We agree that selection in normal tissues may differ profoundly from that in a tumor and that normal tissue findings cannot be simply extrapolated to tumors. We have therefore revised the text as follows:

Title:
Cancer treatment alters mutant selection in normal esophagus

Abstract:

'In the FLOT group there was increased selection for *RAC1*, *NFE2L2* and *MTOR* mutations consistent with these mutants conferring 5-FU resilience in normal epithelium. Sequencing normal epithelia reveals treatment specific selection of mutations and may identify genes implicated in cellular responses to therapy. Line number 40-42

Discussion:
'The enhanced selection of mutants in a treatment specific manner is notable. Activating mutants of *MTOR*, *RAC1* and *NFE2L2* were selected after treatment with FLOT, which differs from the schedules in containing 5-FU, whose activity is potentiated by Leucovorin[12]. The selected mutants are gain of function and may protect normal esophageal cells against 5-FU. Do these findings in normal tissue correlate with treatment resistance in cancer with its altered genome and micro-environment? MTOR and NFE2L2 activation is linked to 5-FU resistance in tumors and is associated with 5-FU resistance in cancer cells[13, 14, 15, 16]. There are no reports linking *RAC1* mutants conferring 5-FU resistance clinically, though expression of the RAC1b isoform confers 5-FU resistance in vitro [17]. It cannot be assumed that mutant selection in normal tissues will be the same in cancer cells. However, identifying mutants selected by treatment in normal tissues may provide a catalogue of potential targets that modulate cellular resilience to therapy, which may guide investigation of tumor resistance.' Line 551-561.

Minor issues

=========

- The method 1D NMC is not described in the Methods section (but it is referenced in l. 353)

The 1D NMC methodology is now described in the methods as follows:

'1D Clustering analysis
To identify positional clustering of missense mutations along the primary amino-acid sequence, we performed 1D non-random mutational clustering (NMC) analysis on positively selected genes within each treatment group separately. Only non-synonymous missense mutations were included in this analysis. We performed NMC clustering using the NMC function within the iPAC R package[18]. Clustering significance was assessed against the null hypothesis that mutations were randomly distributed along the coding sequence, and multiple-testing correction was applied within each gene/treatment-group analysis. Clusters with

adjusted *P* < 0.05 were considered significant. For visualisation, lollipop plots show the significant clusters identified by NMC.' Line number 830-837.

- How did the authors correct for uneven sequencing depth per site and sample when computing mutagenesis, dN/dS and other surrogates of selection and compared them?

We thank the reviewer their comment. In light of this we have now removed samples below 250x coverage, as above this depth we should be able to reliably call mutations of >1% VAF. We have also removed a single sample of >800x coverage to avoid artefacts associated with over-sequencing.

We have further verified that there is no association between number of mutations/kb/ gene and coverage. We provide a summary as **Reviewer Figure 3** below, and the full analysis including the code used is available on https://doi.org/10.5281/zenodo.19627198. We have not included this figure in the revised manuscript at present but would be happy to do so if the reviewer wishes.

We have added the following sentence to the methods:

'We saw no association between number of mutations/kb/gene and the mean gene sequencing coverage (R^2=0.01), see https://doi.org/10.5281/zenodo.19627198 for analysis and code.' Line number 735-736.

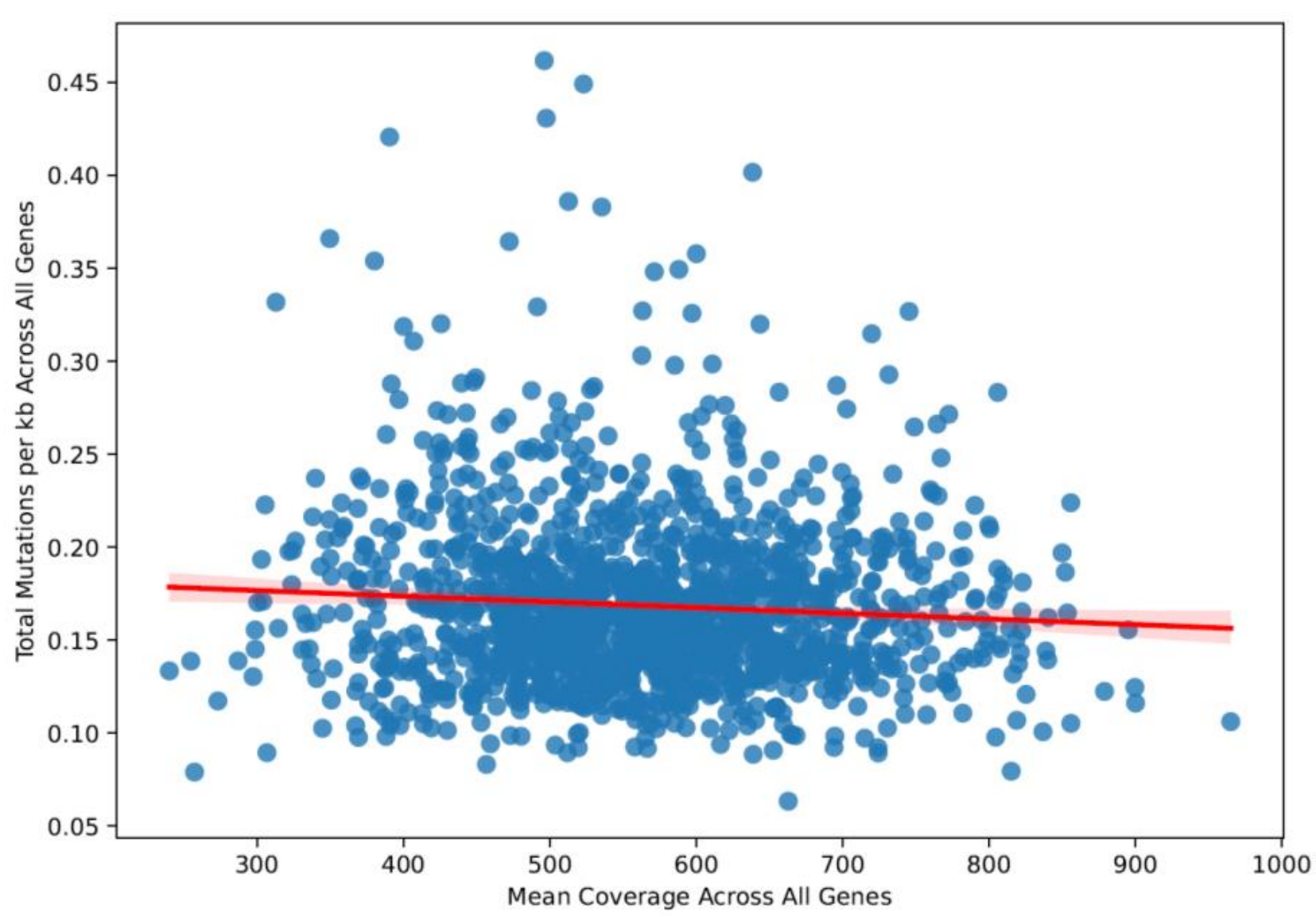

Reviewer Figure 3: SBS per Kb plotted against mean coverage across all genes, each dot is a gene. There is no association between the total mutations/ kb/gene and coverage (r^2=0.01).

- Supp. Fig. 5b-d, Supp. Fig. 6, what happened with the chromosome lengths?

ASCAT linearly scales the chromosome lengths in accordance with the usable SNPs. As different individuals will have different numbers of heterozygous SNPs the chromosomal lengths are scaled accordingly. We have revised the figure legend to explain this:

‘Extended data figure 7 Copy number alterations detected by targeted sequencing
a Percentage of $2mm^2$ grids carrying copy number events covering the *NOTCH1* locus (chr9: 139388885-139407300). CROSS n=610, ECX n=260, EOX n=163, FLOT n=401, NT n=456. P-values derived from Fisher's exact test with Benjamini-Hochberg multiple test correction. Examples of altered copy number at the *NOTCH1* locus (**b**), *TP53* locus (**c**) and *HLA* locus (**d**). In panels **b-d** the depicted chromosome lengths are linearly scaled according to the number of usable heterozygous SNPs. ‘ Lines 337-342.

‘Figure 5 Whole genome sequencing reveals CNA in CROSS patients
a-d Examples of ASCAT profiles showing copy number changes in typical clones from NT (**a**) and CROSS groups (**b**-**d**); ranging from small focal LOH events (**b**) to whole chromosome gains (**c**). In occasional cases multiple complex events were identified (**d**). Decomposed signatures are shown below the ASCAT profile along with potential driver mutations and associated VAF. The depicted chromosome lengths are linearly scaled according to the number of usable heterozygous SNPs.’

- l. 361-362: Should this be pointing to the Methods section?

We now say:

‘Using computational elastic network modelling we predicted the conformational dynamics of the protein (**Methods**).’ Line number 433-434.

- l. 366-367: Non-treated show significantly increased number of mutations within the hinge residues. Increase with respect to what? Significantly higher than expected by neutral mutagenesis?

We have now clarified the text as follows:

‘Both FLOT and NT groups show significantly increased numbers of mutations within the hinge residues compared to an expected Monte Carlo sampled distribution based on the mutational spectrum of each treatment group.’ Line number 439-441.

- l. 717: What does "statistics related to mutational selection" mean? Could the authors be more specific?

We have clarified this as related to structural mutational selection and details of the statistical approach used is more fully described within the structural mutational selection analysis section in the methods.

- Paragraph starting at l. 722: could the authors succinctly explain here how is the so-called 3D protein structure enrichment computed?

This is now explained in the methods as follows:

‘Structural mutational selection

We performed calculation of selection via comparing observed properties with those expected if only the mutational signature was occurring in the tissue. We have applied this method previously to selection of mutant clones in the skin[8]. Mutations observed in each group of patients (CROSS, ECX, EOX FLOT, or NT) were used to calculate the mutational spectrum

for each condition. For each gene studied, the calculated signature was then used to generate an expected distribution for the mutational property being investigated. For example, for mutations observed in the kinase domain, the number of mutations expected in the kinase domain is calculated by taking the mutational spectrum, total number of mutations observed in that gene, and the DNA sequence of the kinase domain. This allows the generation of how many mutations would be expected to be seen in the kinase domain if the only process occurring was mutation consistent with the mutational spectrum. This expected number is then compared to the observed and a p value generated using a binomial test. For p values relating to continuous metrics we apply a Monte Carlo test applied to distances (using differences in median distance to a defined part of the structure such as a small molecule to calculate the p value) and binding energy calculations (using differences in mean energy to calculate a p value).' Line number 841-857.

And for the energy calculations as follows

'Energy calculations are performed using Foldx[19]. We calculate every possible mutation for folding energies, and every possible mutation within 10Å of the ligand for binding energies. Code for calculating binding energies is available at https://github.com/shorthouse-lab/binding_ddg. These energies are then used to construct the expected distribution. To generate Rapamycin parameters for use with Foldx, we used pyfoldx[20]. Ligand parameters were generated following a protocol described previously in which each atom within the ligand is mapped to a chemically matched FoldX recognised atom name, and coordinates from structures are used to build the ligand file that is recognised by FoldX[21].' Line number 860-866.

- Same paragraph: written "signature" where it should be read "profile"

We have amended this line as follows:

'This allows the calculation of how many mutations would be expected to be seen in the kinase domain if the only process occurring was mutation consistent with the mutational spectrum.' Line number 850-852.

- l. 50-52: The authors state "we hypothesized that anticancer treatment may selectively expand mutant clones that confer resistance to treatment in the densely mutated normal esophagus". It might seem that what is implied is that the clonal status in the normal esophagus is somehow inducing the therapy response of the tumors adjacent in the same organ. I do not think this is what the authors intended to imply, although it leads to an interesting question, namely, whether there is any causal link between the evolutionary bottlenecks in the normal esophagus upon cancer treatment and the response to treatment of an hypothetical tumor arising in the same esophagus. This might well be true, but the current study design does not allow to make claims of this kind.

Thank you for highlighting the potential of this comment to create misunderstanding. The clones in normal esophagus are not adjacent to the tumor. We have no evidence to suggest, and consider it unlikely, that normal esophageal mutants confer resistance on the tumor. We agree that the interesting hypothesis of whether evolutionary bottlenecks in the normal esophagus impact tumor resistance cannot be addressed in this study. We have therefore revised the text as follows:

'We hypothesised that anticancer treatment may alter the selection of mutant clones in the already densely mutated normal esophagus.' Line number 51-53

Reviewer #2 (Remarks to the Author):

I greatly enjoyed the manuscript by Fowler et al., and while there are interesting assertions worthy of publication at this level, the work falls short of being suitable at this time because of fundamental analytical shortcomings. I would support acceptance on resubmission, should the major issues that I raise be addressed and no subsequent issues arise. ==

Summary
Originality and significance: The work is novel and will be of general interest
Data & methodology: The data are generally of high quality, though biases in sampling are not clear

Appropriate use of statistics and treatment of uncertainties: The statistics driving many statements are occasionally superficial, lacking the appropriate rigor for suitable assertion of their conclusions.

Conclusions:
Alternative explanations addressing potential sources of uncertainty are not applied readily. Interpretation of these results and potential future gaps in our knowledge would benefit from these being stated. Though their findings make theoretical sense, the inappropriate analyses used to test their system weaken their arguments.
Suggested improvements: More rigorous analyses and application of better statistical inference. It's not immediately obvious how the associated github repo fits with the results presented- cleaning that up would improve accessibility to the author's conclusions and methods.

Thank you for highlighting these important points. We have reanalysed data and added statistical analyses as set out below, which are now described in a Supplementary Note. We also have revised the annotation of the Github repository to improve its accessibility.

References: The authors should check for previous evidence for some of their major findings- that there is evidence for chemotherapy in sequencing data and that PPM1D confers radio-resistance.

Thank you for highlighting these points. There are indeed studies showing the presence of treatment induced mutations in normal tissues other than the esophagus in cancer patients. We now cite this literature as follows:

'…However, the mutational signatures of 5-FU, oxaliplatin or carboplatin (SBS25, 31 and 35, https://cancer.sanger.ac.uk/signatures/sbs/) were absent [22, 23, 24]. This may reflect the selective loss of normal esophageal cells damaged by these agents and contrasts with the detection of chemotherapy signatures in other normal tissues in cancer patients[25, 26, 27, 28]. ' Line number 102-105.

Increased selection of *TP53* and *PPM1D* mutant clones in other normal tissues has also been reported in cancer patients. As well as the extensive literature on clonal haematopoiesis, positively selected *TP53* and *PPM1D* mutants have found in the lung and liver following both chemotherapy or immunotherapy [26, 29, 30, 31, 32]. We now cite this work as follows:

' Selection for *PPM1D* and *TP53* has been observed in other normal tissues including the haematopoietic system, liver and lung in patients treated with radiation, platinum compounds, *TOP2A* inhibitors or immunotherapy[26, 29, 30, 31, 32].' Line number 541-543.

Furthermore, truncating mutations in *PPM1D* exon 6 have a been shown to confer resistance to DNA damaging agents. We have augmented our discussion of this point and citing additional references as follows:

'As in subjects without cancer, over 90% of *PPM1D* mutations in all groups were nonsense or frameshift mutations within exon (**Fig. 3a, b**)[1, 5]. Such mutants generate a stabilized form of the protein which retains phosphatase activity, inhibits TP53 function, blocks the DNA damage response and confers resistance to chemotherapy and ionizing radiation in mutant hematopoietic stem cells, leading to clonal hematopoiesis[31, 32, 33, 34, 35, 36].' Line number 263-267.

Clarity and context: For the most part the manuscript is extremely well written and clear.

Theory states that populations will face selective pressure from strong, persistent environmental perturbation. It stands to reason that somatic tissue will undergo selection when faced with pressure from chemotherapeutic regimes.

Fowler et al. attempt to explore this through characterisation of a cohort of patients with esophageal cancer, for whom they are able to group by different therapeutic regimes, each involving exposure to DNA damaging agents. They biopsy normal tissue and dissect it into multiple gridded subunits, before using nanoseq to demonstrate treatment-specific mutational patterns across ~30% of the genome. No mutations in these data are attributed to any of the chemotherapeutic exposures, therefore the authors reason that clones existed before treatment and that any difference in mutation rate between groups reflects selection for different mutants as a result of treatment regime. They subsequently apply targeted, high depth sequencing to many more gridded subunits to define signals of selection across a panel of 324 genes. A range of subsequent analyses are performed with the aim of exploring the functional consequences of mutations under selection.

======================================

Major points

1) Mutation signature analysis not robust enough

That none of the mutations from mutagenic exposure are found is surprising, given the range of evidence found using the same techniques across multiple tissue types already published and as partly alluded to in the text. There is no evidence of smoking, though many of the patients are, or were, smokers. Given the importance of this finding in driving the narrative, it is noteworthy that there are many discrepancies in the characterisation of mutational processes in this work.

The authors list use of Sigprofiler extractor, but there is no evidence that they performed de novo extraction of signatures. Figure 1e, which is a summary of the mutation signatures in these data, isn't representative of the heterogeneity of signals across the cohort. For example, the signal from SBS16 represents only 3 samples in CROSS, and some of the samples have a surprising lack of diversity; 2 are 100% SBS40 and 16 have no SBS5.

Thank you for highlighting this point. We have now performed de novo SBS signature extraction in the SBS288 context for the Nanoseq data. The optimal number of signatures using the mean sample cosine distance and average stability was determined to be 2. Deconvolving these signatures onto the known COSMIC signatures (v.3.4; cancer.sanger.ac.uk/signatures) using SigprofilerAssignment (V0.0.13) showed signature A comprised a combination of SBS1 (10%), SBS5 (49%) and SBS40a (41%) (cosine similarity 0.97), whereas signature B consisted of a combination of SBS1 (4%), SBS5 (17%), SBS16 (24%) and SBS40a (54%) (cosine similarity 0. 98). Importantly de novo deconvolution does not reveal any known signature(s) attributed to chemotherapy (**Fig. 1e, Fig. S2, Fig. S3**).

We also calculate mutations/Mb in all subjects. This shows no significant difference between treatment groups for Signature A or Signature B (See statistical note).

We now comment on the de novo signature extraction as follows:

'De novo signature analysis revealed two signatures SBS288A and SBS288B (**Fig. 1e, Extended data Fig. 2, 3, Extended data Table 3**). Signature A was a combination of the 'clock like' signatures SBS1 (10%), SBS5 (49%) and SBS40a (41%) (https://cancer.sanger.ac.uk/signatures/sbs/, cosine similarity 0.97). Signature B showed a combination of SBS1 (4%), SBS5 (17%), and SBS40a (54%), together with SBS16 (24%) (cosine similarity 0. 98) (**Fig. 1e, Extended data Table 3**)[18]. SBS16 is correlated with alcohol ingestion in normal esophagus and ESCC (**Extended data Table 3, Extended data Fig. 4**)[19]. There was no significant effect of treatment in the number of Signature A mutations/Mb (p=0.905, Kruskal-Wallis test, **Supplementary note, Extended data Table 4**) or Signature B mutations/ Mb (p=0.1163 Kruskal-Wallis test, **Supplementary note, Extended data Table 4**)' Line number 93-102.

These results are shown in **Extended data Figs 2-4** in the manuscript. We now include SBS288A and SBS288B in the summary of the signature data in **Fig. 1e**.

In Lawson et al. 2025, where nanoseq was applied to a much greater depth, SBS5 is deconvoluted in a mixture along with SBS1 and presented as SBSA, for which not a single individual lacks signal- see figure 4a and c inhttps://www.nature.com/articles/s41586-025-09584-w. [nature.com] That Lawson et al. are unable to distinguish well-known and generally ubiquitous signatures suggests that de novo deconvolution is vital for recognising the active mutational processes in these data, as they may appear differently, or even as composites of previously documented signatures.

We agree with the importance of de novo deconvolution, which is well demonstrated in Lawson et al. as the reviewer says. Please see our response above.

Looking across the rate of substitutions per Mb suggests that many of the mutations in these data are unattributed during signature fitting, e.g. PD47501 has a Sub/Mb of 0.59 and, when multiplied by 3200 Mb, suggests that there are likely 882 unattributed mutations, with 1006 attributed across the 4 signatures. This also suggests that the mutational signature fitting has not been adequately performed.

Nanoseq samples only part of the genome[37]. Hence, for PD47501, 0.59 Sub/Mb * footprint (1704640266bp) gives 959 mutations (1014 corrected). Sigprofiler assigns 1006 mutations so there is good agreement rather than a large proportion of unattributed mutations when the footprint is taken into account. To avoid confusion, we now present the signature mutations as per Megabase to clarify the results presented in **Extended data Table 3**.

As described above, we have re-run Sigprofiler and find the attributable burden correlates well with that from Nanoseq (**Reviewer Fig 4**).

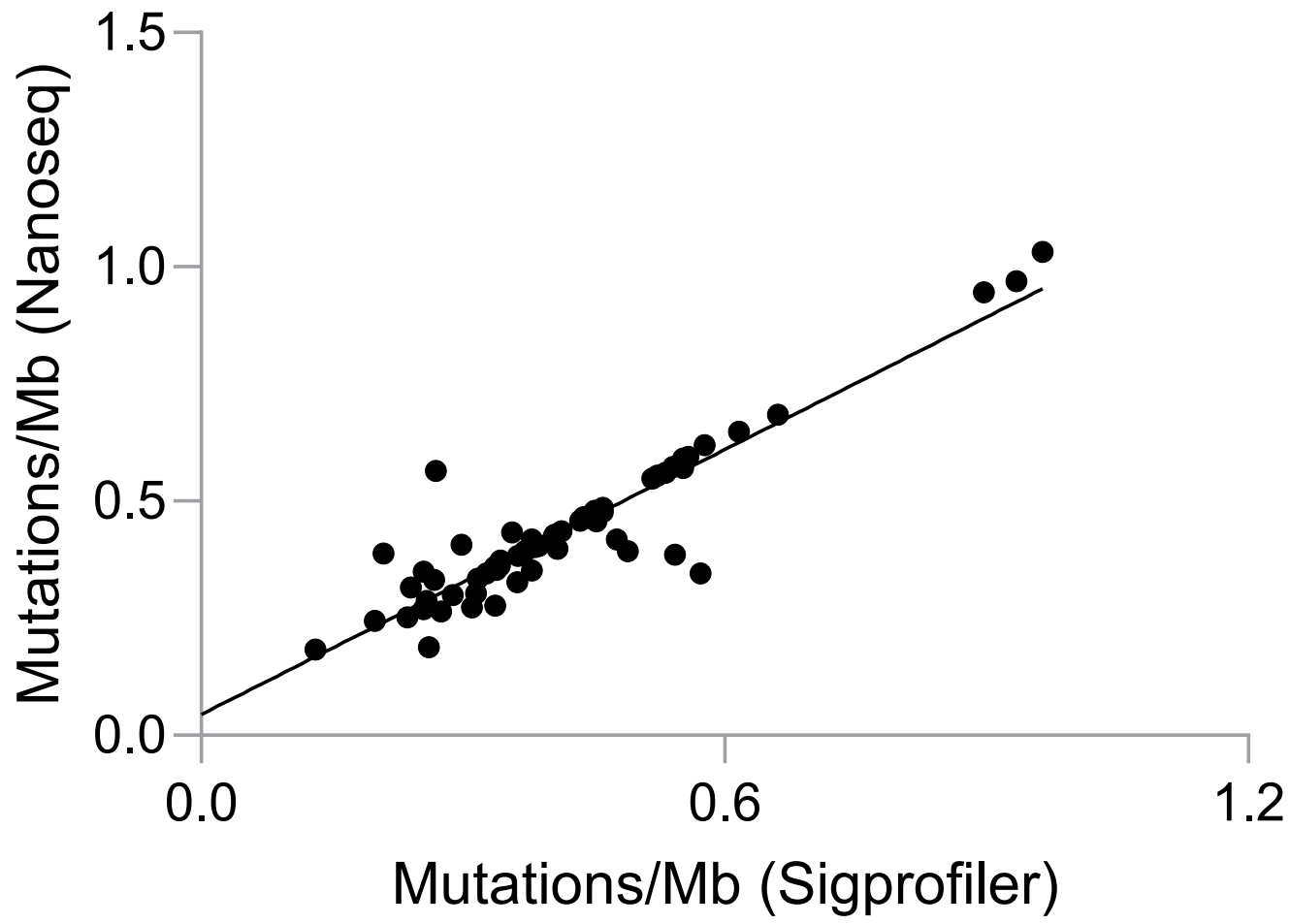

Reviewer Figure 4: Comparison of number of mutations/Mb assigned by Nanoseq and number of mutations/Mb from Sigprofiler signature assignment. R^2= 0.85, p<0.0001.

In order for this work to sufficiently address these shortcomings, the authors should provide evidence of de novo deconvolution and strive to find alternative signals for exposure in their data. For example, fitting of double base substitutions associated with platinum-based therapies and tobacco smoke would be useful. It is possible that mutational signatures among individual samples could form the bases for a cleaner analysis, e.g. further justification for the inclusion of the outlier in NT, and the two in CROSS.

We have investigated fitting DBS signatures which as the reviewer says are associated with platinum-based therapies and tobacco smoke. Unfortunately, DBS are rare in our dataset (total DBS=428 over all 69 patients). With an average of under 7 DBS per patient and over half the patients (43/69) having 5 or fewer DBS, we submit there are too few DBS to run reliable DBS signature analysis by treatment group.

To investigate whether the maximal values of SBS/Mb and SBS288B/Mb observed in CROSS and NT are outliers or are likely to be expected by random and uniform values, we performed a simulation. We selected intervals from the minimal to maximal values of SBS/Mb (+/-2.5% of intervals from minimal to maximal values), and randomly and uniformly sampled from this interval 10000 times, generating simulated distributions of values for CROSS and NT values respectively, shown in blue in **Reviewer Figure 5**.

Then we counted the number of simulated values beyond than our 'outlier' values (>0.97 for CROSS, > 1.03 for NT, see red thresholds (**Reviewer figure 5**). For each simulation, the percentage of values more extreme than these thresholds was ca. 7-9 %, i.e. not significant at p= 0.05. We concluded that the observed 'outliers' are still likely to arise given the observed distributions.

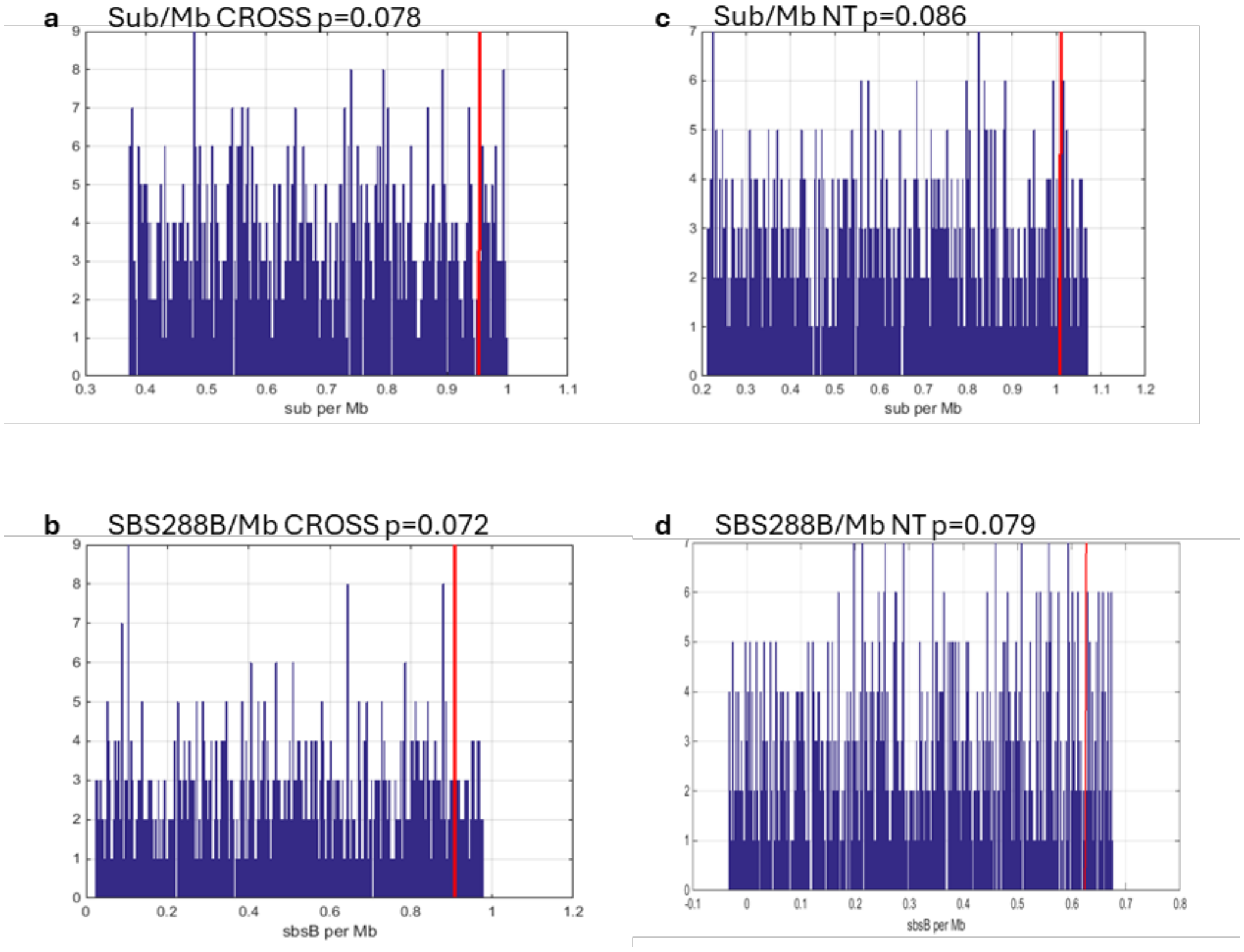

Reviewer figure 5. Simulation of random and uniform values samples from distributions of Sub/Mb (**a**,**c**) and SBS288B/Mb (**b**,**d**) in CROSS (**a**,**b**) and NT (**c**,**d**). Probability of observed maximal value within the distribution is as follows p=0.078 (**a**), p=0.086 (**b**), p=0.072 (**c**), p=0.079(**d**).

The excess of non-exposure derived signatures between NT and treatment regimes would therefore be the measure of selection to move forward with. The presence of signatures from treatment or tobacco smoke doesn't necessarily mean that treatment hasn't caused selection for specific variants already present in the population, however it would need to be stated that the mutations found are possibly the result of enrichment through mutagenesis rather than selection. It would also need to be acknowledged that clock-like processes might be enriched in rapidly expanding populations under selection, though this could be addressed with additional analyses.

This is an interesting suggestion. FLOT, ECX and EOX do not show an increase in burden (or a change in signature, though as the reviewer points out exposure does not always leave a signature) compared to the NT group. It follows that the differences in mutational landscape after treatment may reflect changes in selection of mutations already present in the epithelia rather than the generation of new mutations through treatment. Within the CROSS group there is an increase in burden; suggesting that both the expansion of pre-existing clones and the generation of treatment induced mutations may have occurred. It is certainly possible that multiple cycles of cytotoxic treatment may increase the burden of clock-like signatures as tissues regenerate after each exposure. However, summing SBS/Mb for the clock-like SBS1, SBS5 and SB40a showed no significant difference across the treatment groups (p=0.12, ANOVA). This does not exclude an effect of treatment on mutational burden, as the mutational

impact of short duration of neoadjuvant treatment (3-4 cycles over 8-9 weeks), may be lost in comparison to decades of accumulation of clock like signatures. This leads us to speculate that any increase in treatment related mutational burden may be too small to detect in this study.

2) Confounding sources of variation throughout and superficial statistical analysis

There are many assertions throughout the manuscript that could be made more emphatically if they demonstrated that they weren't potentially an artifact of confounding variables, such as those in the metadata. An example of this is that all 5 of the SCC samples in these data are in the CROSS group (n=11) and is therefore a potentially confounding variable where the CROSS group is suggested to be under selection. More appropriate statistics, aiming to define the role of these effects and that I suggest subsequently, would make for stronger evidence supporting the author's narrative.

To emphasise this point, the author's own analyses of smokers vs. non-smokers (1a,https://www.biorxiv.org/content/10.1101/2024.09.26.615123v1 [biorxiv.org]) suggests that this will lead to an increase in mutation load). Though there doesn't appear to be a bias for smokers across exposure groups (Fisher's exact test), it is clear that their analyses obfuscate the potential role of this mutational process in these data. While it is important for many aspects relating to quantification of mutational processes, controlling for sample representation will also be important where their representation is uneven, e.g. calculation of clone size; how do I know that the increase in TP53 clone size in CROSS (Fig 2d) isn't driven by a single patient or samples with inadequate sequencing? I especially want it to be clear if the signals for selection, i.e. differences in mutation rate, dn/ds and results in Fig S7 for SPOP, are actually driven by SCC, tissue location or tobacco smoke.

Unlike the Nanoseq analysis, the targeted sequencing was performed over in multiple 2mm^2 samples per patient, to give a more representative insight into the clonal dynamics of the epithelium (**Reviewer Figure 6**). Clone sizes, dN/dS and percentage mutant epithelia are calculated from these multiple samples collected in a gridded array, all of which have an average on target coverage of 250x -800x post duplicate removal allowing detection of clones with a VAF above 1%. We have also verified that there is no association between number of mutations/kb/ gene and the mean coverage of each gene (**Reviewer Figure 7**). The full analysis including the code used is available on https://doi.org/10.5281/zenodo.19627198. We have not included this figure in the revised manuscript at present but would be happy to do so if the reviewer wishes.

We have added the following sentence to the methods:

'We saw no association between number of mutations/kb/gene and the mean gene sequencing coverage (R^2=0.01), see https://doi.org/10.5281/zenodo.19627198 for analysis and code.' Line number 735-736.

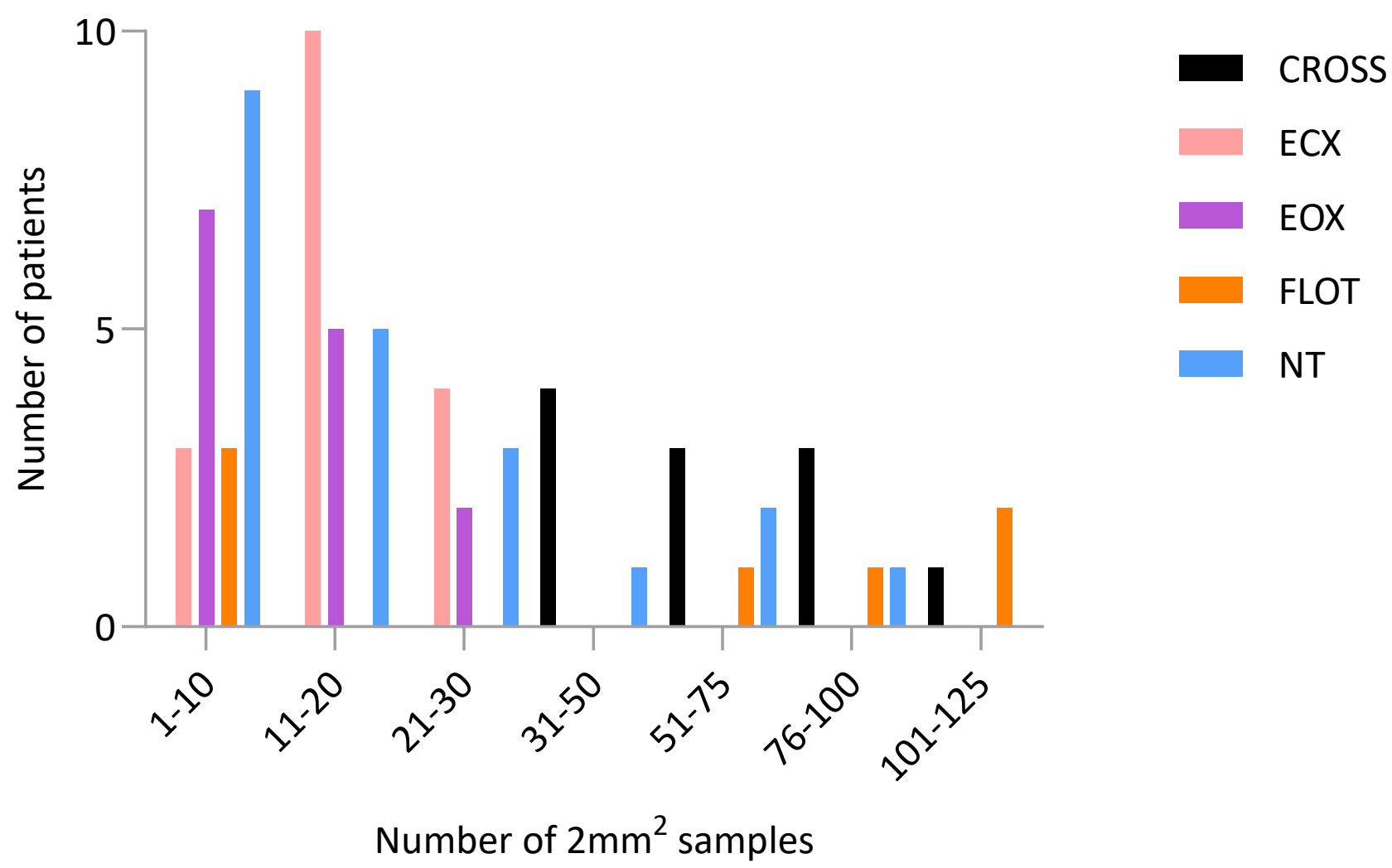

Reviewer Fig. 6: Number of samples per patient (total area of epithelium sampled by targeted sequencing was CROSS, 14.3cm^2, ECX, 5.2cm^2, EOX, 3.26cm^2, FLOT, 8.02cm^2, NT =9.12cm^2).

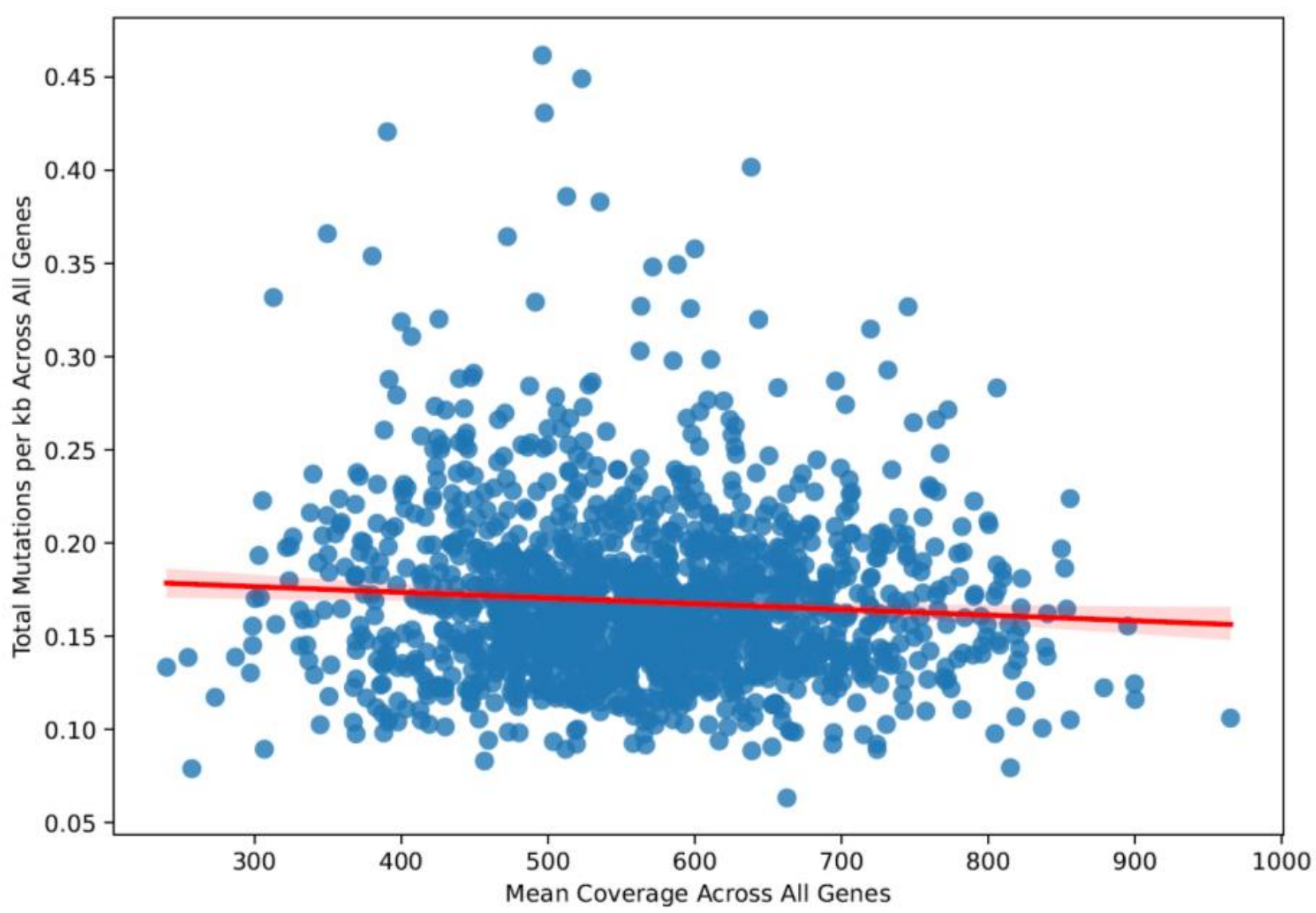

Reviewer figure 7: SBS per Kb plotted against mean coverage across all genes, each dot is a gene. There is no association between the total mutations/ kb/gene and coverage (r^2=0.01).

Our approach of sequencing multiple samples per patient increases the robustness the targeted sequencing results, but it is possible that one or two patients to skew the distribution as the reviewer suggests. Regarding the increase in mutant *TP53* and *PPM1D* in the CROSS group we find this is not the case however (**Reviewer Figure 8**)

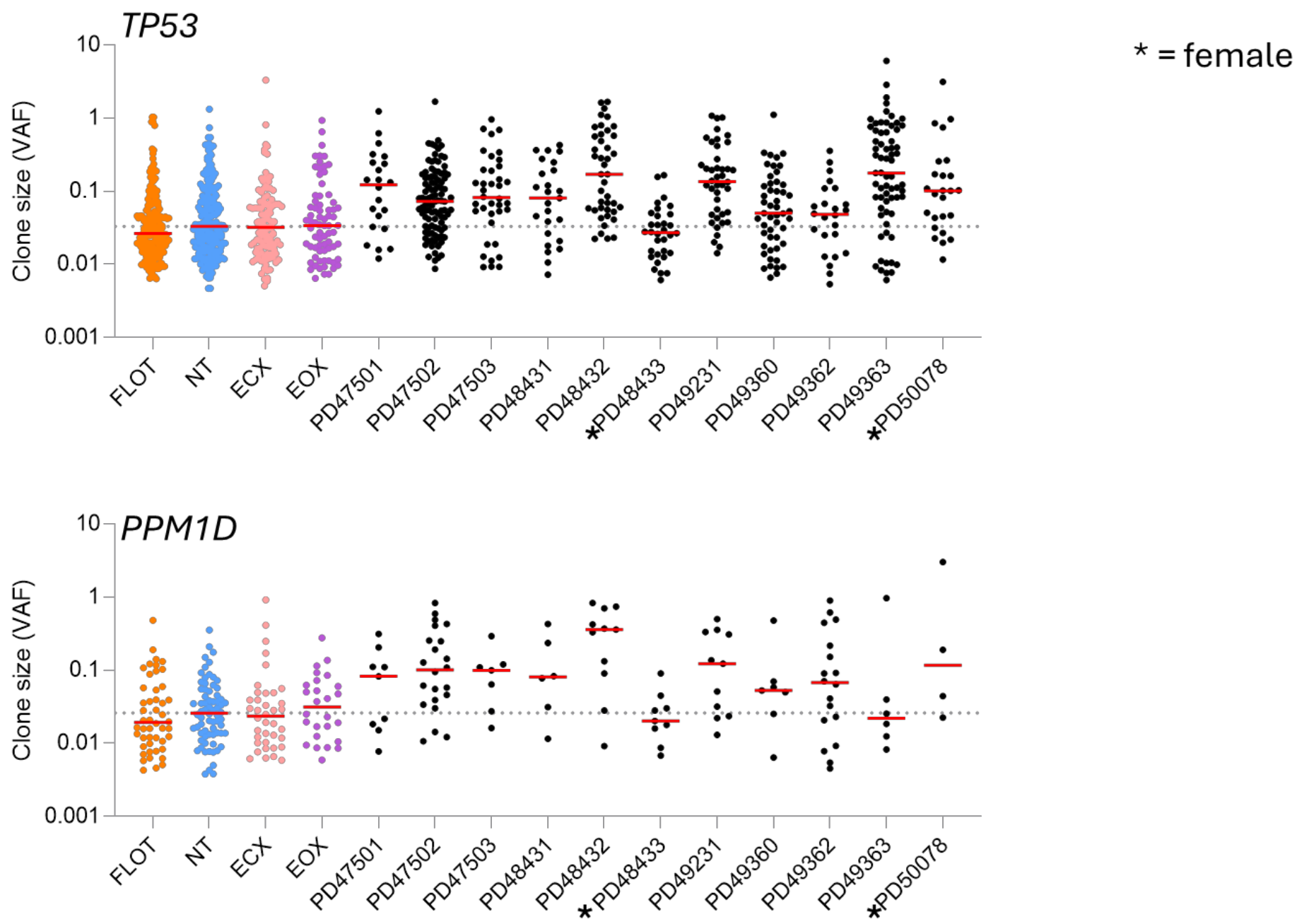

Reviewer Figure 8. Mutant clone sizes for mutant *TP53* and *PPM1D*. Individual values for each CROSS patient are shown with values for all patients in the other treatment groups.

With respect to the clustering analysis, we find no association between Smoking, gender, tumour type, tumour location in the relevant treatment groups (Fisher's exact test) as detailed in the **Reviewer table 3** below and provide further details in the new **Supplementary note**.

	Smoking	Sex	Tumour type (Adeno/SCC)	Tumour location
SPOP MATH vs other (CROSS group)	1	N/A all males	1	1
NFE2L2 KEAP1 binding vs other (FLOT group)	0.27	0.19	N/A all Adeno	N/A all cardias
MTOR Kinase vs other (FLOT group)	0.51	1	N/A all Adeno	N/A all cardias

Reviewer Table 3: Tests for association of missense mutation clustering and the factors shown in CROSS and FLOT patients (Fisher's exact test).

To ensure that the author's statements are robust to the potential impact of confounding variables, I would hope to see some attempts to pull apart the data based upon these variables, such as multivariate regression. For example, this could be applied to the data in Fig S2c and d, where none of the variability between samples and groups is explored with statistical rigor, but is used to state that there is no difference between mutation type in each group.

As the reviewer suggests we have now verified that the proportion of types of mutation (nonsense, splice etc) or SBS, DBS, indel between treatment groups is highly similar (W=0.952, p=0.00077 (nonsense, splice etc), W=1 p=0.007 (SBS, DBS, etc) Kendall's concordance test). We have also performed a more in-depth analysis detailed in the statistical note where we see no association with any type of mutation and treatment, gender, smoking, tumour type, tumour location or age (**Reviewer Table 4,** described in **Supplementary note**).

P_response	ME_treatment	ME_Sex	ME_Smoke	ME_Tumourtype	ME_Tumourlocation	ME_Age
**Nanoseq**						
p_Indel	0.0003	0.9295	1	0.9295	1	0.9295
p_Sub	0.0734	0.3705	0.3705	0.3705	0.7072	0.0734
p_SBS288A	0.905	0.5431	0.8518	0.905	0.905	0.905
p_SBS288B	0.1163	0.7007	0.4527	0.7007	0.329	0.318
**Targeted data**						
p_Mutations	1	1	1	1	0.396	1
p_SBS	0.915	0.915	0.915	1	0.915	0.915
p_DBS	0.25	0.988	0.988	0.723	0.723	0.09
p_Indel	1	1	1	1	0.08	1
p_Nonsyn	1	1	1	1	0.396	1
p_Missense	1	1	1	1	1	1
p_Frameshift	1	1	1	1	0.08	1
p_Splice	0.796	0.796	0.876	0.876	0.796	0.876
p_Nonsense	0.31	0.261	0.785	0.261	0.399	0.387

Reviewer Table 4: Main effects of Treatment and other factors (association of responses and factors): Multiple test corrected p-values of Kruksal Wallis tests except for age where ANCOVA was used.

Similar analyses for individual genes is problematic in its own right, such as is shown in Fig 3C. It is important to bear in mind the inherent biases associated with gene length and codon use. An example is that 43% of the possible mutations over exons 5-8 of TP53 are missense (https://www.nature.com/articles/s41588-024-02039-4 [nature.com]#MOESM4). The authors could instead calculate the log of odds from fishers exact test on each patient and compare against the same gene of bootstrapped mutations randomly sampled from all patients in NT.

We thank the reviewer for this point and have now limited the analysis to comparison on the types of mutations in HLA-B between CROSS and all other treatment groups.

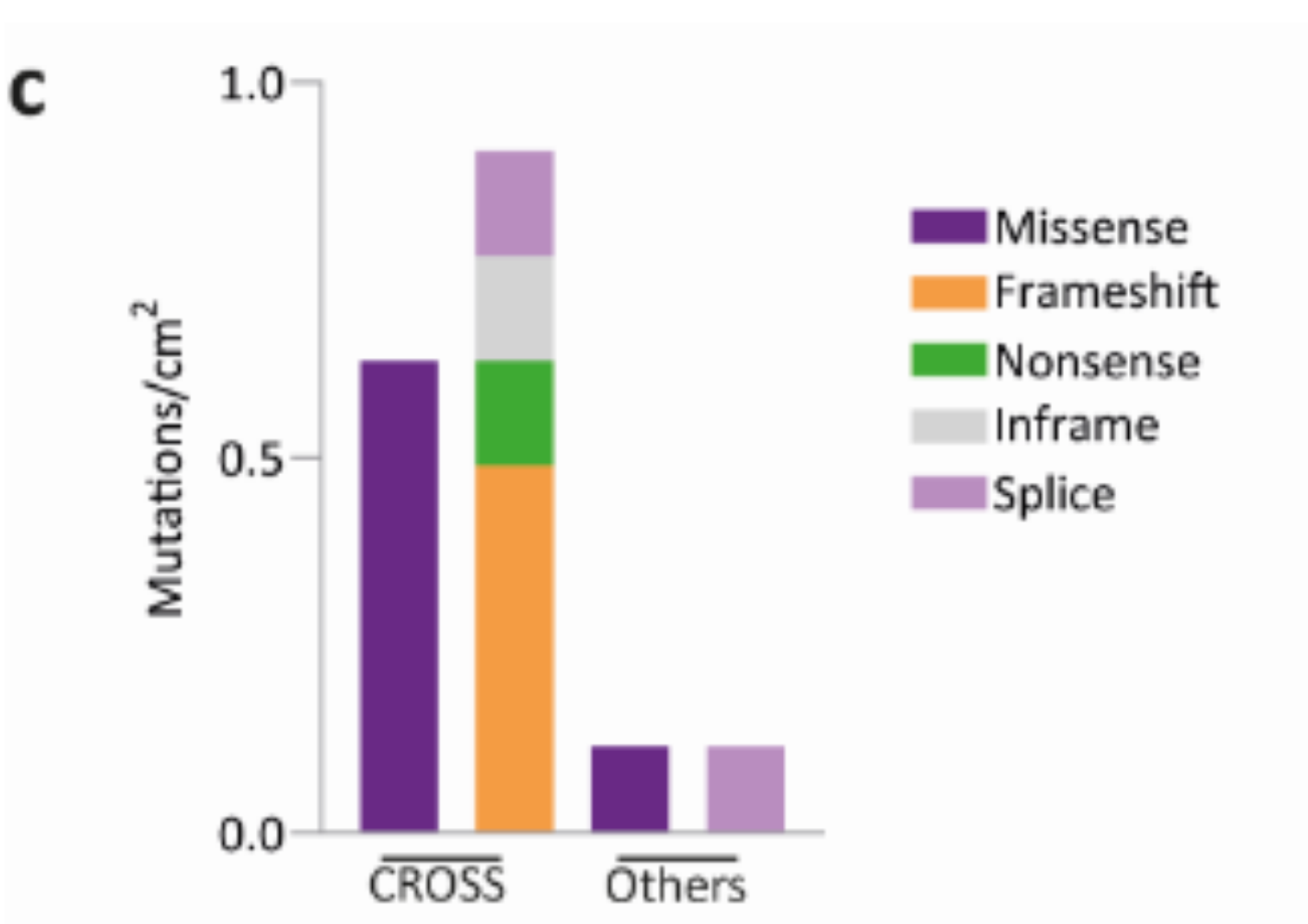

Fig. 4c Selection of HLA mutants. Types of HLA mutations/cm^2 in CROSS patients (CROSS) are significantly different to mutations from all other treatment groups (Others) (p=5.3e-7 Freeman-Halton-Fisher test).

For these data, where clonal inference is so important, it is striking that **mutational processes from nanoseq are assumed to be equally relevant in the panel sequencing data**. I acknowledge that many of these measures will be biased in these data, but the authors should attempt to demonstrate that the clones analysed here are under the same selective pressure through by estimating the mutation rate at synonymous sites as a proxy for the neutral mutation rate (presented in Fig S3d), to show that it correlates with your previous nanoseq data. Mutation signatures would also emphasise that the data are well matched.

We have performed the analysis as suggested by the reviewer and agree with them that substantial caveats should be placed on the comparison. Nanoseq primarily covers be intergenic regions, expected to be under neutral selection. In contrast, the targeted data will comprise relatively few neutral mutations as the genes chosen to be on the bait panel are more frequently under selection.

The SBS/ Mb between the targeted (only synonymous mutations used) and nanoseq are similar but are not well correlated (r2= 0.07, p=0.02, **Reviewer figure 9**). This may reflect the limited number of synonymous mutations in the targeted data. Decomposition signature analysis of the targeted data shows predominantly SBS1 and SBS5 in agreement with the nanoseq results (**Reviewer figure 10**). Some patients show SBS4 (n=5) and SBS36 (n=3) though these sigatures show no correlation with treatment group, smoking status, sex, or tumour type.

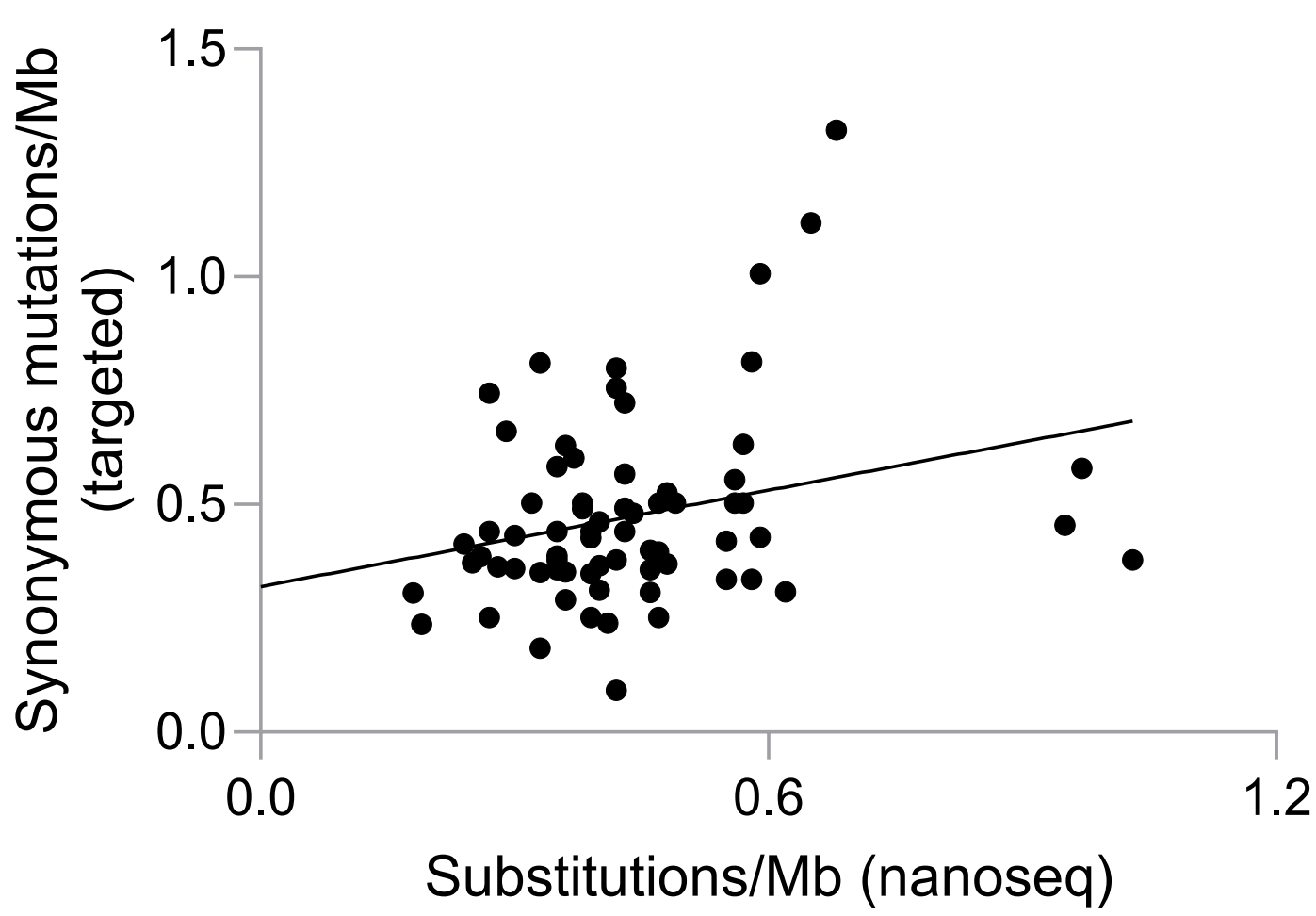

Reviewer figure 9: Relationship between Nanoseq and targeted sequencing burden.

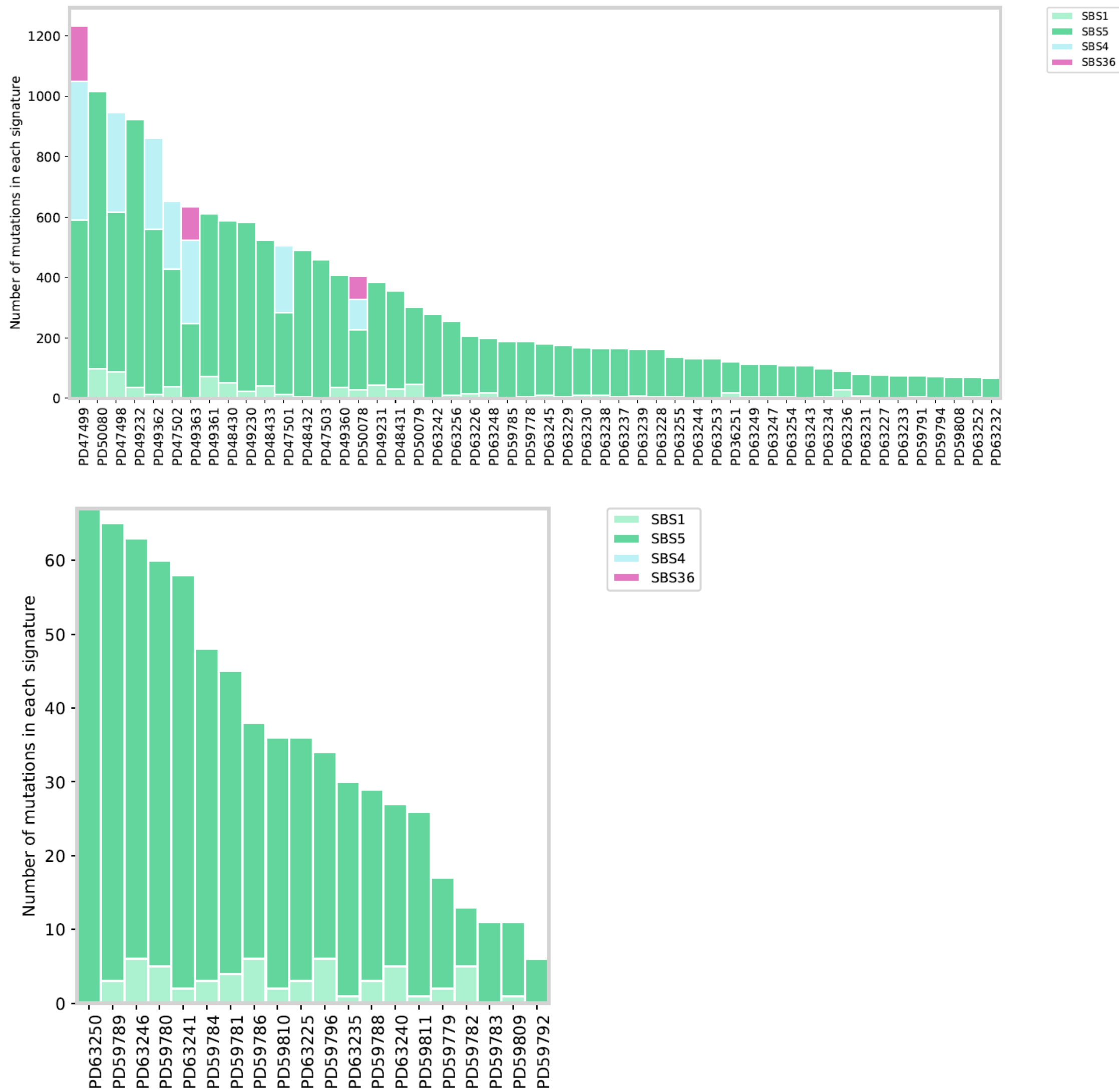

Reviewer figure 10: Deconvolved signatures of targeted sequencing

Overall, we submit that given the differences between the methods and the regions of the genome sequenced likely account for variation between the targeted sequencing and Nanoseq results. Given the uncertainties of this comparison, we have not included this in the revised manuscript at present.

In terms of strengthening the inference of these analyses, especially where smoking is so relevant, **I find it hard to understand why the "UK normal" data weren't included wherever else they're applicable**. This would be really useful to show that smoking has no impact on your analyses.

UK normal has been added to the Nanoseq analysis as suggested by the reviewer. However, as the UK normal bait panel does not cover all the genes analysed in this study and differs in sequencing coverage from this study, we have not included a comparison of UK targeted sequencing data with targeted sequencing this analysis.

3) Inclusion of FLOT and in vitro experiment

As it stands, there's no evidence for therapy-associated clonal outgrowth for FLOT in the same way as the evidence suggests for CROSS. It doesn't fit with the narrative of the work, building that excess mutations from nanoseq signify selection and then disregarding this evidence based upon unique, high dn/ds.

It would make for a nice segue into the section if you could create the same analyses from Fig S4D for NFE2L2, which is uniquely under selection under this regime and relevant to 5FU. A statement about the potential differences in strength of selection or the fact that these clones might need more time than those under CROSS to rise in number and be detectable in your nanoseq analyses would help here.

It's worth mentioning here that the in vitro work from L226 is a nice, complementary experiment, but doesn't tell us if it's actually the combined effect of carboplatin, paclitaxel and radiation in combination as is assessed in vivo. The best you can say without running more explicit experiments is that PPM1D confers resistance to radiotherapy in vitro. for which there appears to be some literature already. There are no statistics for Fig S4D, when there should be.

We agree that the detection of selected of missense kinase mutants in the FLOT group does not result in major clonal expansion as seen with *TP53* and *PPM1D* in CROSS patients. The reviewer's suggestion of a statement commenting on the strength of selection and/or growth rate of the kinase mutants is most helpful and we have included this in the revised text as follows:

'We observed 1D clustering around the KEAP1 binding domain in the FLOT, ECX and NT groups suggesting selection of mutations in this domain occurs in normal oesophagus (**Extended data Fig. 9a**)' Line number 403-405.

Regarding the in vitro experiments, we are currently limited to CRISPR/*Cas9* deletions in the epithelioid system. This enabled us to compare the effect of deletion of *PPM1D* (by targeting exon 1) versus truncation mutants (by targeting exon 6). We agree an important caveat with the system is that we don't model the carboplatin, paclitaxel and radiation combination. Establishing doses of a combination of three agents that correspond to those in vivo is challenging in this primary culture system. We now mention as follows:

'We conclude that radiation selectively expands clones carrying truncating, gain of function (GoF) *PPM1D* mutants in esophageal epithelium although this experiment does not test the carboplatin, paclitaxel and radiation combination given to CROSS patients.' Line number 277-280

Truncating *PPM1D* mutants confer resistance to DNA damaging agents, including radiation and platinum compounds in immortalized cell lines and hematopoetic cells, resulting in the emergence of clonal haematopoiesis in treated cancer patients[38, 39, 40, 41]. However, to our knowledge the increased fitness of *PPM1D* mutant clones has not been demonstrated in primary epithelial cells. We submit that this result in an organotypic esophageal culture does contribute to the paper[42].

Regarding an additional *Nfe2l2* deletion experiment, we have previously shown that *Nfe2l2*$^{-/-}$ cells have decreased fitness relative to wild type cells in esophageal epitheloid cultures (Fig. 7 i,j in [42]). Furthermore, deletion of *Nfe2l2* will not be informative about the impact of the mutations in the KEAP1 binding domains, seen in this study, that increase NFE2L2 levels. We have been exploring prime editing in esophageal epithelioids to model such point mutants over

the last year, finding it challenging, and are unable to add *Nfe2l2* prime editing experiments at present.

Statistics for Fig S4d (now **Fig. 3d** and **e**) are now added. These show significant depletion of cells with exon 1 PPM1D guides compared to control gRNAs under all conditions (**Fig.3d**). However, cells with PPM1D exon 6 guides are only enriched (compared to control gRNA populations) in irradiated cultures (**Fig. 3e**).

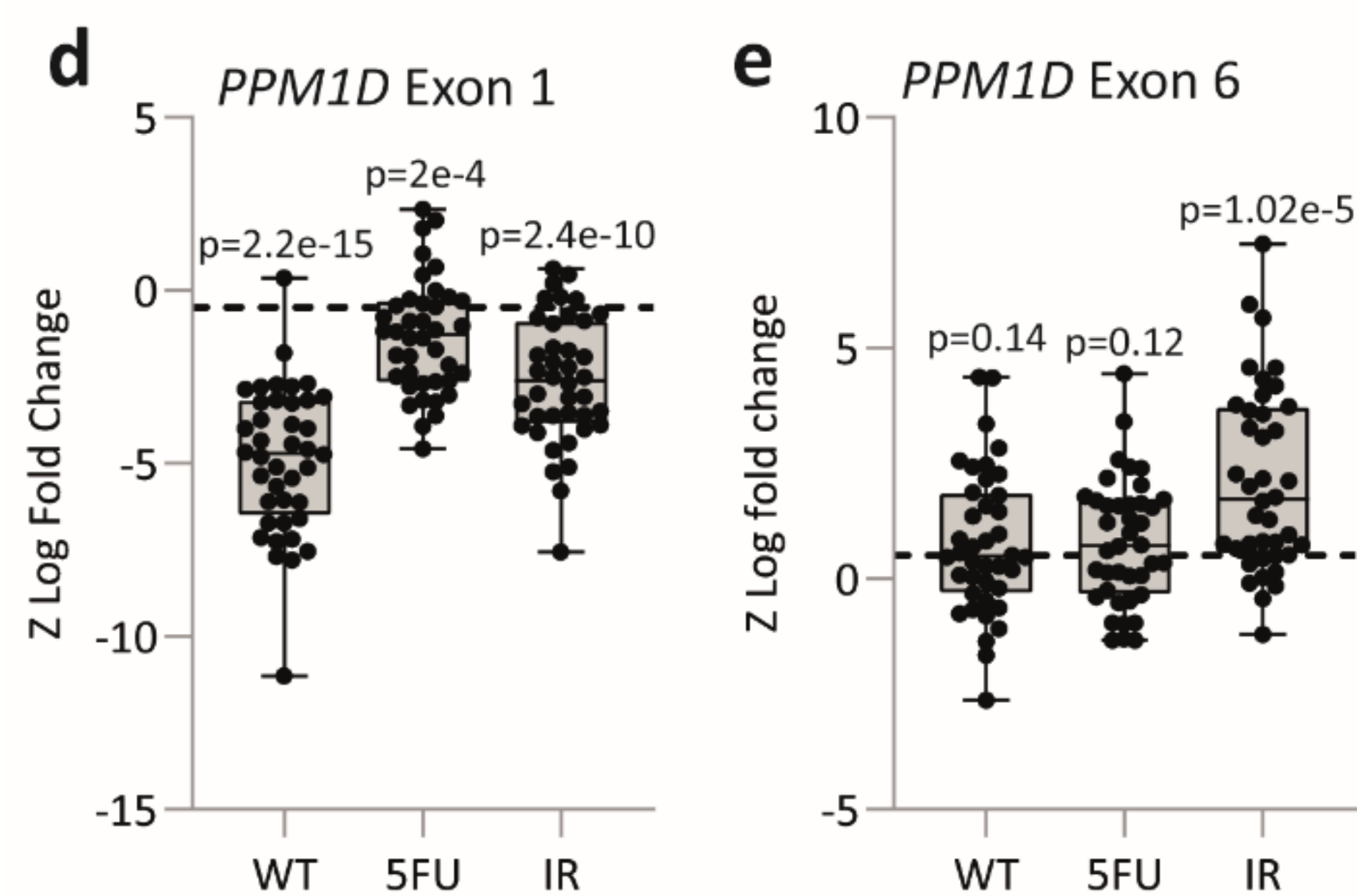

Fig S7d-e Log fold change of gRNA targeting exon 1 (**d**) or exon 6 (**e**) of *PPM1D* at T1 relative to T0. Dots are guide RNAs from four biological replicates, central line shows median, box indicates quartiles and bars indicate minimum and maximum. Thresholds of depleting guides (**d**) and proliferating guides (**e**) are shown by dotted lines and inferred from control guides (methods). P-values determine significance of guides being depleting (**d**) or proliferative (**e**).

=======================================

Minor points

Fig S3B and C looks like the percentage of mutant epithelia decreases with age, as does its variance. That super interesting and it wouldn't take much to formally test this. Fig S1d is also of interest and it would be useful to show that slopes the same for each exposure group, if true.

We have now tested the dependency of the percentage of epithelium mutant for *TP53* and *PPM1D* on age and find no relationship (**Reviewer figure 12**).

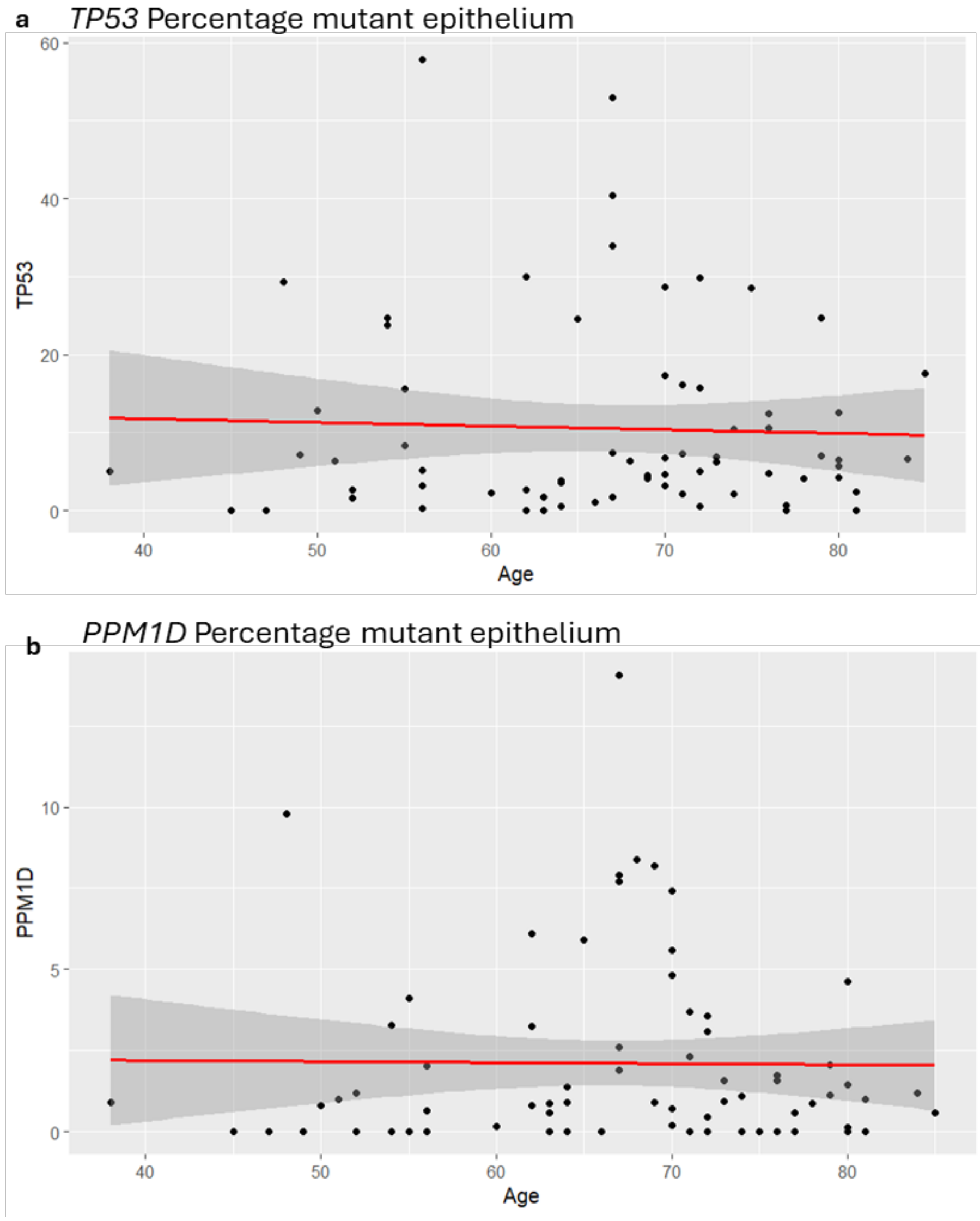

Reviewer figure 12: Association between age and percentage mutant TP53 (**a** adj R2=-0.013, p=0.747) and PPM1D (**b** adj R2=-0.015, p=0.92).

We do not see an association with age and the percentage mutant epithelia for either TP53 or PPM1D (**Reviewer table 1**).

The authors cite Mitchell et al. 2025 (64) to show that signatures for chemotherapy are found in duplex sequencing. I would add other work by Nuria Lopez Bigas, Sam Behjati and Jyoti Nangalia, Ellie Dunstone, who have all demonstrated that similar signals are prevalent and to drive that these findings are unusual.

Thank you for highlighting these highly relevant papers in tissues other than the esophagus. We now reference these as follows:

"The absence of a chemotherapy mutational signature in both Nanoseq and whole genome samples of normal epithelium is notable. In contrast, in other normal tissues patients treated with chemotherapy, drug induced mutations have been detected with duplex sequencing approaches as used in this study[26, 27, 28, 43]. The mutational signatures observed in other tissues include those from platinum compounds, 5-FU and Capecitabine, all of which were included in the treatment protocols used here[22, 23, 27, 44]. The lack of detectable mutational signatures in esophagus from treated individuals is unusual and may reflect loss of epithelial cells that acquire sufficient chemotherapy-induced damage by cell death or differentiation and shedding." Line number 544-551

Fig S2a Is there no cut off for poorly/excessively sequenced samples? If there is QC for inclusion, please state any thresholds or tests.

We now include the QC thresholds for coverage in Extended data Fig. 5a. These were a minimum of 250 and maximum of 800 reads after removal of duplicates.

L258 How were these samples selected? Sample numbers are very biased towards CROSS samples, so it would be good to know upfront the sample numbers. It's a shame that the FLOT and NT samples originate from only two patients, but it should be stated clearly.

We now include the criteria for sequencing. All samples with a mutation with a VAF greater than 0.35 were whole genome sequenced.

Text now reads:

Only samples which had a mutation with VAF>0.35 were selected for WGS. Line number 310

==

Formatting etc

Fig 3e In the block for sample 3, there are two unjoined groups of squares- what does that represent?
These are pieces of tissue from the same region which are not physically joined. We now explain this in the legend as follows: 'The spatial location of adjacent 2mm^2 samples is shown, unjoined groups of samples were from the same area but were not physically joined'. Line number 333-334

L89 I don't think these papers need to be cited with respect to a statement about your own results.

These references have been removed.

L261 Please include a reference for "Loss of heterozygosity (LOH) of the NOTCH1 gene were seen as expected"

We have now added references

'Loss of heterozygosity (LOH) of the *NOTCH1* gene was seen, an event which is also common in normal epithelium from patients without cancer[1, 4, 45].' Line number 313-314

L167 This sentence is redundant and should be removed.

This has been done.

Table s3 sample PD59796 is missing

The germline sample from this patient failed the Nanoseq library preparation and there was insufficient remaining DNA to repeat it. This has now been stated in the methods. Line number 770-771

L59 It isn't obvious that the combinations of chemotherapeutics associated with each regime is detailed in Table S1 and their administration in Fig 1b. The description of the treatments also isn't exactly representative of FLOT, which is a platinum agent, a taxane AND an antimetabolite with folonic acid. The description of drug combinations. Could be expressly written in full on 1b and make up for the white space of 1a, which could be stacked.

Thank you for this helpful suggestion, we have revised the figure accordingly.

L641 a non synonymous mutations with VAF>0.35 // one of these is wrong: XaX non or synonymous mutationXsX

We have corrected this to: ‘a non-synonymous mutation with VAF>0.35’ Line number 760-761

L153 This is turn suggests that …

Corrected to: This in turn suggests that Line 190

L67 “excised” d

Corrected Line number 68
======================================

Reviewer #2 (Remarks on code availability):

Data availability and processing:

Data under EGA accessions are unavailable as of 11.12.25
Data is now available with EGA

I decided to follow the notebook in ATM, as this gene isn’t mentioned in the manuscript and not considered among any of the genes under selection.

Following their instructions, I installed a conda environment for the darwinian_shift package and downloaded the human genome reference data required from the earlier version of this package. I had to manually call the genome and annotation with the reference_fasta and exon_file options, where the authors called the source_genome option. I needed to install another dependency to open the external data: pip install openpyxl. I was able to run all the code and reproduce all the figures but the last, which was very different. It is not clear to me

how this plot fits in with those of the rest of the manuscript- the plot is included in the attached PDF.

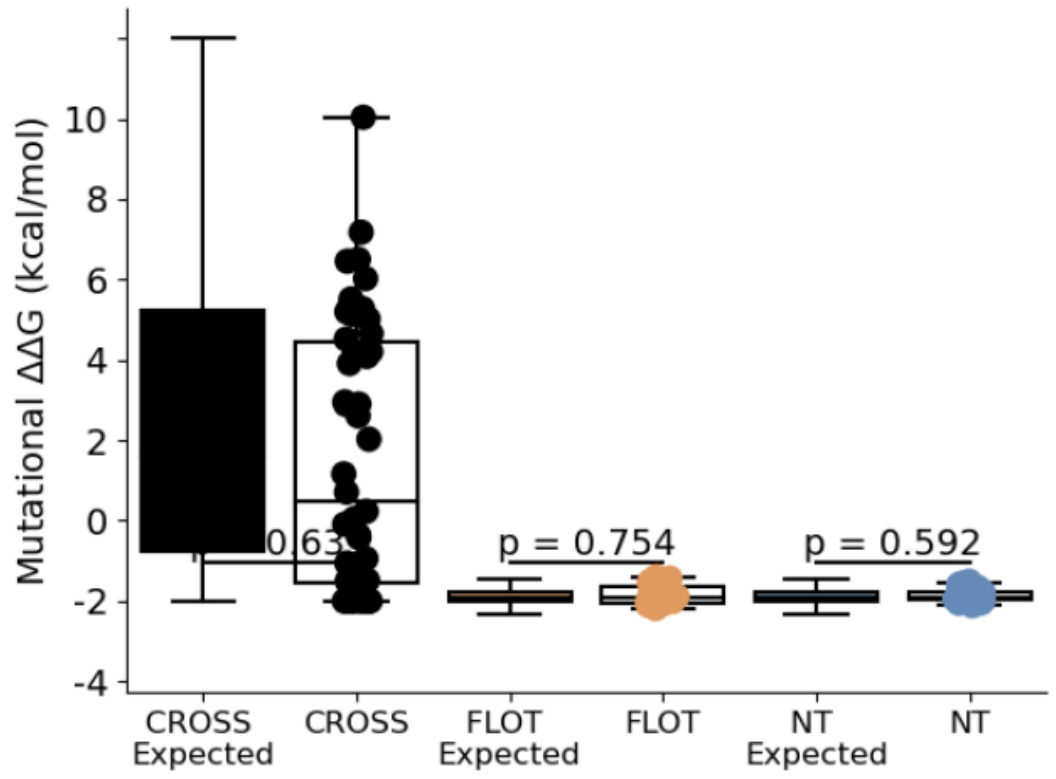

This graph was from a previous version of the paper, we have removed it and tidied up the Github respository.

On code for data processing not included in the github, suitable github repositories are provided in the methods section.

On the github link provided for this work, there exist the following folders:
ATM - jupiter notebook as other genes
Foldx - nothing to run
Foldx_binding - nothing to run
MTOR - jupiter notebook as other genes
RAC1 - jupiter notebook as other genes
SPOP - jupiter notebook as other genes
clustering - nothing to run

Reviewer #3 (Remarks to the Author):

This manuscript examines how neoadjuvant chemotherapy and chemoradiation shape somatic clonal selection in histologically normal human oesophageal epithelium. By combining high-depth targeted sequencing with NanoSeq, the authors show that treatment does not substantially increase mutation burden but instead selectively expands pre-existing mutant clones in a treatment-specific manner. In particular, TP53 and PPM1D mutant clones are enriched following chemoradiation, while mutations in genes such as MTOR, RAC1, and NFE2L2 are preferentially selected in patients receiving FLOT chemotherapy.

My comments focus primarily on the protein- and structure-level interpretation of missense mutation selection, including the use of structural clustering, molecular modelling, and ΔΔG-based analyses to infer functional consequences of selected variants.

We thank Reviewer 3 for their constructive and detailed assessment, particularly regarding the structural and protein-level interpretation of missense selection. We agree that clearer methodological description, more quantitative reporting, and more cautious wording around functional inference will strengthen the manuscript. In the revised version we (i) clarify which

structural datasets are used and why, (ii) define each 3D analysis with explicit metrics and statistical testing, (iii) expand reporting of gene-level selection statistics, and (iv) moderate mechanistic claims to distinguish baseline selection from treatment-associated quantitative shifts.

General methodological and reporting issues

• The manuscript refers to "genes for which structural information was available", but it is unclear how this was defined. Given the widespread availability of high-quality predicted structures, clarification is needed as to whether this refers specifically to experimentally determined PDB structures, ligand-bound conformations, or other criteria. This distinction is important for understanding the scope and potential biases of the 3D clustering and ΔΔG analyses.

We agree this requires clarification. In the revised manuscript we explicitly define “structural information” as experimentally determined structures deposited in the Protein Data Bank (PDB), including apo and ligand-bound conformations where available. We did not use predicted structures (e.g., AlphaFold) for the primary 3D clustering or ΔΔG analyses reported here.

This choice was made to ensure (i) residue-level coordinates reflect experimentally observed conformational states, and (ii) ligand / interface geometries are grounded in empirical complexes when interpreting proximity-to-ligand and binding calculations. We now state this explicitly in the Methods and note in the Discussion that extending analyses to high-confidence predicted structures is an important future direction.

‘ This was augmented by 3D clustering analysis for genes where experimentally determined structures deposited in the Protein Data Bank including apo and ligand-bound conformation were available’. Line number 209-211

And

The significance of missense mutations can be further explored by extending the analysis to high confidence predicted protein structures. Line number 593-595

• The 1D clustering analysis using NMC is reasonably well defined. However, the manuscript refers to "**3D clustering" without clearly specifying a single metric or statistical framework**. It would be helpful to clarify how 3D clustering is defined, including how distances are computed, what constitutes a cluster versus enrichment, and how significance is assessed and corrected for multiple testing. In addition, clustering is generally reported in binary terms (significant vs non-significant), which makes it difficult to compare the strength of clustering across genes and treatment groups. Providing quantitative measures of clustering or enrichment (for example, observed versus expected mutation density within predefined functional regions, or effect sizes with confidence intervals) would allow more direct comparison between conditions and help distinguish baseline selection from treatment-specific effects. As currently presented, multiple conceptually distinct analyses are grouped under the term "3D clustering", limiting assessment of robustness and reproducibility.

We agree that the term “3D clustering” was used too broadly and that the underlying analyses should be more clearly defined and quantitatively reported. In the revised manuscript, we more

clearly and explicitly define structural enrichment tests, each with a stated metric, null model, and statistical procedure.

For each test, we state exactly how distances are computed (Cα–ligand heavy atom minimum distance / nearest-atom distance, etc.), how the expected distribution is generated (Monte Carlo sampling conditional on mutational context), and how significance is assessed. Finally, we revise the text throughout to avoid binary "clustered/not clustered" language and instead describe results as quantitative shifts relative to baseline selection.

• The ΔΔG-binding analyses rely heavily on FoldX-based scoring. FoldX was originally developed for protein stability estimation and is not well validated for ligand binding, particularly for non-standard ligands. The manuscript would benefit from clarification of ligand parameterisation, benchmarking against known effects, and more cautious interpretation of binding energy changes.

We agree with the reviewer that FoldX was originally developed for stability estimation and that ligand-binding ΔΔG predictions are more uncertain, particularly for non-standard ligands. In the revised manuscript we therefore expand the Methods to describe ligand parameterisation in FoldX via pyFoldX (in the same way as our previous publication: https://doi.org/10.1016/j.ymeth.2023.12.008), and explicitly state the intended use and limitations of these calculations.

Importantly, we use ΔΔG estimates comparatively and distributionally, not as absolute physical binding free energies: we test whether the observed mutation set is shifted relative to a null distribution matched to the treatment group's mutational spectrum.

We have moderated the language throughout to frame these results as supportive and hypothesis-generating, and we now avoid statements implying direct experimental binding changes without validation. We also add parallel stability (ΔΔGfold) estimates where relevant to avoid over-focusing on binding as the sole mechanism.

• The dN/dS analyses appear to be performed across the full targeted gene panel, but only a small subset of genes are reported in the supplementary tables. Providing complete gene-level results (including non-significant genes), or at least ranked summaries per treatment group, would greatly aid interpretation and assessment of the specificity and relative strength of the reported selection signals.

We agree and thank the reviewer for highlighting this reporting gap. In the revised Supplementary Information we now provide complete gene-level dN/dS results for the full targeted panel for each treatment group, including non-significant genes, together with q-values and relevant model outputs. This is now reported in Extended data Tables 8-12.

Gene-specific interpretation of missense selection
-SPOP
• **The interpretation of selected SPOP missense mutations as loss-of-function under chemoradiation would benefit from clearer definition and stronger calibration.** While prior literature supports classification of prostate cancer SPOP mutations as loss-of-function, the manuscript does not show whether these reference mutations exhibit comparable 1D or 3D clustering patterns to the CROSS-selected variants. Demonstrating similar spatial

clustering would strengthen the inference that the two sets share a common functional mechanism.

We agree that the inference benefits from calibration. In the revised manuscript we explicitly compare CROSS-selected SPOP missense mutations with prostate cancer-associated SPOP variants previously characterised as loss-of-function, using both 1D positional enrichment and 3D proximity-to-interface analyses. We now show that CROSS mutations are biased toward the same functional surface as the prostate cancer set, although the magnitude of enrichment is modest and overlaps with baseline selection observed in untreated samples. Accordingly, we have revised the interpretation to emphasise interface-focused baseline selection with a treatment-associated quantitative shift, rather than asserting chemoradiation-specific loss-of-function as a definitive conclusion.

• In addition, the ΔΔG analyses focus exclusively on ligand-binding energetics. Loss of function in adaptor proteins such as SPOP is often mediated by effects on folding stability, abundance, or regulation rather than selective disruption of a single interaction. Assessing predicted stability effects, or clarifying why ligand binding is expected to be the dominant mechanism in this context, would help justify the functional interpretation.

We agree that loss of function in adaptor proteins such as SPOP may arise from effects on folding stability, abundance, or regulation rather than selective disruption of a single interaction. We now include predicted protein stability ΔΔG estimates alongside binding-related analyses and clarify that multiple mechanisms may contribute in text. These findings demonstrate that no strong effect is observed in the folding contributions to SPOP, and that the primary observed difference is actually binding ΔΔG.

Text now reads:

'Multiple mechanisms can be responsible for loss of function aside from changes in ligand binding energies; these include protein abundance, and folding stability. We investigated the predicted stability of SPOP proteins carrying mutations seen in CROSS patients and found that no strong effect is observed in the folding contribution. Our analysis finds ΔΔG is the primary observed difference between CROSS and NT groups (Extended data Fig. 8c).' Line number 387-391

• Finally, spatial clustering of missense mutations supports functional relevance but does not distinguish loss- from gain-of-function, as clustering has frequently been associated with activating mutations in cancer. It is also notable that significant clustering relative to the ligand-binding site is observed in the no-treatment group. This suggests that clustering reflects baseline selection on SPOP rather than a chemoradiation-specific effect, and therefore does not, by itself, support a treatment-specific loss-of-function mechanism. Notably, in Fig. 3g the distribution of predicted binding energy changes for CROSS-selected mutations appears closer to the neutral distribution than to the prostate cancer loss-of-function set, suggesting that effects may be modest or heterogeneous. A more cautious framing of the conclusions, or additional quantitative comparisons, would therefore be appropriate.

We agree that spatial clustering alone does not distinguish loss- from gain-of-function, and that the presence of significant interface enrichment in the no-treatment group indicates baseline selection on SPOP. We argue however that this enrichment is significantly enhanced

in the CROSS group, and that therefore it is likely that exposure to CROSS treatment increases selection of SPOP activating mutations compared to NT.

Regarding the observation that SPOP CROSS selected mutations appear closer to the expected distribution than to the prostate cancer selected data, we agree that whilst this appears true – the expected distribution is generated via 10,000 permutations of expected locations, and so the high energy mutations observed are indeed rare. In addition – statistical analysis indicates a significant different between CROSS expected and CROSS observed (p = 0.006), and no statistically significant difference to the Prostate cancer mutations (p = 0.236), we therefore believe it is justifiable to say that the CROSS observed mutations are more similar to the Prostate than expected. We do agree that more cautious framing of the results would aid in interpretation here, so we have tempered the language throughout.

Text now reads:

'SPOP mutations in CROSS patients show similar 1D positional enrichment, 3D proximity to interface and ligand binding energy to the LoF mutations seen in prostate cancer (**Extended data Fig. 8d, Fig. 4g**). We hypothesise that LoF missense SPOP mutants may be quantitatively selected by chemoradiation. Line number 391-394

-NFE2L2

• The NFE2L2 analysis is biologically plausible and broadly consistent with prior work on KEAP1-NFE2L2 regulation. However, clustering of missense mutations within the KEAP1-binding region is also observed in ECX and no-treatment groups, indicating baseline selection on this interface. The treatment-specific effect therefore appears quantitative rather than qualitative, and this distinction would benefit from clearer emphasis.

We agree – this observed difference is in magnitude and suggests an increased selective pressure compared to no treatment and ECX group. In the revised manuscript we explicitly state that enrichment of missense mutations within the KEAP1-binding motifs is observed in untreated and ECX samples, consistent with baseline selection on the KEAP1–NFE2L2 regulatory interface in normal oesophagus. The FLOT group shows the strongest quantitative enrichment (higher fraction within the motif and higher mutation density/clone representation), and we now present this as a quantitative shift under treatment, rather than a qualitatively new pattern unique to FLOT.

Text now reads:

'We observed 1D clustering around the KEAP1 binding domain in the FLOT, ECX and NT groups suggestion selection of mutations in this domain occurs in normal oesophagus (**Extended data Fig. 9a**).' Line number 403-405.

And

'The FLOT group showed the most significant clustering ((**Extended data Fig. 9a**) with the highest density of mutations/cm^2 (**Extended data Fig. 9c**) and 78% of the missense mutations occurring within the exDLG-ETGE KEAP1 binding region (amino acids 17-82, Fig. S11d), though this does not reach statistical significance. We suggest that although mutations in NFE2L2 may be selected in normal oesophageal epithelium this is further enhanced by FLOT chemotherapy' Line number 412-407.

• In addition, the conclusion that selected NFE2L2 mutations are protein-stabilising and confer chemoresistance is inferred from positional clustering and prior literature rather than

demonstrated directly in this system. Incorporating predicted ΔΔG effects on protein stability for the observed missense variants, or otherwise quantifying their likely impact on NFE2L2 stability or abundance, would help support the proposed mechanism and distinguish stabilising regulatory effects from alternative functional consequences. Clarifying this point, or moderating the language accordingly, would strengthen this section.

We agree that the stabilisation/chemoresistance link was inferential as written. Unfortunately, we don't have extensive structural information for NFE2L2 presently (there is an Alphafold predicted structure but it is not biologically meaningful). The main structural information present for NFE2L2 is a small section (residues 71 to 87) bound to KEAP1. This is one of our predicted hotspots – and so we have performed binding ΔΔG analysis of this small section of the protein – finding that mutations observed in cancer consistently increase the predicted binding energy and therefore are predicted to decrease the likelihood of binding and act a loss of function. We note however that given the small section of protein resolved in the structure we cannot generate basal distributions in order to statistically test this hypothesis. We have revised the text to reflect that our data support selection for KEAP1-interface disruption, consistent with prior reports linking such mutations to increased NRF2 signalling and drug tolerance, but we avoid implying demonstrated chemoresistance in this cohort.

Text now reads

'Nearly all mutations observed across the cohort showed a predicted increase in ligand binding energy therefore decreasing the likelihood of KEAP1 binding (**Extended data Fig. 9b**).' Line number 407-409.

-RAC1

• The RAC1 analysis integrates clustering, structural proximity metrics, ΔΔG-binding predictions, and MD simulations to infer selection for activating mutations under FLOT. While the biological framework is plausible, several aspects would benefit from clarification. Notably, enrichment of mutations in hinge residues is observed in both FLOT and no-treatment groups, indicating baseline selection on RAC1 rather than a treatment-specific effect. The FLOT-specific conclusion therefore rests primarily on differences in proximity to GTP and predicted binding energetics, for which effect sizes and biological significance are not fully established.

We agree that hinge-region enrichment in both FLOT and untreated samples indicates baseline selection acting on RAC1, and that the treatment-associated signal is primarily quantitative. In the revised manuscript we therefore separate (i) baseline hinge enrichment (shared across groups) from (ii) the FLOT-associated shift toward mutations closer to the nucleotide-binding pocket and toward a distributional shift in predicted nucleotide-binding energetics. We have revised the conclusions to avoid implying that increased GTP affinity alone is the definitive activating mechanism.

Text now reads:

'Both FLOT and NT groups show significantly increased numbers of mutations within the hinge residues compared to an expected Monte Carlo sampled distribution based on the mutational spectrum of each treatment group (**Fig. 6b**). These two groups also show significantly closer mutational distance to the Switch domains compared to an expected distribution based on the mutational signature (**Extended data Fig.10c**). This suggests selection of mutations within these regions occurs in untreated oesophageal epithelium. Line number 439-444.

• In addition, RAC1 functions as a molecular switch, and activating mutations need not act by increasing GTP affinity alone. Many oncogenic GTPase mutations alter conformational cycling, impair GAP-mediated hydrolysis, or modify effector interactions, often through effects on Switch I or Switch II regions. Clarifying whether the identified hinge mutations overlap these functionally critical regions, rather than relying primarily on proximity to bound GTP, would strengthen the mechanistic interpretation.

We agree and have revised the mechanistic interpretation accordingly. In the revised text we explicitly map observed variants onto established functional regions of RAC1- Switch I and Switch II, and we clarify that oncogenic activation can arise through altered conformational cycling, impaired hydrolysis, or altered effector engagement, not solely nucleotide affinity. We now present proximity-to-GTP and ΔΔG calculations as one line of supportive evidence combined with distance to Switch I and Switch II, rather than the primary mechanistic determinant.

Text now reads:

'We suggest that untreated epithelia harbors a pool of *RAC1* mutations involving the hinge region, but that treatment with FLOT selects those which act through increased affinity of GTP for the binding site, though activating mechanisms involving altered conformational cycling, impaired hydrolysis or altered effector engagement are also possible.' Line number 455-459

• The MD simulations are performed for a small, selected set of mutations, including known gain-of-function variants, and no comparison is shown for mutations observed in untreated samples. As presented, these simulations support plausibility but do not independently distinguish treatment-specific activating effects. A more cautious framing of the conclusions, or additional quantitative comparison across groups, would strengthen this section.

We now frame the MD simulations as illustrating plausibility of altered switch dynamics for selected variants rather than as evidence distinguishing treatment-specific activation. We have revised the text to emphasise that the MD results are consistent with altered flexibility in switch regions for specific variants, but do not, on their own, establish treatment-specific activating selection.
The text now reads

'These results are consistent with the hypothesis that mutations within the Switch regions can increase the flexibility of the hinge region to increase activity' Line number 455-456

-MTOR
• The MTOR analysis is compelling and benefits from strong prior knowledge of kinase-domain gain-of-function mutations and their association with increased clonal fitness. The enrichment of kinase-domain mutations, their overlap with known activating variants, and their association with larger clone sizes together provide convincing support for selection of activating MTOR mutations, particularly under FLOT treatment.

• However, activation of MTOR can arise through allosteric relief of autoinhibition as well as direct effects on the kinase domain. Clarifying whether the selected mutations map to regions associated with allosteric regulation or structural unlocking would further strengthen the

interpretation of these variants as activating, and may help explain why pathway activation is observed without predicted disruption of rapamycin binding.

We agree and have strengthened this section. Mutations are observed in regions involved in autoinhibition and allosteric relief in addition to kinase-domain localisation. We add corresponding discussion to note that activating mTOR variants can arise through both direct kinase-site effects and allosteric relief of autoinhibition, and we interpret our positional enrichment accordingly.

Text now reads:

'A significant proportion of *MTOR* mutations in all treatment groups overlap with known, experimentally validated GoF mutations (**Fig. 7d**). These activating mutations can arise though both direct kinase-site effects and allosteric relief of autoinhibition' Line number 491-494.

• The subsequent interpretation regarding druggability and resistance would also benefit from clarification. Predicted lack of effect on rapamycin binding does not directly imply pathway sensitivity or resistance in vivo, and the conclusion that "druggable GoF MTOR mutants confer resistance" appears broader than what is supported by the presented data. A more cautious framing distinguishing selection under chemotherapy from therapeutic resistance, or clarification of the specific form of resistance being implied, would strengthen this section.

We agree that our wording overstated what the binding calculations support. Predicted lack of effect on rapamycin binding does not demonstrate in vivo sensitivity or resistance, and our study does not directly test pharmacological response. We have therefore revised this text to distinguish selection under chemotherapy from therapeutic resistance to mTOR inhibitors. In the revised manuscript we state that many selected mTOR variants overlap known gain-of-function mutations and are potentially targetable, and that our calculations do not suggest obvious loss of rapamycin binding for the observed variants; however, we explicitly frame this as hypothesis-generating and avoid claims of clinical resistance or sensitivity without experimental validation.

The sentence 'We conclude that druggable GoF *MTOR* mutants confer resistance in FLOT patients' has therefore been removed from the manuscript.

References

1. King C*, et al.* Somatic mutant selection is altered by prior NOTCH1 mutation in aging esophageal epithelium. *bioRxiv*, 2024.2009.2026.615123 (2026).

2. Calvet F*, et al.* Sex and smoking bias in the selection of somatic mutations in human bladder. *Nature* **647**, 436-444 (2025).

3. Lawson ARJ*, et al.* Somatic mutation and selection at population scale. *Nature* **647**, 411-420 (2025).

4. Martincorena I*, et al.* Somatic mutant clones colonize the human esophagus with age. *Science (New York, NY)* **362**, 911-917 (2018).

5. Yokoyama A*, et al.* Age-related remodelling of oesophageal epithelia by mutated cancer drivers. *Nature* **565**, 312-317 (2019).

6. Chang J*, et al.* Genomic analysis of oesophageal squamous-cell carcinoma identifies alcohol drinking-related mutation signature and genomic alterations. *Nature Communications* **8**, 15290 (2017).

7. Skrupskelyte G*, et al.* The dynamics of mutational selection in cutaneous squamous carcinogenesis. *Commun Biol*, (2026).

8. Fowler JC*, et al.* Selection of Oncogenic Mutant Clones in Normal Human Skin Varies with Body Site. *Cancer Discovery* **11**, 340-361 (2021).

9. Hanley JA, Lippman-Hand A. If nothing goes wrong, is everything all right? Interpreting zero numerators. *Jama* **249**, 1743-1745 (1983).

10. Rahman S, Kosakovsky Pond SL, Webb A, Hey J. Weak selection on synonymous codons substantially inflates <i>dN/dS</i> estimates in bacteria. *Proceedings of the National Academy of Sciences* **118**, e2023575118 (2021).

11. Albu M, Min XJ, Hickey D, Golding B. Uncorrected Nucleotide Bias in mtDNA Can Mimic the Effects of Positive Darwinian Selection. *Molecular Biology and Evolution* **25**, 2521-2524 (2008).

12. Peters GJ*, et al.* Induction of thymidylate synthase as a 5-fluorouracil resistance mechanism. *Biochimica et Biophysica Acta (BBA)-Molecular Basis of Disease* **1587**, 194-205 (2002).

13. Schmitt M*, et al.* Colon tumour cell death causes mTOR dependence by paracrine P2X4 stimulation. *Nature* **612**, 347-353 (2022).

14. Jiang X*, et al.* High expression of nuclear NRF2 combined with NFE2L2 alterations predicts poor prognosis in esophageal squamous cell carcinoma patients. *Mod Pathol* **35**, 929-937 (2022).

15. Akhdar H, Loyer P, Rauch C, Corlu A, Guillouzo A, Morel F. Involvement of Nrf2 activation in resistance to 5-fluorouracil in human colon cancer HT-29 cells. *European Journal of Cancer* **45**, 2219-2227 (2009).

16. Kim EJ, Kim YJ, Lee HI, Jeong S-H, Nam HJ, Cho JH. NRF2 Knockdown Resensitizes 5-Fluorouracil-Resistant Pancreatic Cancer Cells by Suppressing HO-1 and ABCG2 Expression. *International Journal of Molecular Sciences* **21**, 4646 (2020).

17. Goka ET, Chaturvedi P, Lopez DTM, Garza ADL, Lippman ME. RAC1b Overexpression Confers Resistance to Chemotherapy Treatment in Colorectal Cancer. *Molecular Cancer Therapeutics* **18**, 957-968 (2019).

18. Ye J, Pavlicek A, Lunney EA, Rejto PA, Teng CH. Statistical method on nonrandom clustering with application to somatic mutations in cancer. *BMC Bioinformatics* **11**, 11 (2010).

19. Delgado J, Radusky LG, Cianferoni D, Serrano L. FoldX 5.0: working with RNA, small molecules and a new graphical interface. *Bioinformatics* **35**, 4168-4169 (2019).

20. Radusky LG, Serrano L. pyFoldX: enabling biomolecular analysis and engineering along structural ensembles. *Bioinformatics* **38**, 2353-2355 (2022).

21. Jamshidi Parvar S, Hall BA, Shorthouse D. Interpreting the effect of mutations to protein binding sites from large-scale genomic screens. *Methods* **222**, 122-132 (2024).

22. Pich O, Muiños F, Lolkema MP, Steeghs N, Gonzalez-Perez A, Lopez-Bigas N. The mutational footprints of cancer therapies. *Nat Genet* **51**, 1732-1740 (2019).

23. Christensen S*, et al.* 5-Fluorouracil treatment induces characteristic T>G mutations in human cancer. *Nat Commun* **10**, 4571 (2019).

24. Alexandrov LB*, et al.* The repertoire of mutational signatures in human cancer. *Nature* **578**, 94-101 (2020).

25. Kuijk E, Kranenburg O, Cuppen E, Van Hoeck A. Common anti-cancer therapies induce somatic mutations in stem cells of healthy tissue. *Nature Communications* **13**, 5915 (2022).

26. Pich O*, et al.* Somatic evolution following cancer treatment in normal tissue. *Nature*, (2025).

27. Mitchell E, *et al.* The long-term effects of chemotherapy on normal blood cells. *Nature Genetics* **57**, 1684-1694 (2025).

28. Sánchez-Guixé M, *et al.* Origins of Second Malignancies in Children and Mutational Footprint of Chemotherapy in Normal Tissues. *Cancer Discov* **14**, 953-964 (2024).

29. Coombs CC, *et al.* Therapy-Related Clonal Hematopoiesis in Patients with Non-hematologic Cancers Is Common and Associated with Adverse Clinical Outcomes. *Cell stem cell* **21**, 374-382.e374 (2017).

30. Bolton KL, *et al.* Cancer therapy shapes the fitness landscape of clonal hematopoiesis. *Nat Genet* **52**, 1219-1226 (2020).

31. Hsu JI, *et al.* PPM1D Mutations Drive Clonal Hematopoiesis in Response to Cytotoxic Chemotherapy. *Cell stem cell* **23**, 700-713.e706 (2018).

32. Kahn JD, *et al.* PPM1D-truncating mutations confer resistance to chemotherapy and sensitivity to PPM1D inhibition in hematopoietic cells. *Blood* **132**, 1095-1105 (2018).

33. Kleiblova P, *et al.* Gain-of-function mutations of PPM1D/Wip1 impair the p53-dependent G1 checkpoint. *J Cell Biol* **201**, 511-521 (2013).

34. Chen Z, *et al.* Wip1 deficiency impairs haematopoietic stem cell function via p53 and mTORC1 pathways. *Nature Communications* **6**, 6808 (2015).

35. Zink F, *et al.* Clonal hematopoiesis, with and without candidate driver mutations, is common in the elderly. *Blood* **130**, 742-752 (2017).

36. Zhang L, Hsu JI, Goodell MA. PPM1D in Solid and Hematologic Malignancies: Friend and Foe? *Molecular Cancer Research* **20**, 1365-1378 (2022).

37. Abascal F, *et al.* Somatic mutation landscapes at single-molecule resolution. *Nature* **593**, 405-410 (2021).

38. Pharoah PDP, *et al.* PPM1D Mosaic Truncating Variants in Ovarian Cancer Cases May Be Treatment-Related Somatic Mutations. *J Natl Cancer Inst* **108**, (2016).

39. Swisher EM*, et al.* Somatic Mosaic Mutations in PPM1D and TP53 in the Blood of Women With Ovarian Carcinoma. *JAMA Oncology* **2**, 370-372 (2016).

40. Burocziova M, Danek P, Oravetzova A, Chalupova Z, Alberich-Jorda M, Macurek L. Ppm1d truncating mutations promote the development of genotoxic stress-induced AML. *Leukemia* **37**, 2209-2220 (2023).

41. Ruark E*, et al.* Mosaic PPM1D mutations are associated with predisposition to breast and ovarian cancer. *Nature* **493**, 406-410 (2013).

42. Herms A*, et al.* Self-sustaining long-term 3D epithelioid cultures reveal drivers of clonal expansion in esophageal epithelium. *Nature Genetics* **56**, 2158-2173 (2024).

43. Wenger A*, et al.* Extensive and differential platinum chemotherapy mutagenesis in children. *bioRxiv*, 2025.2010.2012.681852 (2025).

44. Boot A*, et al.* In-depth characterization of the cisplatin mutational signature in human cell lines and in esophageal and liver tumors. *Genome Research* **28**, 654-665 (2018).

45. Abby E*, et al.* Notch1 mutations drive clonal expansion in normal esophageal epithelium but impair tumor growth. *Nat Genet* **55**, 232-245 (2023).

Response to reviewers NG-A70761R

We are most grateful for the thoughtful critique of the revised manuscript. We respond to the outstanding comments below in blue.

Reviewer #1 (Remarks to the Author):

The authors have done a commendable effort to address the issues raised in the first review. As far as this reviewer is concerned, comprehensive, sufficient amount of clarification has been provided in response to the comments and questions by the panel of reviewers and in the text of the manuscript. In particular, the manuscript is supported by more robust statistical analyses, the reporting is comprehensive, the methodology better described, the mechanistic interpretation has been refined. All in all, the way the results are now framed makes more sense.

With all that said, there is a reminder comment regarding the very remarkable, almost puzzling result that there were no traces of mutational signatures of 5-FU, oxaliplatin or carboplatin (SBS25, 31 and 35) and only the CROSS samples witnessed a significant increase of the mutation burden. The authors postulate that this may reflect a selective loss of normal oesophageal cells damaged by these agents. In the response to reviewers the authors also refer to the possibility that clonal expansions after the CROSS regime can explain more mutations being detected in the corresponding samples due to an increase in the proportion of mutated epithelium without requiring foreign mutational processes: according to this hypothesis, we would expect that the clock like signatures + SBS16 in specific cases are predominantly due to low chemo exposed cells, with the higher CROSS burden being a result of a mixture of expanded clock-like mutations and maybe foreign mutations specific to ionizing radiation. Another possible explanation may be that, even under completely neutral selection, some endogenous mutational signatures, most prominently SBS5, may funnel heterogeneous sources of DNA damage as postulated in this preprint (https://www.biorxiv.org/content/10.1101/2025.08.28.672844v2 [biorxiv.org]).

As much as these explanations can make sense, in view of the technical difficulties to completely rule out the chemotherapy mutational footprint – and also for the sake of establishing sensitivity bounds due to the methodology in place – this reviewer suggests, at the discretion of the authors, a few more stringent tests that could be conducted as part of this analysis.

How to be extra confident that there are no traces of chemotherapy induced mutations? What would be the sensitivity bounds owing to fitting method of choice?

This reviewer suggests two potential extra avenues to completely clarify this point, insofar as this is one of the most striking observations of the study.

- To check whether the de novo signature extraction of choice is in agreement with an alternative method. By experience of our group, this reviewer would suggest looking into the

results of HDP (https://github.com/McGranahanLab/HDP_sigExtraction [github.com]) or MSA (https://gitlab.com/s.senkin/MSA [gitlab.com]) to confirm the consistency of the mapping of the mutation catalogs with mutational processes.

We thank the reviewer for their insightful comments and agree that additional signature extraction analysis by an alternative package is worthwhile. We attempted to run MsigHDP but encountered several software compatibility issues during execution. Specifically, we identified bugs in the SaveAnalysis function where a missing infer.rownames = TRUE argument in an internal ICAMS::as.catalog call caused the pipeline to fail at the output stage, and a separate issue in CombineChainsAndExtractSigs where signatures meeting the user-specified high.confidence.prop threshold were incorrectly classified as low-confidence. These issues appear to reflect an incompatibility between the current mSigHdp release and the ICAMS 4.0.0 API. We were able to partially work around these issues but were unable to reliably extract high-confidence signatures from our cohort.

For the **nicolaroberts/hdp** package (Forked by McGranahanLab above), installation failed due to a C linkage error (multiple definition of DEBUG) introduced by stricter behaviour in modern linkers (gcc/ld), rendering the package uninstallable in our R environment without non-trivial modification of the package source code.

Given these technical barriers, we instead compared the SigProfilerExtractor results with **MutationalPatterns** (v3.22.0)[1], an independent NMF-based approach available through Bioconductor, using the maximum number of signatures with stability above 0.8. Analysis of all treatment groups (CROSS, ECX, EOX, FLOT and NT) revealed 5 deconvolved signatures (SBS1, SBS5, SBS16, SBS40 and SBS8) (**Reviewer Figure 1**). SBS8 is currently of unknown aetiology and was found in 62/69 patients, in the NT and treatment samples alike ((CROSS (100%), ECX (94%), EOX (92%), FLOT (71%), NT (86%)). Thus, both MutationalPatterns and SigProfilerExtractor fail to identify signatures linked to chemotherapy in any group.

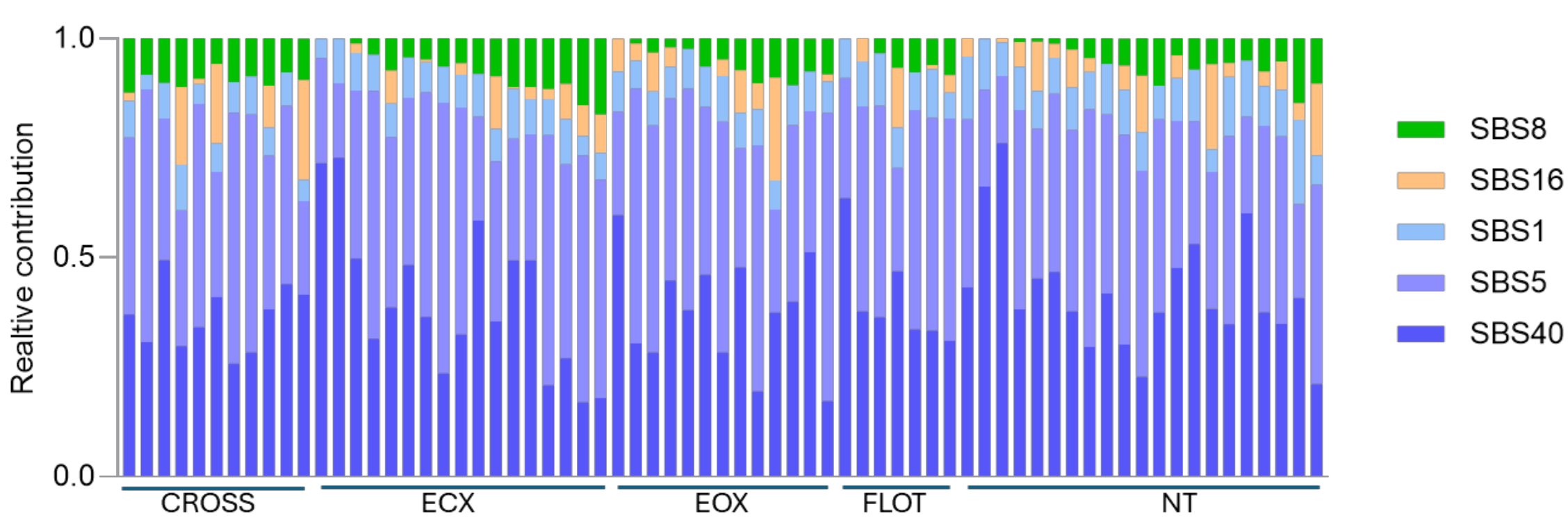

Reviewer Figure 1 Relative mutational contribution to COSMIC deconvolved signatures determined by Mutational Patterns. CROSS n=11, ECX n=17, EOX n=13, FLOT n=7, NT n=21 patients. SBS1(light blue), SBS5 (mid blue), SBS40 (dark blue), SBS 16 (orange), SBS8 (green).

The appearance of SBS8 is surprising as this has not been seen in previous studies of somatic mutations in the oesophagus[2-4]. To further assess the quality of signature attributions, we used MutationalPatterns to call signatures across a UK cohort of normal esophagus from transplant donors with no history of cancer (**Reviewer Figure 2**)[2]. Four signatures deconvolving into SBS1, SBS16, SBS40 and SBS30 were present in all 31 UK, samples (**Reviewer Figure 2**). No SBS5 was observed. SBS30 is associated with inactivating NTHL1 mutations. Seven of the 31 UK patients had been whole genome sequenced and none showed any mutations with NTHL1.

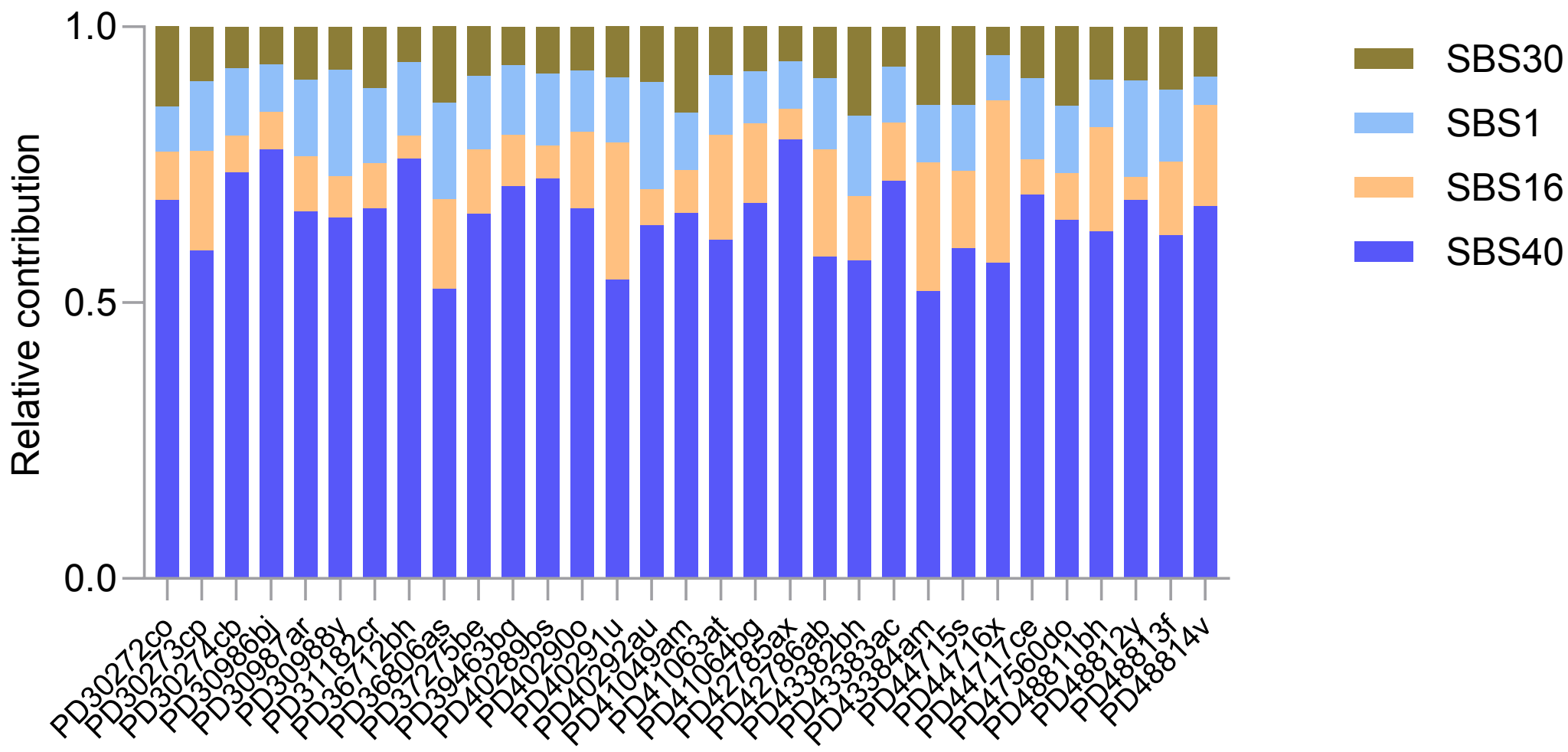

Reviewer Figure 2 Relative mutational contribution to COSMIC deconvolved signatures determined by Mutational Patterns. UK donor samples, n=31. SBS1(light blue), SBS40 (dark blue), SBS 16 (orange), SBS30 (brown).

We conclude that whilst MutationalPatterns does not reveal a chemotherapy signature in treated patients, the results we obtain from both the cancer patients in this study and the UK normal cohort are inconsistent with previous reports. We have therefore not included this analysis within the manuscript at present, though we would be happy to do so at the reviewer's request.

- In order to be extra confident of the presence or absence of a footprint of a certain foreign signature, it might also be useful to conduct a likelihood test, whereby a collection of baseline signatures representing the typical mutational processes operative in the oesophagus can be used, e.g. SBS1, 5, 40a, 16, to test whether the reconstruction would significantly improve by including an additional query signature (in this case, treatment-associated) like SBS25, 31, 35. This can be accomplished by e.g. an off-the-shelf method like mSigAct

(https://github.com/steverozen/mSigAct [github.com]).

As suggested by the reviewer we have also run the mSigAct method over the Nanoseq data (**Table 1**). Many of the treatment patients (CROSS, ECX, EOX and FLOT) did show a significantly improved fit with the addition of the chemotherapy signatures SBS25, SBS31 and SBS35 to a baseline signature. However, this was also true for both the non-treatment (NT) patients and a cohort of transplant donors from the UK (from [5]), none of whom had been exposed to chemotherapy.

	**Total patients**	**Patients with any chemotherapy signature (SBS25, 31 or 35)**	**SBS25**	**SBS31**	**SBS35**
CROSS	11	7 (64%)	7 (64%)	5 (45%)	5 (45%)
ECX	17	11 (65%)	8 (47%)	6 (35%)	9 (53%)
EOX	13	4 (31%)	3 (23%)	1 (8%)	0
FLOT	7	6 (86%)	3 (42%)	5 (71%)	5 (71%)
NT	21	8 (38%)	7 (33%)	4 (19%)	5 (24%)
UK*	31	3 (10%)	1 (3%)	2 (6%)	0

Table 1: Proportion of patients showing significant improved fitting of chemotherapy signatures SBS25, SBS31 or SBS35 to baseline signature containing SBS1, SBS5, SBS16 and SBS40a using mSigAct. * Nanoseq from esophagus from 31 transplant donors from [5].

We have not included this analysis in the manuscript at present but can add it at the reviewers' request.

We agree with the reviewer lack of robust chemotherapy signatures in treated esophagus is surprising as these have been identified in chemotherapy exposed tumours, including eosophageal adenocarcinoma, and in normal tissues other than esophagus[6-12]. As the neoadjuvant dosing regimens in the patient cohort involve a

maximum of 4 cycles it is possible that insufficient mutations are fixed relative to preexisting mutations to enable therapy associated SBS signatures to be detected. Sequencing of esophagus from patients who are on longer treatment schedules may help to resolve this question.

We have amended the text as follows

'The lack of detectable mutational signatures associated with chemotherapy in esophagus from treated individuals was notable. This may reflect loss of epithelial cells that acquire sufficient chemotherapy-induced damage by cell death or by differentiation and shedding of cells that are predominantly *TP53* wild type. It is also possible that the short duration of neoadjuvant treatment does not fix sufficient mutations in the tissue for detection of chemotherapy induced signatures.' Lines 525-530.

Reviewer 2

Reviewer #2 (Remarks to the Author):

The authors have responded quickly and clearly worked hard. I am very happy with their responses, and in particular am pleased to see supplementary note 1. I would be happy to see this accepted if the authors address two minor points that I'll raise in summary at the end.

I'll go through my thoughts on the author responses as I formatted my initial review and unless I refer to something specifically, it should be taken that I am satisfied that the authors have sufficiently addressed any issues.

Major point 1) Mutation signature analysis not robust enough

This is a much stronger analysis and feel that their efforts of mutation signature deconvolution are defensible. I am still surprised that there are no chemo or smoking signatures, but that's the data and I accept the author's arguments as to why this is. Their reason for not using DBS to try and infer exposure-based signatures is also very fair and the outcome of the simulations is acceptable, especially as there are no treatment-specific signature.

I like the inclusion of the SBS288 signatures and their fitting as supplementary figures. The authors very graciously handled my misunderstanding as to the extent of genome coverage of nanoseq and made a good attempt to link mutational processes therein with their panel-based sequencing. Though the outcome is not very satisfying, I am happy that to push for anything else is beyond the scope of their efforts.

Thank you for the positive assessment of the revisions which have significantly improved the paper.

Major point 2) Confounding sources of variation throughout and superficial statistical

analysis

Reviewer figs 6 and 8 are not insightful and do not do enough to convey the impact of bias on the analyses through a lack of statistical analysis, though this does not impact the manuscript. In spite of this, supplementary note 1 does do a good job and I am happy that there are no confounding aspects of patient cohorts in their findings. To this point, I took the VAF data for TP53 SNVs from Table 5 and ran a mixed effects model that accounts for patient and sample as mixed effects to assess the impact of treatment, smoking, gender, disease state and location on VAF. My analysis agrees with the findings of the authors, that only the CROSS treatment type shows a significant effect on TP53 clone size (p = 0.0349).

Thank you for suggesting these analyses which have significantly improved the manuscript.

The author's use Kendall's concordance test to define significant differences the proportion of types of mutations, but if I'm not mistaken, this uses ranks as input and therefore isn't appropriate for this purpose.

There are multiple possible tests to assess the distributions of mutation types between treatments shown in extended data Fig. 5 c &d. This data is not normally distributed (including residuals) so it is best to apply a non-parametric test such as Freeman-Fisher-Haton (a generalization of chi-square), the Friedman test, or Kendall's concordance test. The proportion of mutation types appears to be very similar between treatment groups and therefore we felt that a Kendall's concordance test would be the most appropriate to capture the degree of similarity (i.e. agreement or concordance) between the distributions, as opposed to the other tests which measure possible differences.

As the Reviewer correctly points out ranked data is used by the Kendall's test. However, this test also accepts the input of continuous data, by ranking of the data as a first step, in a similar manner to other non-parametric tests. We also note Kendall's concordance test is typically used in ecological environmental studies in a similar context as ours[13].

' Kendall's concordance test was used to test for similarity between the distributions shown in Extended Data Figures 5c and 5d, by ranking the continuous values [13]. ' Lines 822 to 823.

We have not included the Freeman-Fisher-Haton and Friedman tests, which also show no significant difference between the distributions but can do so if this is felt necessary.

In their attempt to compare neutral mutations between panel-based sequencing and nanoseq, I appreciate that the inherent bias in each of the methods makes it difficult to derive comparable mutational process. Despite this, the authors attempt mutation signature fitting of the panel-based data, and while broadly similar, there are a

couple of points that stand out: SBS4 is present, with nonsmokers making up 2/7 with considerable sbs4 burden (Pd49362, Pd49363). The authors state that there are 5 patients with sbs4, but there are 7. There are inconsistencies here that don't gel with the narrative in the manuscript, but I appreciate that the comparison of the two data types and use of signature fitting on pull-downs of the genome is not necessarily a fair or very accurate way of comparing the two sequencing methods. I appreciate their effort and will leave it at that.

Major point 3) Inclusion of FLOT and in vitro experiment

I'm happy with the Author's responses. Given the level of detail that they go into with regard to Nfe2l2, it's fair to say that they have attempted this experiment and that I can't ask for more. The lack of this makes the narrative a bit nonsensical, but the results are sufficiently worthwhile that most readers won't be bothered by it.

Minor points:

I can see the EGA accessions now, though the github repository has not been updated despite the author's response. It says the last update was 13.07.25, therefore it was not rerun. Was an update pushed? In their attempt to compare neutral mutations between panel-based sequencing and nanoseq, I appreciate that the inherent bias in each of the methods makes it difficult to derive comparable mutational process. Despite this, the authors attempt mutation signature fitting of the panel-based data, and while broadly similar, there are a couple of points that stand out: SBS4 is present, with nonsmokers making up 2/7 with considerable sbs4 burden (Pd49362, Pd49363). The authors state that there are 5 patients with sbs4, but there are 7. There are inconsistencies here that don't gel with the narrative in the manuscript, but I appreciate that the comparison of the two data types and use of signature fitting on pull-downs of the genome is not necessarily a fair or very accurate way of comparing the two sequencing methods. I appreciate their effort and will leave it at that.

Major point 3) Inclusion of FLOT and in vitro experiment

I'm happy with the Author's responses. Given the level of detail that they go into with regard to Nfe2l2, it's fair to say that they have attempted this experiment and that I can't ask for more. The lack of this makes the narrative a bit nonsensical, but the results are sufficiently worthwhile that most readers won't be bothered by it.

Minor points:
I can see the EGA accessions now, though the github repository has not been updated despite the author's response. It says the last update was 13.07.25, therefore it was not rerun. Was an update pushed?

Apologies, the updates to the github repository had been incorrectly pushed. This has now been corrected.

Summary:
I would like to see the github repository actually updated and a justification for, or alteration of, use of Kendall's concordance test.

Thank you, please see comments above.

Reviewer #3 (Remarks to the Author):

The authors have responded constructively to my comments and have substantially improved the structural and protein-level interpretation. In particular, the revised manuscript now defines the structural datasets used, separates 1D clustering from structural enrichment analyses, provides clearer descriptions of the null models and statistical tests, expands the dN/dS reporting, and moderates several of the mechanistic conclusions. I am satisfied that the my main concerns have been addressed.

Thank you for the constructive critique that has significantly improved the paper.

References

1 Manders, F. *et al.* MutationalPatterns: the one stop shop for the analysis of mutational processes. *BMC Genomics* **23**, 134 (2022). https://doi.org/10.1186/s12864-022-08357-3

2 King, C. *et al.* Somatic mutant selection is altered by prior NOTCH1 mutation in aging esophageal epithelium. *bioRxiv*, 2024.2009.2026.615123 (2024). https://doi.org/10.1101/2024.09.26.615123

3 Martincorena, I. *et al.* Somatic mutant clones colonize the human esophagus with age. *Science (New York, N.Y.)* **362**, 911-917 (2018). https://doi.org/10.1126/science.aau3879

4 Yokoyama, A. *et al.* Age-related remodelling of oesophageal epithelia by mutated cancer drivers. *Nature* **565**, 312-317 (2019). https://doi.org/10.1038/s41586-018-0811-x

5 King, C. *et al.* Somatic mutant selection is altered by prior NOTCH1 mutation in aging esophageal epithelium. *bioRxiv*, 2024.2009.2026.615123 (2026). https://doi.org/10.1101/2024.09.26.615123

6 Murugaesu, N. *et al.* Tracking the genomic evolution of esophageal adenocarcinoma through neoadjuvant chemotherapy. *Cancer Discov* **5**, 821-831 (2015). https://doi.org/10.1158/2159-8290.Cd-15-0412

7 Abbas, S. *et al.* Mutational signature dynamics shaping the evolution of oesophageal adenocarcinoma. *Nature Communications* **14**, 4239 (2023). https://doi.org/10.1038/s41467-023-39957-6

8 Pich, O. *et al.* The mutational footprints of cancer therapies. *Nat Genet* **51**, 1732-1740 (2019). https://doi.org/10.1038/s41588-019-0525-5

9 Pich, O. *et al.* Somatic evolution following cancer treatment in normal tissue. *Nature* (2025). https://doi.org/10.1038/s41586-025-09792-4

10 Mitchell, E. *et al.* The long-term effects of chemotherapy on normal blood cells. *Nature Genetics* **57**, 1684-1694 (2025). https://doi.org/10.1038/s41588-025-02234-x

11 Wang, Y. *et al.* APOBEC mutagenesis is a common process in normal human small intestine. *Nat Genet* **55**, 246-254 (2023). https://doi.org/10.1038/s41588-022-01296-5

12 Wenger, A. *et al.* Extensive and differential platinum chemotherapy mutagenesis in children. *bioRxiv*, 2025.2010.2012.681852 (2025). https://doi.org/10.1101/2025.10.12.681852
13 Legendre, P. Species associations: the Kendall coefficient of concordance revisited. *Journal of Agricultural, Biological, and Environmental Statistics* **10**, 226-245 (2005). https://doi.org/10.1198/108571105X46642
